# Synergistic Regulation of Nucleation and Interfacial Chemistry for Energy-Dense and Durable Anode-Free Na Batteries

**DOI:** 10.1021/jacs.6c02019

**Published:** 2026-04-08

**Authors:** Yongling An, Zhihao Pei, Jiarui Yang, Deyan Luan, Xiong Wen David Lou

**Affiliations:** Department of Chemistry, 53025 City University of Hong Kong, 83 Tat Chee Avenue, Kowloon, Hong Kong 999077, China

## Abstract

Anode-free Na batteries are promising for achieving ultimate high energy density yet experience unsatisfactory cycling stability largely stemming from inhomogeneous Na deposition and an unstable solid electrolyte interphase (SEI). Herein, we report a multifunctional interface layer composed of Sn nanodots and ZnF_2_ nanosheets on a lightweight Al current collector (Sn–ZnF_2_/Al) to homogenize Na growth and stabilize the SEI layer. The abundant sodiophilic Sn and ZnF_2_ sites reduce Na nucleation energy barriers and uniformize the electric field distribution, further guiding homogeneous Na growth. Moreover, the in situ formed NaF-rich SEI enhances interfacial stability and serves as a protective layer against side reactions. Consequently, synergistic performance improvement is realized with highly reversible Na plating and stripping of around 99.98% at 8.0 mA cm^–2^ and 1.0 mAh cm^–2^ over 4440 cycles. Remarkably, an energy-focused anode-free pouch cell (Sn–ZnF_2_/Al//Na_3_V_2_O_2_(PO_4_)_2_F) exhibits an energy density of 442.9 Wh kg^–1^ (calculated based on active materials of both electrodes) and 80.37% capacity retention after 220 cycles. Meanwhile, a lifetime-focused anode-free pouch cell (Sn–ZnF_2_/Al//Na_4_Fe_3_(PO_4_)_2_P_2_O_7_) achieves ultrastable cyclability with 80.59% capacity retention over 2600 cycles. This work offers a potentially universal strategy for the development of anode-free Na batteries.

## Graphical abstract

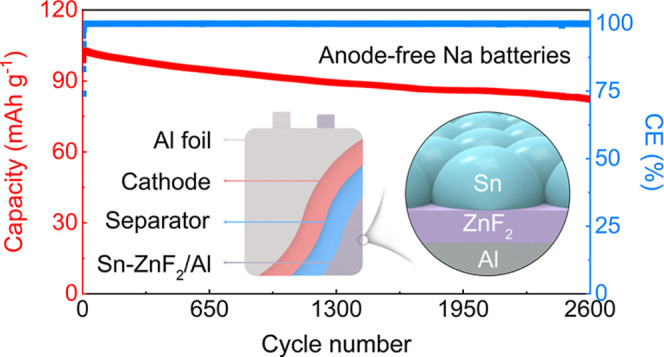

## 1 Introduction

Na-ion batteries are regarded as cost-effective energy storage technologies because of the abundance of Na resources.^1,2^ However, the large ionic radius and mass of Na ions, which function as charge carriers, limit their energy density.^3,4^ An anode-free architecture, which employs a pre-sodiated cathode paired directly with a current collector, represents an effective approach to simultaneously minimize cost and maximize energy density.^5,6^ While pursuing the highest energy density, this device struggles to achieve long-term cycling stability due to the limited Na source.^7,8^ Therefore, achieving high reversibility of Na deposition/dissolution is pivotal for enhancing the performance of anode-free Na batteries (AFNBs).^9,10^

The current collector is critical for Na nucleation and initial growth, which determines the Coulombic efficiency (CE) and ultimately affects battery performance.^11,12^ A current collector with high Na affinity facilitates homogeneous Na plating and suppresses dendrite growth.^13,14^ A viable method is to engineer sodiophilic materials [e.g., metals (Au,^15^ Ag,^16^ Sn,^17^ Zn^18^), oxides (ZnO,^5^ SnO_2_ ^19^), carbon-based materials (amorphous and graphitic carbon,^3^ Ketjenblack,^20^ heteroatom-doped carbon^9^), compounds (fluorinated Al,^21^ Cu_3_P,^22^ Bi–N_3_S_1_ ^23^), etc.] onto inherently sodiophobic current collectors. Such engineered systems help sustain high energy density and offer improved modulation potential.^23,24^ Controlling Na plating is thus key to CE, which significantly affects the performance of AFNBs.^25,26^ However, the spontaneously formed solid electrolyte interphase (SEI) between deposited Na metal and electrolyte has poor chemical and mechanical stability.^27,28^ It is prone to cracking during repeated volume changes, which continuously depletes active Na and electrolytes and consequently lowers CE.^29,30^ Therefore, under the harsh conditions of anode-free systems, it is crucial to simultaneously tackle the two closely intertwined issues of uniform nucleation and SEI stability through an integrated design approach.

In this work, we design a dense and multifunctional interface layer composed of Sn nanodots and ZnF_2_ nanosheets on a lightweight Al current collector (Sn–ZnF_2_/Al) for AFNBs. The abundant active sites provided by Sn and ZnF_2_ significantly reduce the Na nucleation barrier, subsequently guiding uniform Na deposition. Moreover, the in situ formed NaF-rich SEI during Na plating enhances interfacial stability and suppresses side reactions. Accordingly, the Sn–ZnF_2_/Al host exhibits a high reversibility of Na deposition and dissolution with an average CE of around 99.98% at 8.0 mA cm^–2^ and 1.0 mAh cm^–2^ over 4440 cycles. Remarkably, the Sn–ZnF_2_/Al–Na electrode delivers long-term cycling stability for 1000 h at 10.0 mA cm^–2^ and 10.0 mAh cm^–2^. In addition, an energy-focused anode-free Sn–ZnF_2_/Al//Na_3_V_2_O_2_(PO_4_)_2_F (NVOPF) pouch cell exhibits an energy density of 442.9 Wh kg^–1^ (calculated based on the active materials of both electrodes) and 80.37% capacity retention after 220 cycles. Meanwhile, a lifetime-focused anode-free Sn–ZnF_2_/Al//Na_4_Fe_3_(PO_4_)_2_P_2_O_7_ (NFPP) pouch cell exhibits ultrastable cyclability with 80.59% capacity retention over 2600 cycles.

## 2 Results and Discussion

### 2.1 Preparation and Characterization of Sn–ZnF_2_/Al

Sn–ZnF_2_/Al is fabricated by electrodeposition and galvanic displacement methods (Figure 1A). The pristine Al foil is characterized by the field-emission scanning electron microscopy (FESEM) images, energy-dispersive X-ray (EDX) spectrum, and X-ray diffraction (XRD) pattern (Figures S1 and S2). By an electrodeposition approach, Zn nanosheets are deposited onto an Al foil surface (Zn/Al, Figures 1B,C and S3). The EDX spectrum and XRD pattern show the characteristic peaks of Zn and Al (Figure S4), manifesting the Zn deposition on the Al current collector.

**Figure 1:**
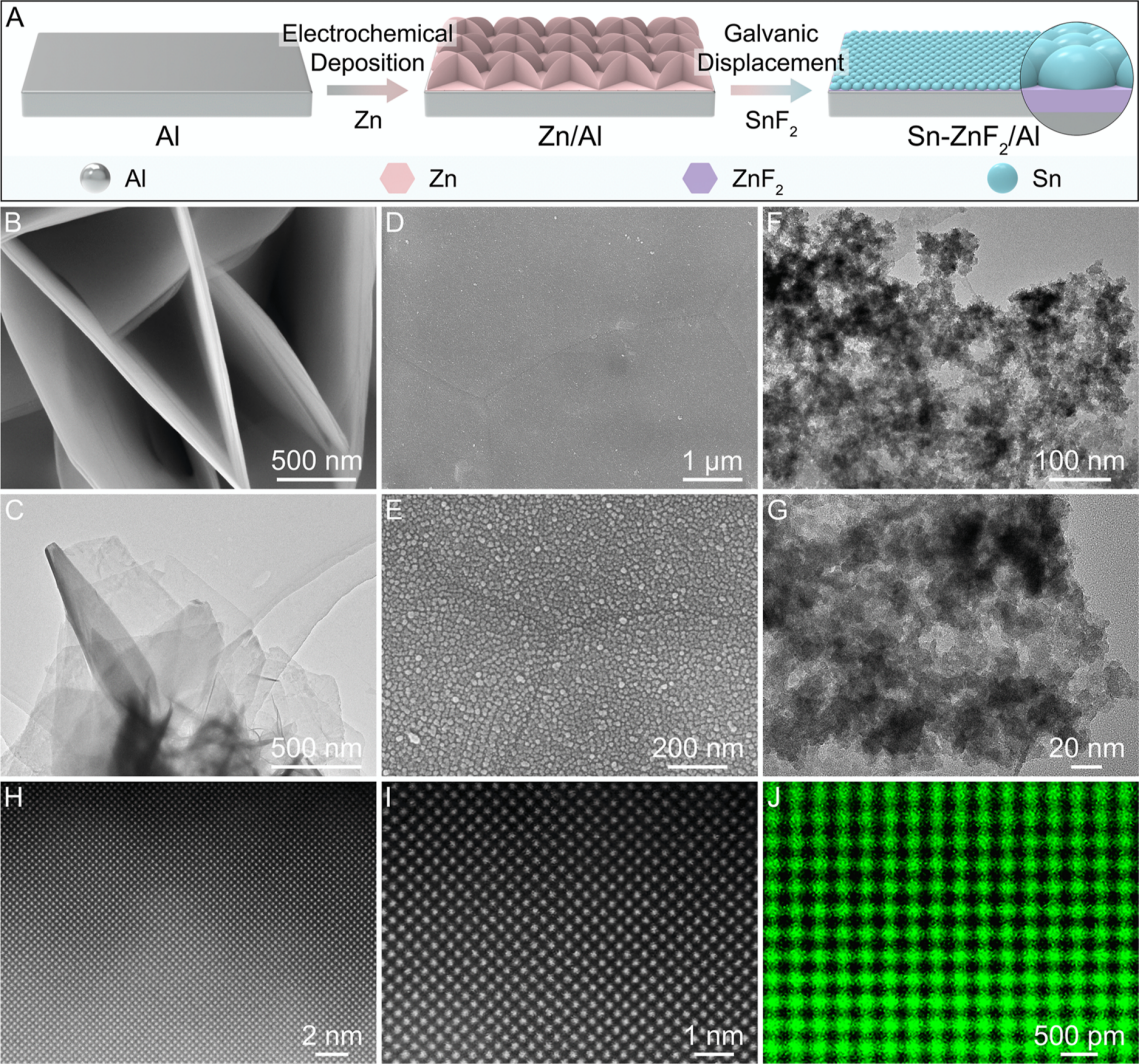
Fabrication and morphology of Sn–ZnF_2_/Al. (A) Schematic illustration of the synthesis process of Sn–ZnF_2_/Al. (B) FESEM and (C) TEM images of Zn/Al. (D,E) FESEM, (F,G) TEM, and (H,I) HAADF-STEM images and (J) corresponding elemental mapping of Sn–ZnF_2_/Al.

Zn nanosheets are subsequently converted into uniform Sn nanodots and ZnF_2_ nanosheets by a galvanic displacement process (Figure 1D,E). The thickness of the Sn−ZnF_2_ layer is about 55 nm (Figure S5). The XRD pattern of Sn–ZnF_2_/Al shows the characteristic peaks of Sn, and the EDX result proves the existence of Zn, F, and Sn elements (Figure S6). In the X-ray photoelectron spectroscopy (XPS) Zn 2p spectra, two characteristic peaks of Zn 2p_1/2_ and Zn 2p_3/2_ are detected (Figure S7). Compared to that of Zn/Al, the Zn 2p_1/2_ and Zn 2p_3/2_ peaks of Sn–ZnF_2_/Al exhibit a slight shift, which is attributed to the formation of ZnF_2_.^31^ In the F 1s spectra, the F signal further verifies the formation of ZnF_2_. These results collectively prove that Zn reacts with SnF_2_ to form Sn and ZnF_2_. Transmission electron microscopy (TEM) images disclose that Sn nanodots are grown on ZnF_2_ nanosheets (Figure 1F,G). Elemental mapping exhibits a homogeneous distribution of Sn, Zn, and F elements throughout the Sn–ZnF_2_/Al structure (Figure S8), consistent with the architecture of Sn nanodots dispersed on ZnF_2_ nanosheets. High-angle annular dark-field scanning TEM (HAADF-STEM) and mapping results of Sn–ZnF_2_/Al show clear lattice fringes of Sn (Figures 1H–J and S9),^32^ which are consistent with the XRD results.

The effect of the H_2_O content in the solvent medium on the morphology of Sn–ZnF_2_/Al is systematically investigated (Figure 2A). At an H_2_O content of 0%, most Zn nanosheets react, leaving a small fraction unreacted (Figure S10). When H_2_O content increases to 5.0%, only a limited number of Zn nanosheets remain, with the rest having almost completely reacted (Figures 2B,E and S11). Further increasing H_2_O content to 10.0% leads to the formation of uniform Sn nanodots and ZnF_2_ nanosheets (Figure 2C,F). When the H_2_O content reaches 15.0%, the Sn nanodots agglomerate, bringing about a significant deterioration in their distribution homogeneity (Figure 2D,G). Increasing the H_2_O content to 20.0% results in the formation of more inhomogeneous and larger Sn nanoparticles (Figure S12). In H_2_O environments, Sn^2+^ ions tend to hydrolyze readily, which may precipitate and disturb the uniformity of the displacement reaction.^33^ This explains the severe aggregation observed at high H_2_O contents. At an optimal H_2_O content, hydrolysis is moderated, allowing controlled Sn^2+^ ion reduction and uniform Sn nanodot formation, whereas in dimethyl sulfoxide (DMSO) environments, the displacement reaction proceeds too slowly, leading to incomplete conversion of Zn nanosheets.^33^ The addition of H_2_O accelerates the reaction to a favorable rate. To achieve an optimal reaction rate, a controlled amount of H_2_O is introduced into DMSO as an auxiliary solvent. In this work, a homogeneous and compact interface layer is achieved at an H_2_O content of 10.0% in the solvent medium. This interface layer composed of sodiophilic Sn and ZnF_2_ sites on an Al current collector can lower the Na nucleation energy barrier and homogenize the electric field distribution, thus guiding homogeneous Na growth. In contrast to other current collectors, Al foil is preferred in terms of weight and can function as a current collector in Na batteries.

**Figure 2:**
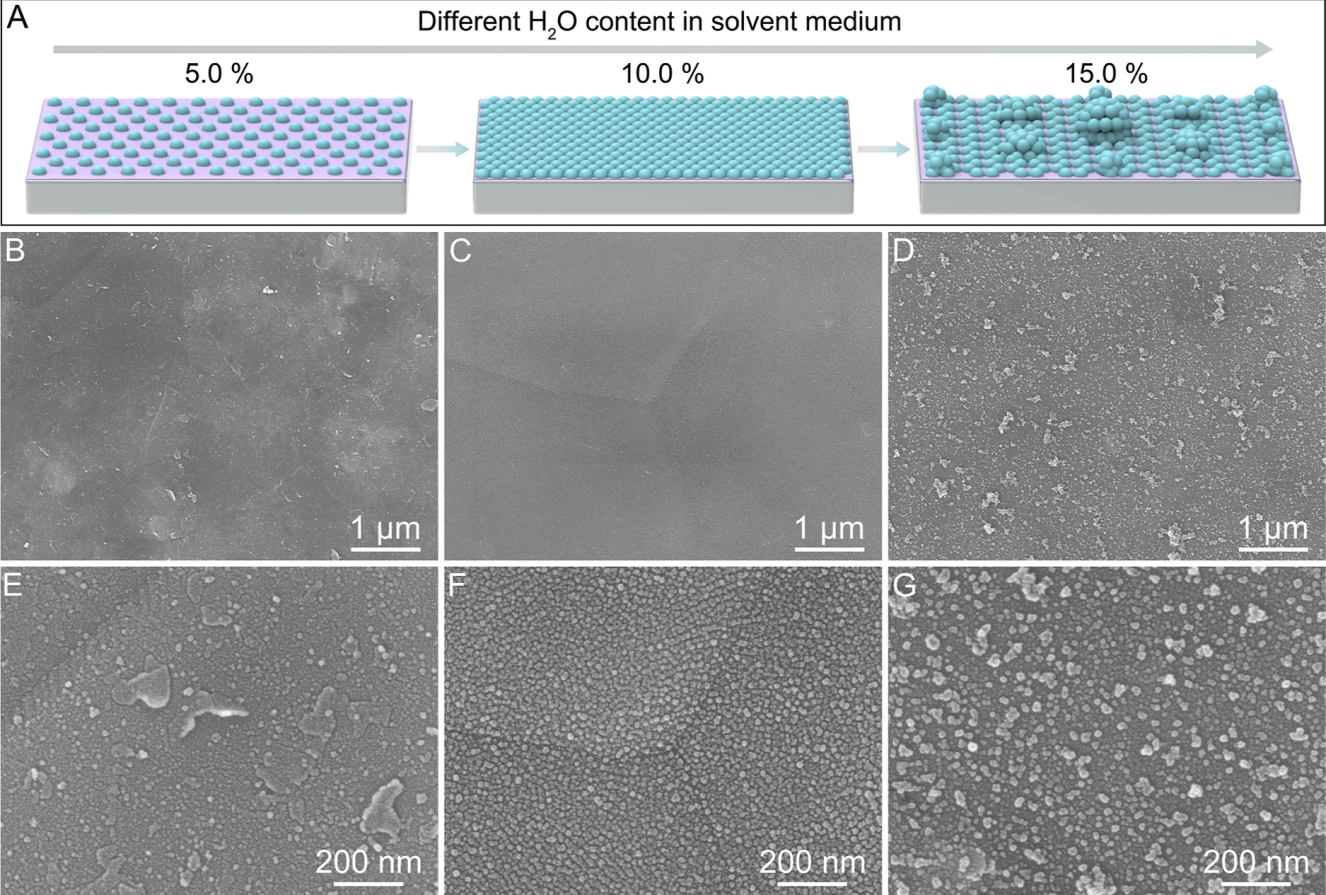
Morphological evolution of Sn–ZnF_2_/Al. (A) Schematic representation of the morphological evolution of Sn–ZnF_2_/Al. (B–G) SEM images of Sn–ZnF_2_/Al synthesized with different H_2_O content in a solvent medium: (B,E) 5.0%, (C,F) 10.0%, and (D,G) 15.0%.

### 2.2 Na Plating Behavior

Density functional theory calculations clarify the role of individual components in Sn–ZnF_2_/Al (Figures S13 and S14). Sodiophilicity, defined as the capability of a substrate to adsorb and bond Na, is quantitatively indicated by its binding energy.^34–36^ As shown in Figure 3A, both Sn and ZnF_2_ exhibit high binding energies, thus confirming their sodiophilic nature.^5^ Furthermore, interfacial charge density analysis displays significant charge transfer at the interface of Sn and ZnF_2_, revealing a strong interaction (Figure 3B). This strong affinity effectively mitigates the accumulation of deposited Na within Sn–ZnF_2_/Al, thereby suppressing the growth of Na dendrites.

**Figure 3:**
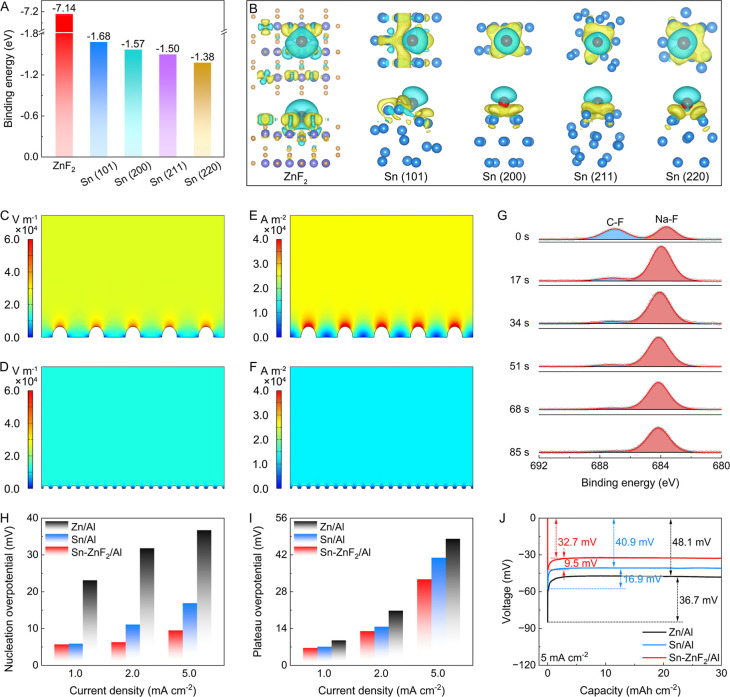
Stabilization mechanism of Na plating and electrochemical results of Sn–ZnF_2_/Al. (A) Binding energies of Na with Sn and ZnF_2_. (B) Charge-density models of Na-adsorbed Sn and ZnF_2_. Simulations of the distribution of (C,D) electric field and (E,F) current density on (C,E) Al and (D,F) Sn–ZnF_2_/Al. (G) F 1s spectra of the cycled Sn–ZnF_2_/Al electrode after etching for different times. (H,I) Nucleation and plateau overpotentials for various hosts at current densities of 1.0, 2.0, and 5.0 mA cm^–2^. (J) Voltage-capacity profiles of Na plating on different hosts at 5.0 mA cm^–2^.

Finite element simulation is performed to investigate the distribution of the electrolyte concentration, current density, and electric field intensity within the Sn–ZnF_2_/Al structure. Regions with significantly enhanced electric fields are observed on the uneven surface of the Al current collector (Figure 3C), which promotes the generation of Na dendrites.^37^ In addition, nonuniform electrolyte concentration and current density across the Al current collector are detected (Figures 3E and S15), suggesting a high likelihood of accelerated dendrite growth. By contrast, the Sn–ZnF_2_/Al host exhibits a uniform electric field distribution during plating (Figure 3D), which is attributable to preferential Na deposition. For Sn–ZnF_2_/Al, Na deposition occurs on the surfaces of sodiophilic components, leading to a reduced current density and dispersed ion flux (Figure 3F). Furthermore, the Sn–ZnF_2_/Al also homogenizes the distribution of the electrolyte concentration (Figure S15). Collectively, these simulation results suggest that Sn–ZnF_2_/Al effectively uniformizes the distributions of current density, electrolyte concentration, and electric field intensity, thereby supporting the long-term cyclability of cells.

SEI plays a critical role in governing Na deposition behavior.^28,38^ XPS depth profiling of cycled Sn–ZnF_2_/Al elucidates its SEI composition (Figure 3G). With different sputtering times from 0 s to 85 s, the characteristic peak of NaF is observed, indicating that the SEI is dominated by NaF. The NaF-rich SEI contributes to improving interfacial stability and achieving long-term cycling.^6,27,39^ Initial Na nucleation plays a significant role in controlling the quality of Na deposition.^40,41^ Comparative analysis of overpotentials at various current densities reveals the superiority of the Sn–ZnF_2_/Al host. Prepared via a similar process, Sn/Al serves as a control (Figure S16). The Sn–ZnF_2_/Al host exhibits the lowest nucleation overpotential across all tested current densities (Figures 3H and S17), indicating a reduced Na nucleation barrier. Furthermore, its plateau overpotential is consistently lower than that of other hosts (Figures 3I and S17), suggesting facilitated Na^+^ ion transport kinetics. For example, the Sn–ZnF_2_/Al host exhibits a nucleation overpotential of 9.5 mV and a plateau overpotential of 32.7 mV, both lower than those of the Sn/Al and Zn/Al counterparts (Figure 3J). This enhanced performance stems from its abundant sodiophilic Sn and ZnF_2_ sites, which lower the nucleation energy barrier and guide uniform Na deposition.

Na plating behavior on various hosts is examined to prove the advantage of Sn–ZnF_2_/Al (Figures S18–S20). Substantial Na dendrites form on the Zn/Al surface at a deposition capacity of 10 mAh cm^–2^, posing potential safety risks. In contrast, Na deposition is significantly more uniform on both Sn/Al and Sn–ZnF_2_/Al hosts. When the Na plating capacity reaches 20 mAh cm^–2^, the Sn–ZnF_2_/Al host exhibits a smooth surface morphology. This compact and uniform deposition persists at a high capacity of 30 mAh cm^–2^. The enhanced regulation of Na deposition on the Sn–ZnF_2_/Al host can be attributed to its sufficient sodiophilic Sn and ZnF_2_ sites, which reduce the nucleation energy barrier and promote uniform Na deposition.

### 2.3 Electrochemical Performance

Na plating/stripping efficiency, which is critical for the performance in an anode-free cell,^42^ is investigated. In Figures 4A and S21, the Sn–ZnF_2_/Al host exhibits the lowest onset voltage for Na deposition and dissolution among all of the tested hosts. High current density suggests the presence of more active sodiophilic sites, implying rapid electrochemical reaction kinetics.^9,43^ Besides, the Sn–ZnF_2_/Al host demonstrates a significantly longer cycling life and good reversibility compared to the Sn/Al and Zn/Al hosts (Figures 4B and S22). Specifically, an average CE of 99.95% over 1800 cycles is obtained by the Sn–ZnF_2_/Al host at 1.0 mA cm^–2^ and 1.0 mAh cm^–2^. In comparison, the Sn/Al host shows a relatively low average CE of 99.87% for 986 cycles, while the Zn/Al host experiences severe fluctuations for only 300 cycles. Electrochemical impedance spectroscopy (EIS) reveals a small charge transfer resistance of the Sn–ZnF_2_/Al host (Figure S23), indicating the fast reaction kinetics. FESEM images show a dense and uniform surface of the Sn–ZnF_2_/Al host after cycling (Figure S24). Figures 4C and S25–S36 show CEs on Sn–ZnF_2_/Al under different testing conditions. At 8.0 mA cm^–2^ and 1.0 mAh cm^–2^, it maintains an average CE of 99.98% after 4440 cycles. Such a high CE is consistently retained with improvement in both areal capacity and current density (Table S1). For instance, average CEs of 99.96% and 99.98% are achieved at conditions of 8.0 mA cm^–2^/10.0 mAh cm^–2^ and 10.0 mA cm^–2^/8.0 mAh cm^–2^, respectively. The performance of Sn–ZnF_2_/Al is comparable to that of previous works under identical conditions (Table S2).

**Figure 4:**
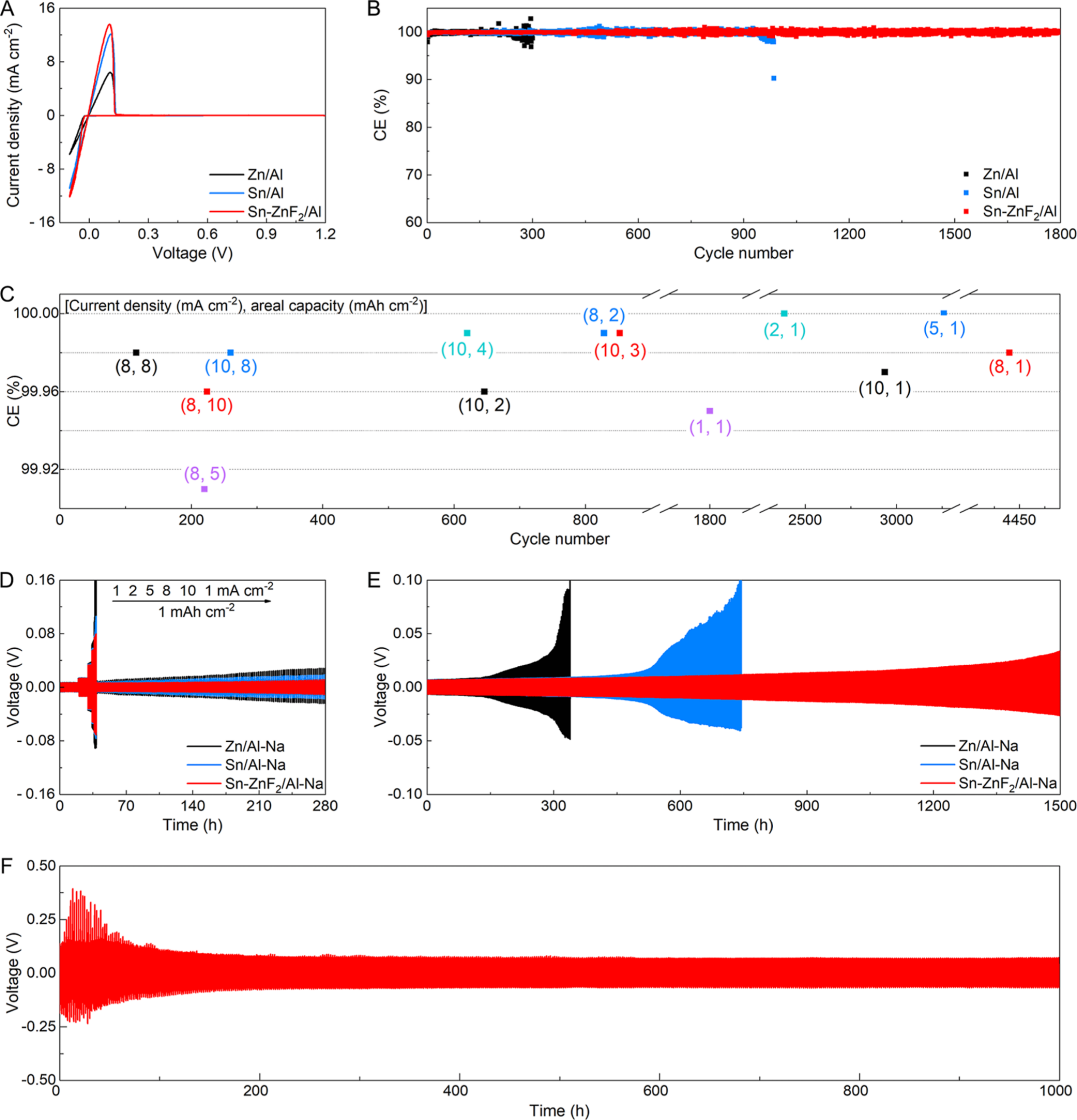
Na plating and stripping performance of Sn–ZnF_2_/Al. (A) CV profiles and (B) CE plots of various hosts. (C) CEs on the Sn–ZnF_2_/Al host at various areal capacities and current densities. (D) Rate capability and (E) cycling performance of different electrodes. (F) Cycling stability of Sn–ZnF_2_/Al–Na at 10 mA cm^–2^ and 10 mAh cm^–2^.

The cyclability of Zn/Al–Na, Sn/Al–Na, and Sn–ZnF_2_/Al–Na is probed. As the rate improves from 1.0 to 10.0 mA cm^–2^, the Sn–ZnF_2_/Al–Na displays excellent rate capability (Figure 4D). By contrast, both Sn/Al–Na and Zn/Al–Na show higher voltage hysteresis under the same conditions. At 1.0 mA cm^–2^ and 1.0 mAh cm^–2^, the Sn–ZnF_2_/Al–Na presents superior cycling stability over 1500 h (Figure 4E and Table S3). However, Zn/Al–Na exhibits a significant increase in overpotential at 339 h, while Sn/Al–Na delivers increased voltage hysteresis and experiences polarization after 745 h (Figure S37). After cycling, a smooth and dendrite-free surface for the Sn–ZnF_2_/Al–Na electrode is obtained (Figure S38). Generally, high current densities exacerbate Na dendrite growth, leading to a low CE and short cycle life. As shown in Figures S39–S41, the Sn–ZnF_2_/Al–Na electrode displays stable operation at different conditions (2.0 mA cm^–2^/2.0 mAh cm^–2^, 5.0 mA cm^–2^/5.0 mAh cm^–2^, and 8.0 mA cm^–2^/8.0 mAh cm^–2^). Even at 10.0 mA cm^–2^ and 10.0 mAh cm^–2^, this electrode achieves stable cycling over 1000 h (Figures 4F and S42), indicating its excellent durability. The cycling property of Sn–ZnF_2_/Al–Na outperforms that of most reported composite Na electrodes (Table S4).

To evaluate the practical feasibility of Sn–ZnF_2_/Al–Na, anode-less full cells are constructed by pairing an NVOPF cathode with Zn/Al–Na, Sn/Al–Na, or Sn–ZnF_2_/Al–Na anodes (denoted as Zn/Al–Na//NVOPF, Sn/Al–Na//NVOPF, or Sn–ZnF_2_/Al–Na//NVOPF, respectively). The NVOPF cathode material is characterized by XRD, EDX, and FESEM (Figures S43 and S44). Figures S45–S47 show CV curves, cycling performance, and rate capability of Na//NVOPF. In CV profiles, the Sn–ZnF_2_/Al–Na//NVOPF exhibits smaller polarization compared to the Sn/Al–Na//NVOPF and Zn/Al–Na//NVOPF (Figures 5A and S48). Additionally, the Sn–ZnF_2_/Al–Na//NVOPF cell exhibits an enhanced rate capability at various current densities (Figure S49), as further confirmed by its charge–discharge voltage profiles (Figure S50). The EIS result reveals a small charge transfer resistance of Sn–ZnF_2_/Al–Na//NVOPF (Figure S51), which can account for the improvement in rate capability. The Sn–ZnF_2_/Al–Na//NVOPF delivers an excellent cycling property with 97.54% capacity retention after 240 cycles with a negative to positive capacity (N/P) ratio of 1.0 (Figure 5B). By contrast, the full cells with Sn/Al–Na and Zn/Al–Na anodes suffer from failure after 122 and 80 cycles, respectively (Figure S52). The performance enhancement is attributed to high reversibility with a dense morphology (Figure S53). The cycling properties of Sn–ZnF_2_/Al–Na//NVOPF at various N/P ratios are investigated. With an N/P ratio of 0.5, the cell maintains a 97.63% capacity retention after 120 cycles (Figure S54). Stable cycling is consistently achieved across N/P ratios ranging from 0.6 to 0.9 (Figures S55–S58). At a higher N/P ratio of 1.6, this cell demonstrates extended cyclability, retaining 96.64% of its capacity over 400 cycles (Figure S59). In addition, under a high current density of 2.0 C, the cell continues to operate stably, preserving 95.05% capacity retention after 400 cycles (N/P = 1.0, Figures 5C and S60). Compared with previously reported systems, the Sn–ZnF_2_/Al–Na∥NVOPF cell shows superior cycling stability (Table S5).

**Figure 5:**
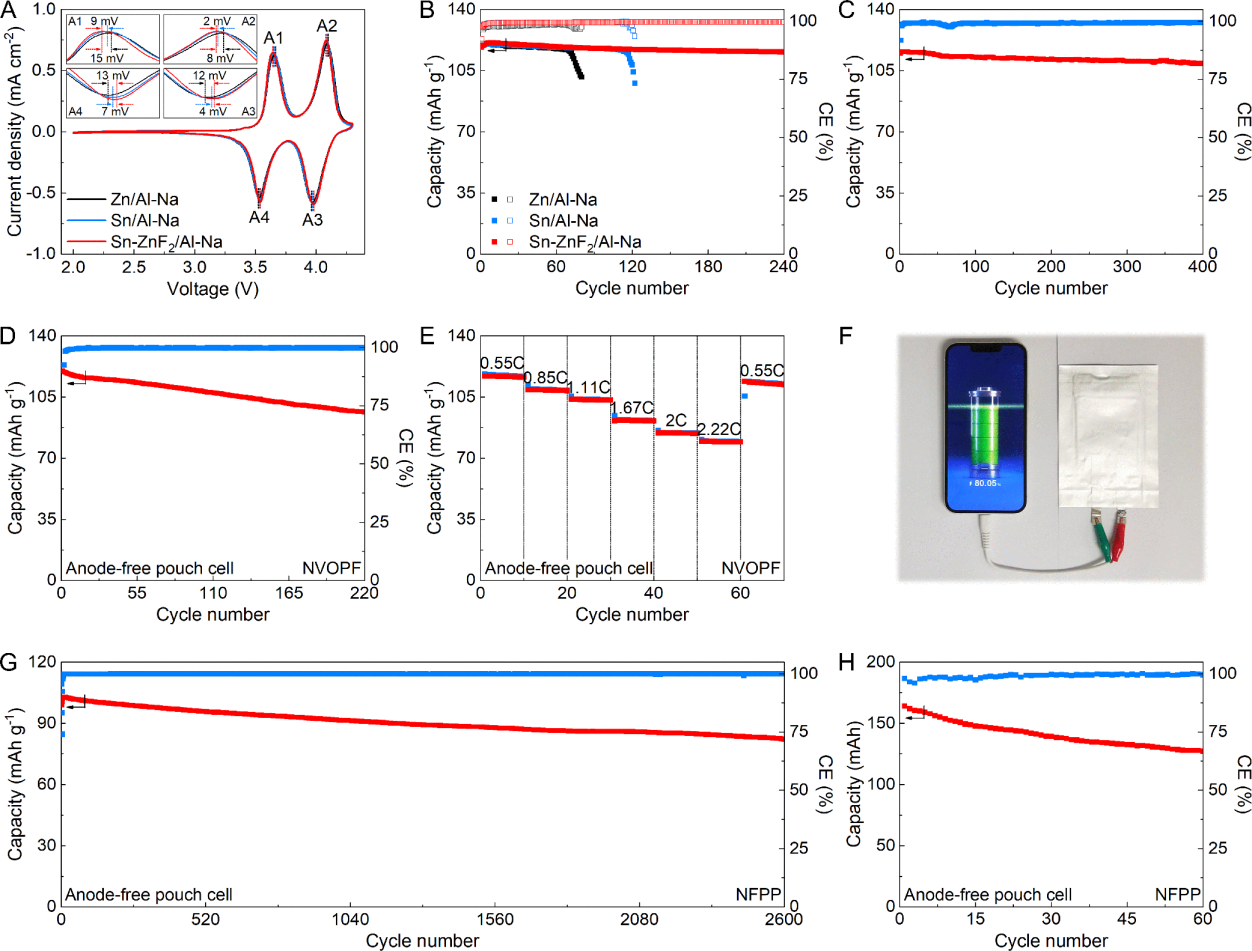
Electrochemical performance of full cells. (A) CV profiles and (B) cycling stability of various full cells. (C) Cycling stability of Sn–ZnF_2_/Al–Na//NVOPF at 2.0C. (D) Cycling stability and (E) rate capability of a single-layer anode-free Sn–ZnF_2_/Al//NVOPF pouch cell. (F) Photographs of a fully charged Sn–ZnF_2_/Al//NVOPF powering a smartphone. (G) Cycling stability of a single-layer anode-free Sn–ZnF_2_/Al//NFPP pouch cell. (H) Cycling stability of a 0.16 Ah-level multilayer anode-free Sn–ZnF_2_/Al//NFPP pouch cell.

The anode-free architecture improves energy density by reducing the weight and volume of cells.^32,44,45^ Therefore, we further assemble anode-free Na pouch cells by pairing NVOPF or NFPP cathodes with an Sn–ZnF_2_/Al anodic current collector (denoted as Sn–ZnF_2_/Al//NVOPF or Sn–ZnF_2_/Al//NFPP, respectively). Figures S61 and S62 show the XRD pattern, EDX result, and FESEM images of NFPP. CV curves, cycling performance, and rate capability of Na//NFPP are explored (Figures S63–S65). In Figures 5D and S66, the anode-free Sn–ZnF_2_/Al//NVOPF pouch cell displays excellent cycling, with 80.37% capacity retention after 220 cycles. Besides, it also delivers an energy density of 442.9 Wh kg^–1^ (calculated based on active materials of both electrodes). The Sn–ZnF_2_/Al//NVOPF pouch cell exhibits a superior rate performance across varying current densities (Figure 5E). With increasing current density from 0.55 C to 2.22 C, stable capacities of 116.7, 108.9, 103.5, 91.5, 84.4, and 79.4 mAh g^–1^ are achieved by the Sn–ZnF_2_/Al//NVOPF pouch cell (Figure S67). This pouch cell enables a stable power supply to electric devices, such as smartphones, LED signs, fans, and LED lights (Figures 5F and S68). Another widely adopted cathode, NFPP, is also paired for assembling an anode-free Sn–ZnF_2_/Al//NFPP pouch cell with 75.75% capacity retention over 1600 cycles at 0.5 C (Figure S69). In addition, this pouch cell exhibits a good rate capability (Figure S70). Even tested at 1.0 C, it manifests stable operation with 80.59% capacity retention over 2600 cycles (Figures 5G and S71). A comprehensive comparison with state-of-the-art works illustrates that the cycling performance of both Sn–ZnF_2_/Al//NVOPF and Sn–ZnF_2_/Al//NFPP pouch cells outperforms that of most reported AFNBs (Table S6). To further validate scalability, a 0.16 Ah-level anode-free Sn–ZnF_2_/Al//NFPP pouch cell is assembled, achieving a stable cycling performance (Figures 5H and S72).

## 3 Conclusion

In summary, a multifunctional interface layer composed of Sn nanodots and ZnF_2_ nanosheets is engineered on an Al current collector to enable energy-dense and durable AFNBs. The abundant sodiophilic Sn and ZnF_2_ sites decrease the Na nucleation energy barrier, further modulating the homogeneous Na nucleation and growth. In addition, the in situ formed NaF-rich SEI promotes uniform Na^+^ ion flux and suppresses side reactions. Consequently, the high reversibility of Na deposition and dissolution is achieved with an average CE of around 99.98% at 8.0 mA cm^–2^ and 1.0 mAh cm^–2^ over 4440 cycles. In addition, the Sn–ZnF_2_/Al–Na electrode can operate for over 1000 h at 10.0 mA cm^–2^ and 10.0 mAh cm^–2^. As a result, an energy-focused Sn–ZnF_2_/Al//NVOPF pouch cell realizes an energy density of 442.9 Wh kg^–1^ (calculated based on active materials of both electrodes) and a capacity retention of 80.37% after 220 cycles. Meanwhile, a lifetime-focused Sn–ZnF_2_/Al//NFPP pouch cell exhibits ultrastable cycling with a capacity retention of 80.59% over 2600 cycles. This work provides a viable pathway for developing high-energy-density batteries via an elaborate interphase design.

## Supplementary Material



## Notes

Experimental and computational details; characterization methods; additional EDX, XRD, XPS, FESEM, TEM, and HAADF-STEM results; electrochemical results; and supplementary notes and tables (PDF)
