# Supplementary material for: Synergistic Regulation of Nucleation and Interfacial Chemistry for Energy-Dense and Durable Anode-Free Na Batteries
Source: J Am Chem Soc. 2026 Apr 8;148(15):16350–8. doi: 10.1021/jacs.6c02019 (PMC13107440; doi:10.1021/jacs.6c02019)
Supplement: Supplementary file 1 [file ja6c02019_si_001.pdf]

Supporting Information for

Synergistic Regulation of Nucleation and Interfacial Chemistry for Energy-Dense and Durable Anode-Free Na Batteries

Yongling An,[†] Zhihao Pei,[†] Jiarui Yang,[†] Deyan Luan,[†] Xiong Wen (David) Lou[†]*

[†]Department of Chemistry, City University of Hong Kong, 83 Tat Chee Avenue, Kowloon, Hong Kong, 999077 China

*Corresponding author. Email: david.lou@cityu.edu.hk (X.W.L.)

MATERIALS AND METHODS

Synthesis of Zn/Al

Zn/Al was fabricated via an electrochemical deposition method. A treated Al foil was served as the working electrode, while a Zn foil was acted as both the counter and reference electrodes. The electrolyte was prepared by dissolving cetyltrimethylammonium bromide (Sigma-Aldrich), ammonium sulfate (Sigma-Aldrich), and zinc sulfate heptahydrate (Sigma-Aldrich) in deionized water. Zn nanosheets were grown on the Al surface by an electrochemical deposition method. After deposition, the obtained Zn/Al substrate was thoroughly washed with deionized water and dried for further use.

Synthesis of Sn-ZnF_2/Al

Sn-ZnF_2/Al was fabricated by a galvanic displacement method. Tin(II) fluoride (Sigma-Aldrich) solution was prepared by dissolving in a mixed solvent composed of dimethyl sulfoxide (Sigma-Aldrich) and deionized water with a concentration of 0.1 M. The as-prepared Zn/Al substrate was then immersed in this solution to allow the spontaneous displacement reaction, resulting in the formation of the Sn-ZnF_2/Al product.

Synthesis of Sn/Al

Sn/Al was fabricated through a similar procedure as Sn-ZnF_2/Al, except that tin(II) chloride dihydrate (Sigma-Aldrich) aqueous solution was used.

Materials characterizations

The crystal phase of the products was determined by X-ray diffraction (XRD) using a Bruker D2 Phaser diffractometer. The elemental chemical states were examined by X-ray photoelectron spectroscopy (XPS, ESCALAB 250Xi). The morphology and structure were investigated by field-

emission scanning electron microscopy (FESEM, JSM-7800F), transmission electron microscopy (TEM, JEOL JEM-2100), and focused ion beam transmission electron microscopy (FIB-TEM, FEI Scios 2 HiVac, Talos F200X G2). Elemental distribution was visualized via TEM equipped with energy-dispersive X-ray (EDX) spectroscopy. The chemical composition was quantitatively assessed using EDX spectroscopy equipped with both the FESEM and TEM systems.

Electrochemical measurements

Electrochemical tests were performed on a NEWARE testing system. Electrochemical impedance spectroscopy (EIS) was acquired on a CHI 660E electrochemical workstation in the frequency range of 100 kHz to 10 MHz, and cyclic voltammetry (CV) was characterized on the same equipment. To explore the Coulombic efficiency (CE) of Na plating and stripping, Na foil was used as both the counter electrode and reference electrode, while Zn/Al, Sn/Al, and Sn-ZnF_2/Al were used as the working electrodes, respectively. The diameter of the working electrode was 10 mm. 1 M sodium hexafluorophosphate ($NaPF_6$) in diglyme was used as an electrolyte. The electrolyte volume was approximately 80 μL per cell. The cell was first cycled between 0.01 V and 1.0 V at 0.05 mA for aging and activation. It was subsequently discharged under varying current densities and time, and charged to 1.0 V. To evaluate the cycling performance, Na was pre-plated on Zn/Al, Sn/Al, and Sn-ZnF_2/Al with different areal capacities to fabricate the Zn/Al-Na, Sn/Al-Na, and Sn-ZnF_2/Al-Na electrodes, respectively. The depth of discharge was 50%. In anode-less and anode-free cells, commercial $Na_3V_2O_2(PO_4)_2F$ (NVOPF) and $Na_4Fe_3(PO_4)_2P_2O_7$ (NFPP) were used as the cathodes. The NVOPF and NFPP powders were purchased from Canrd Technology Co. Ltd. The NVOPF cathode was fabricated by blending NVOPF powder, Ketjen black, and polyvinylidene fluoride in a weight ratio of 7:2:1 using N-methyl-2-pyrrolidone as the solvent to form a homogeneous slurry. This

slurry was coated onto an Al foil and subsequently dried overnight at 150 °C under vacuum. The dried electrode was then cut into a plate with a diameter of 10 mm. The mass loading of active materials was 1.5-2.2 mg cm^{-2}. The NFPP cathode was fabricated through a similar procedure as NVOPF, except that NFPP powder was used with a weight ratio of 8:1:1. The anode-less full cells were assembled using NVOPF as the cathode, Zn/Al-Na, Sn/Al-Na, and Sn-ZnF_2/Al-Na as the anodes, respectively. These anodes were fabricated by pre-plating Na on Zn/Al, Sn/Al, and Sn-ZnF_2/Al hosts with different areal capacities. The diameters of the cathode and anode were 10 mm. 1 M $NaPF_6$ in diglyme was used as an electrolyte. The cells were cycled between 2.0 V and 4.3 V at different current densities. The anode-free pouch cells were assembled in a glove box using NVOPF or NFPP as the cathode, Sn-ZnF_2/Al as the anodic current collector. 1 M $NaPF_6$ in diglyme was used as the electrolyte. An aluminum-plastic film was used as the soft case for the pouch cell. The size of the pouch cell was 8 × 12 cm. The cells were cycled under compression from a pressure fixture and underwent no additional formation processes. Charge and discharge tests were performed using a NEWARE battery testing system at 25 °C.

Computational details

The Vienna ab initio simulation package was utilized for density functional theory calculations. The interactions between ions and electrons were modeled using the projector augmented wave method. The generalized gradient approximation based on the Perdew-Burke-Ernzerhof scheme was employed for the description of electron exchange and correlation interactions. Plane-wave basis with wave function cut-off energy of 450 eV was utilized. The atomic relaxation was terminated once the total energy tolerance converged to 10^{-5} eV and the changes of the force on atoms were less than 0.02 eV $Å^{-1}$. Additionally, all structures were optimized using a (2×2×1) Monkhorst-Pack K-point grid

considering the symmetry of supercell, accuracy of calculation, and cost of used time. A vacuum space of 15 Å was placed along the Z axis to avoid the interaction among the slab.

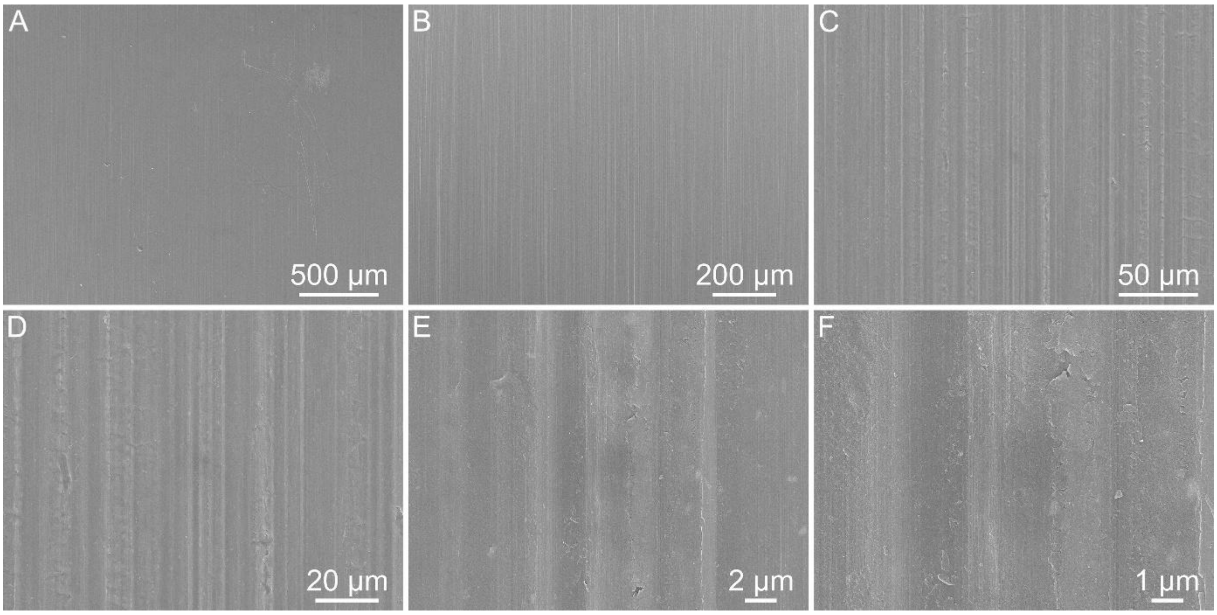

Figure S1. FESEM images of Al foil.

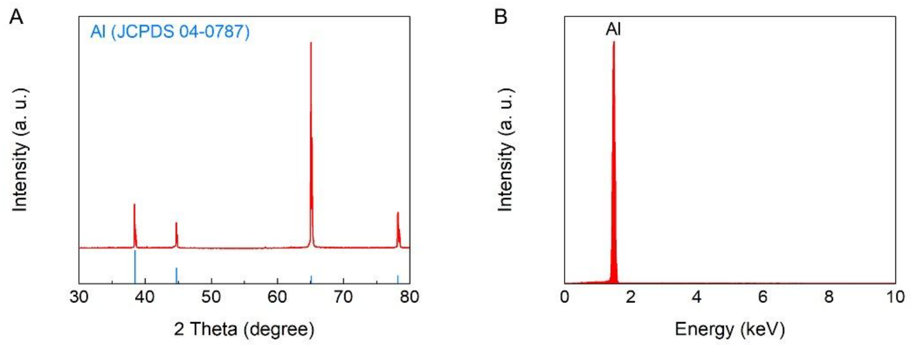

Figure S2. (A) XRD pattern and (B) EDX spectrum of Al foil.

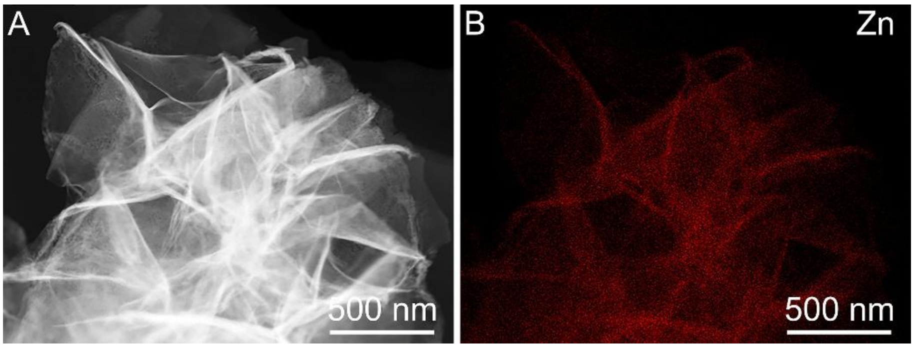

Figure S3. (A) HAADF-STEM and (B) elemental mapping images of Zn/Al.

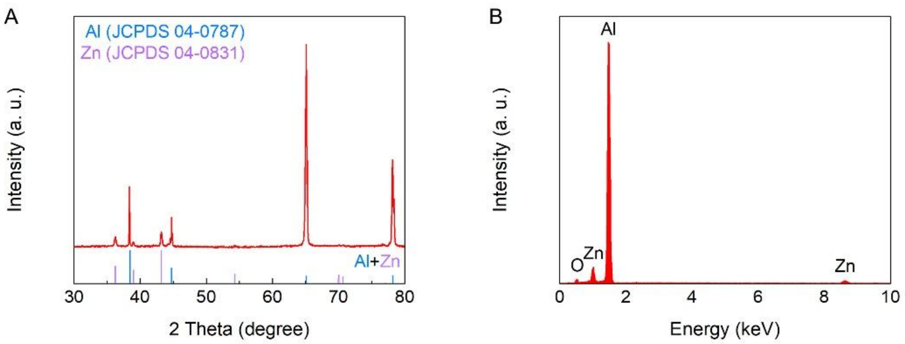

Figure S4. (A) XRD pattern and (B) EDX spectrum of Zn/Al.

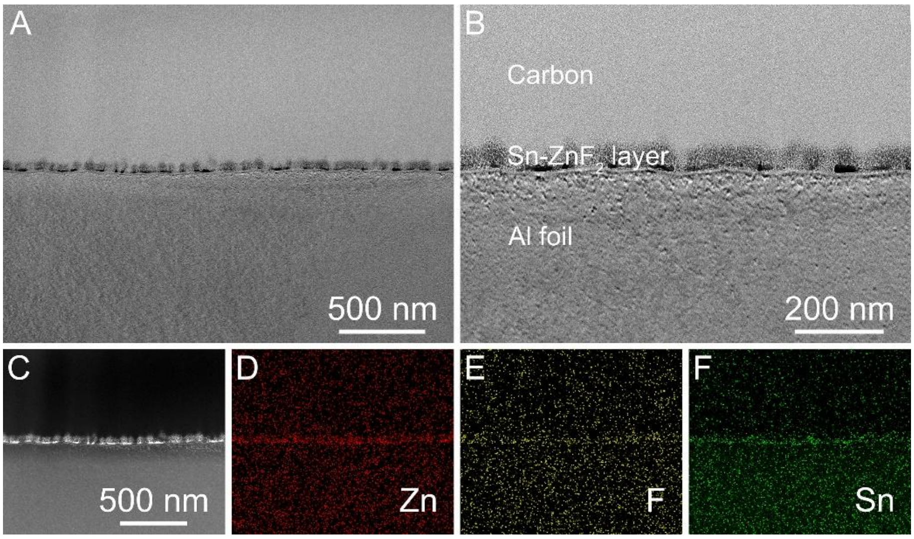

Figure S5. (A, B) FIB-TEM, (C) HAADF-STEM, and (D-F) elemental mapping images of Sn-ZnF_2/Al.

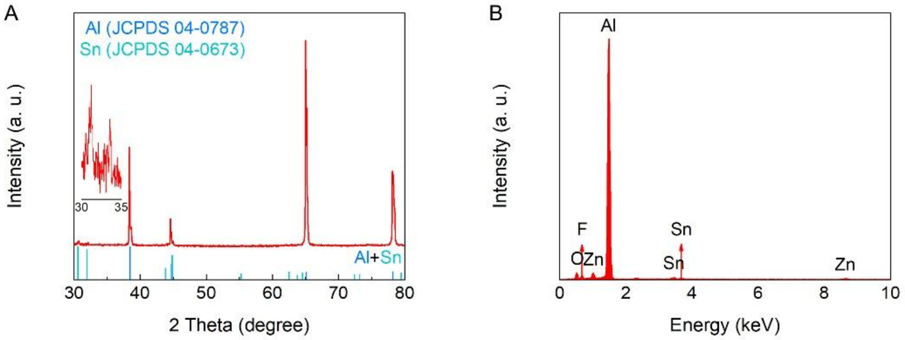

Figure S6. (A) XRD pattern and (B) EDX spectrum of Sn-ZnF_2/Al.

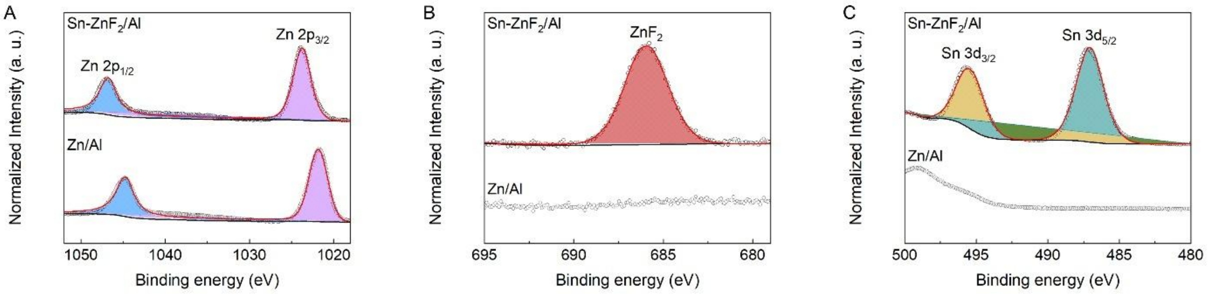

Figure S7. High-resolution (A) Zn 2p, (B) F 1s, and (C) Sn 3d XPS spectra of Zn/Al and Sn-ZnF_2/Al.

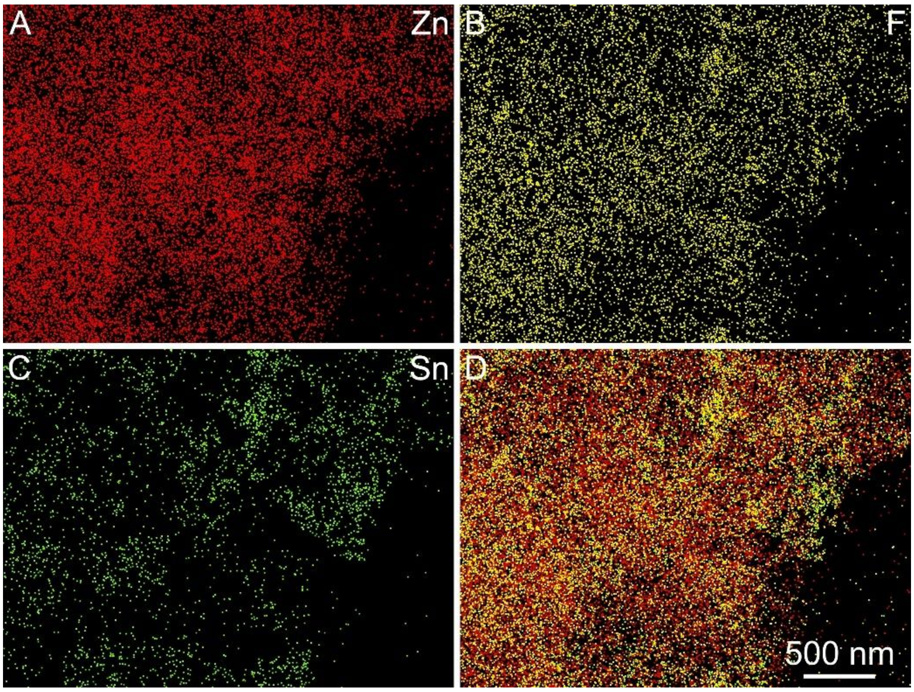

Figure S8. Elemental mapping images of Sn-ZnF_2/Al.

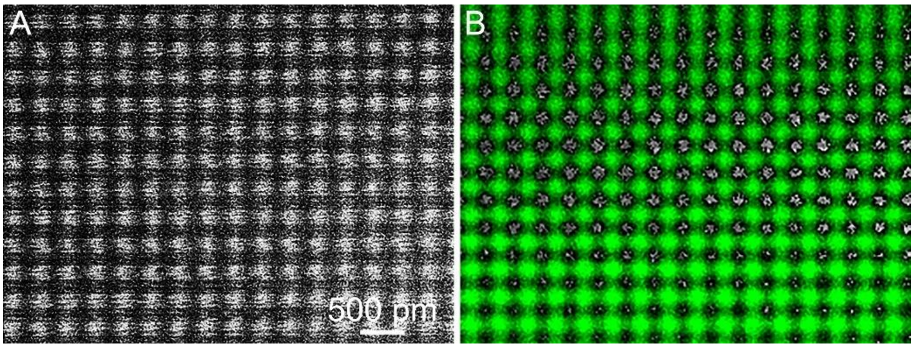

Figure S9. (A) HAADF-STEM and (B) elemental mapping images of Sn-ZnF_2/Al.

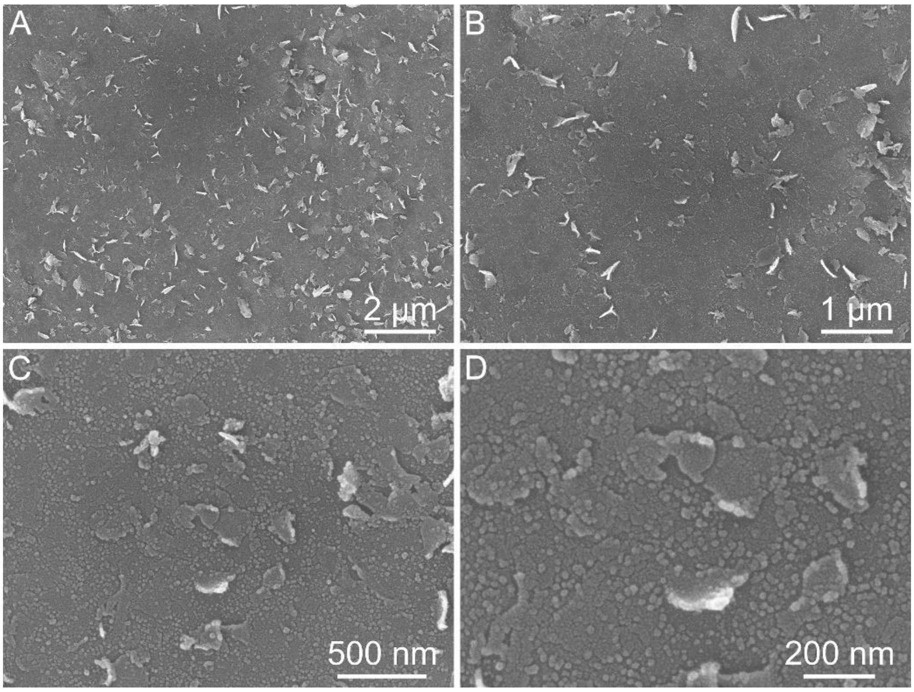

Figure S10. FESEM images of Sn-ZnF_2/Al synthesized in a solvent medium with 0 % H_2O.

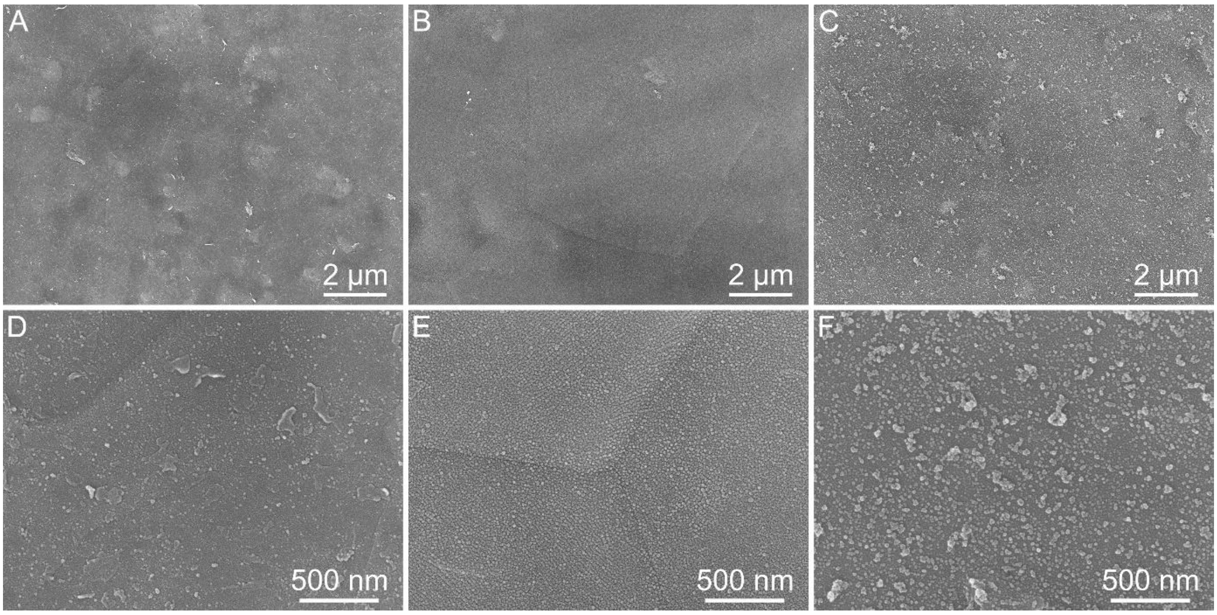

Figure S11. FESEM images of Sn-ZnF_2/Al synthesized with different H_2O content in a solvent medium: (A, D) 5.0 %, (B, E) 10.0 %, and (C, F) 15.0 %.

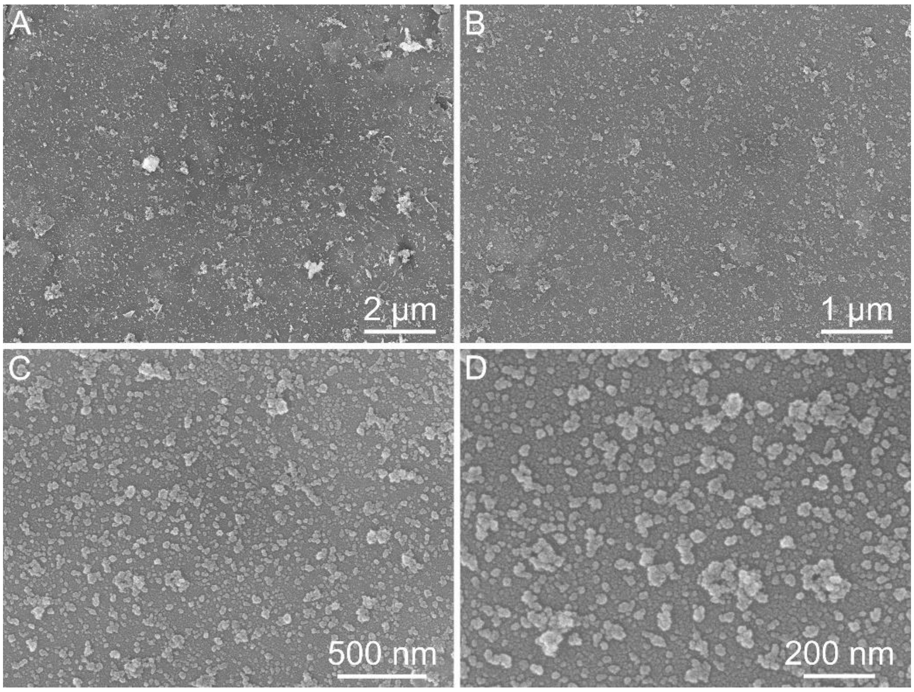

Figure S12. FESEM images of Sn-ZnF_2/Al synthesized in a solvent medium with 20.0 % H_2O.

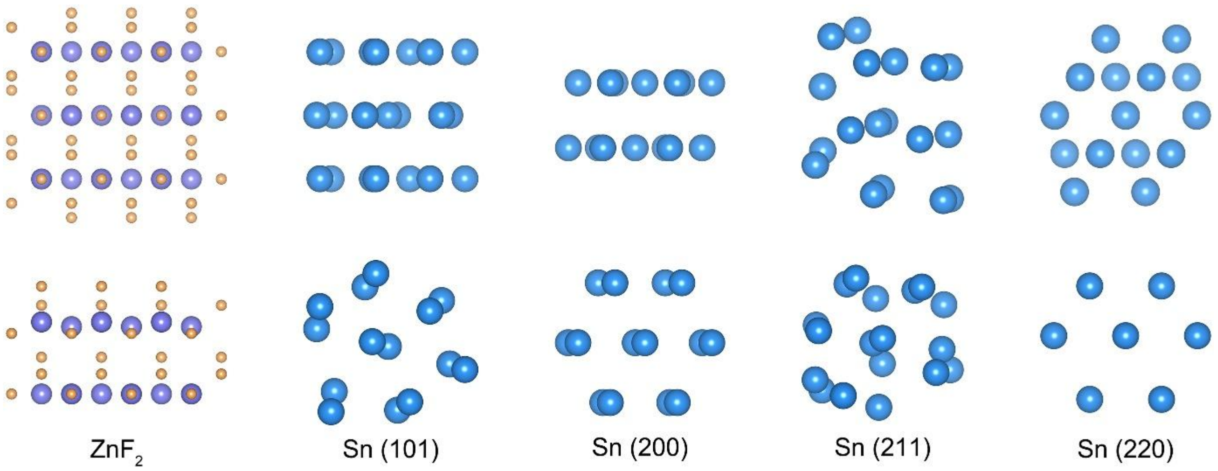

Figure S13. Models of ZnF_2 and Sn.

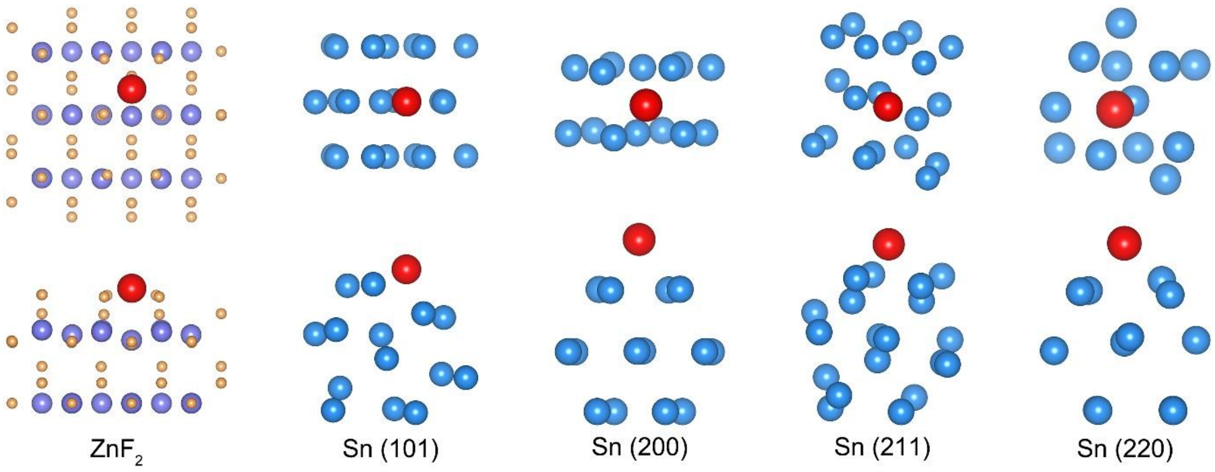

Figure S14. Models of ZnF_2 and Sn after Na atom adsorption.

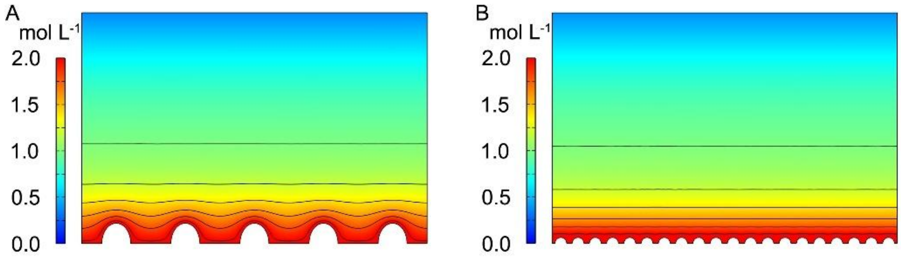

Figure S15. Simulated distributions of electrolyte concentration on (A) Al and (B) Sn-ZnF_2/Al.

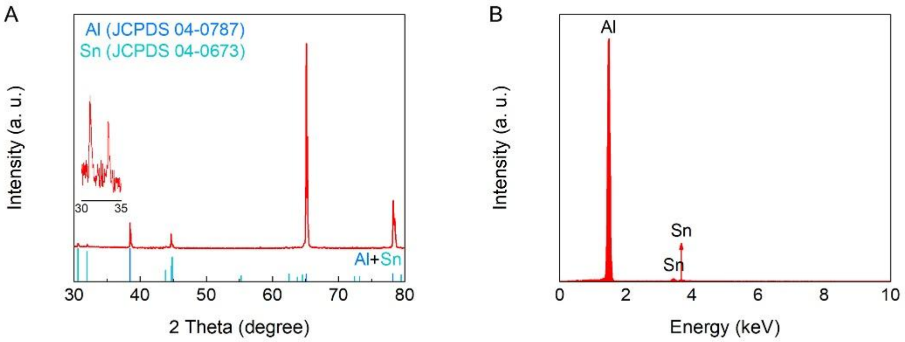

Figure S16. (A) XRD pattern and (B) EDX spectrum of Sn/Al.

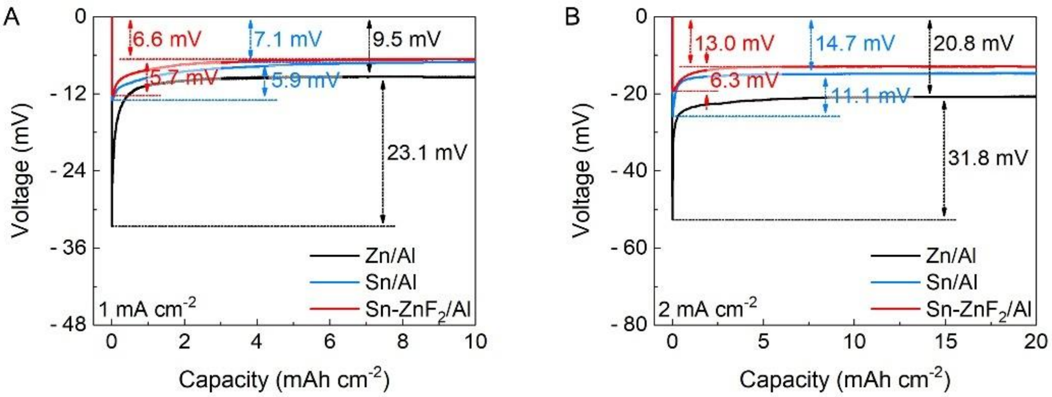

Figure S17. Voltage-capacity curves of Na plating on different hosts tested at different current densities. (A) 1.0 mA cm^{-2} and (B) 2.0 mA cm^{-2}.

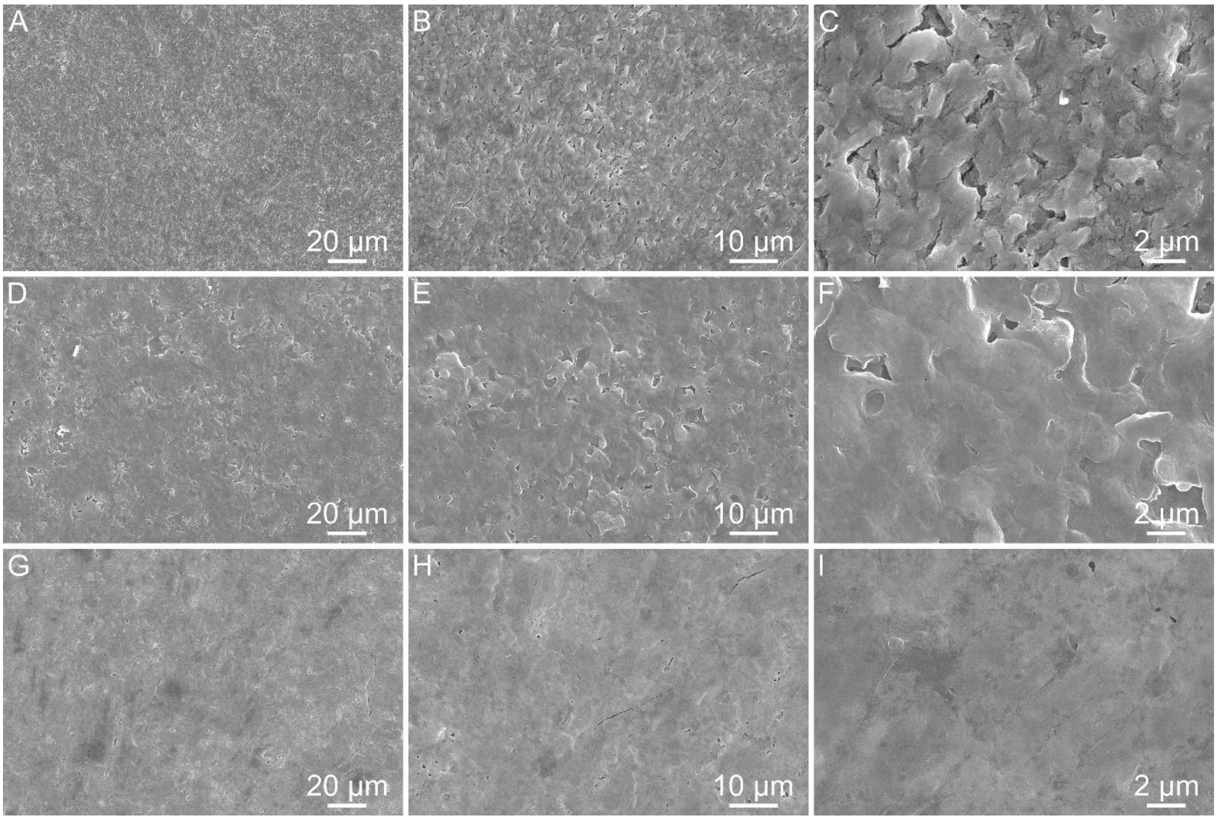

Figure S18. FESEM images of different hosts after the Na deposition at a current density of 1.0 mA cm^{-2} with an areal capacity of 10.0 mAh cm^{-2}. (A-C) Zn/Al, (D-F) Sn/Al, and (G-I) Sn-ZnF_2/Al.

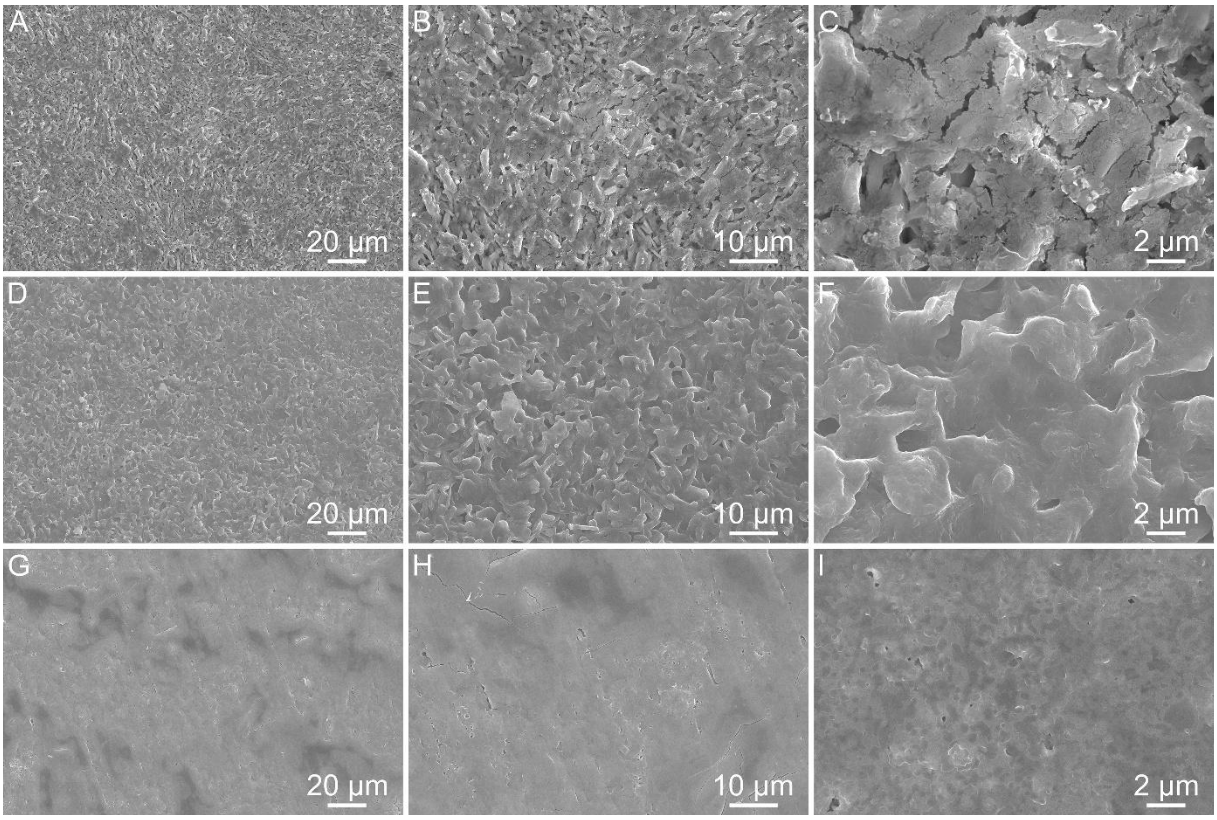

Figure S19. FESEM images of different hosts after the Na deposition at a current density of 1.0 mA cm^{-2} with an areal capacity of 20.0 mAh cm^{-2}. (A-C) Zn/Al, (D-F) Sn/Al, and (G-I) Sn-ZnF_2/Al.

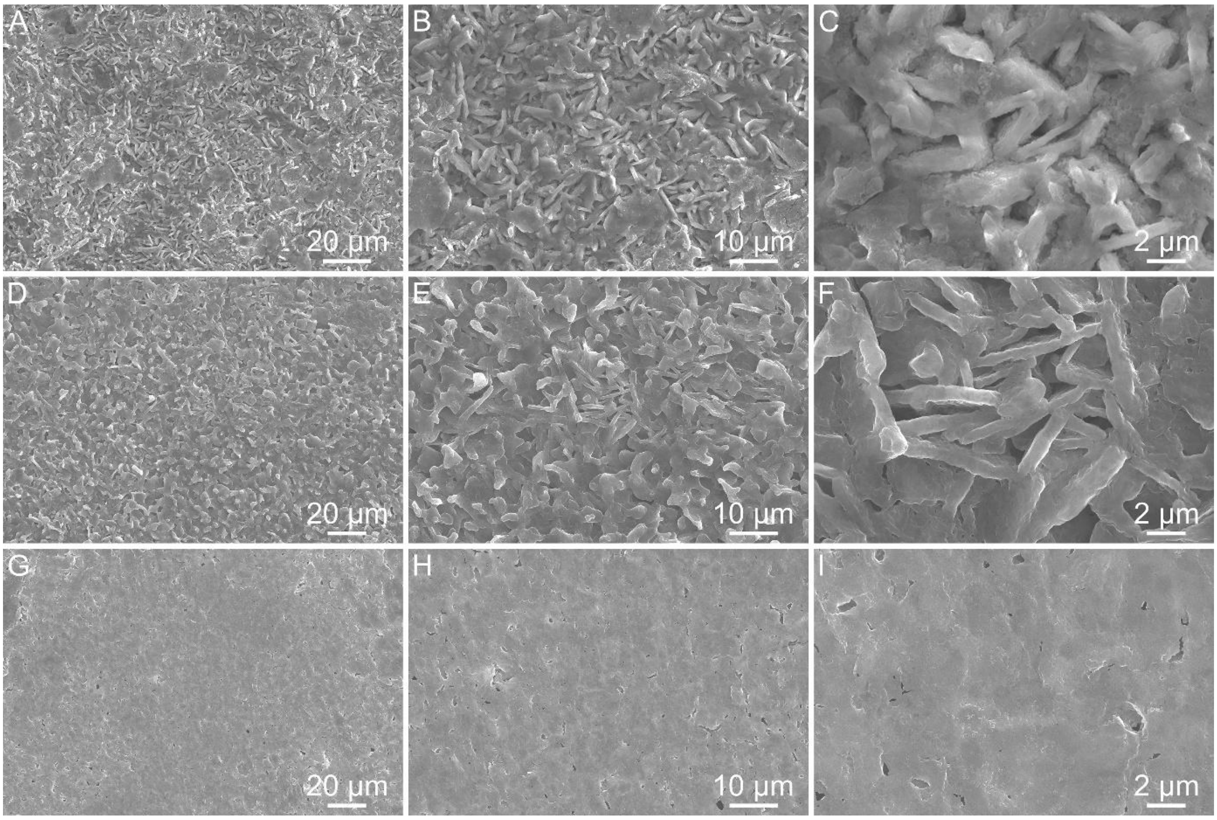

Figure S20. FESEM images of different hosts after the Na deposition at a current density of 1.0 mA cm^{-2} with an areal capacity of 30.0 mAh cm^{-2}. (A-C) Zn/Al, (D-F) Sn/Al, and (G-I) Sn-ZnF_2/Al.

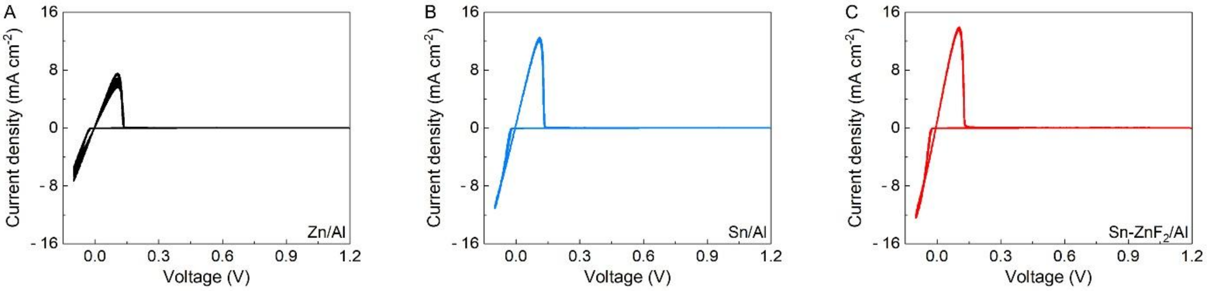

Figure S21. CV curves of different hosts tested at a scan rate of 0.5 mV s^{-1}. (A) Zn/Al, (B) Sn/Al, and (C) Sn-ZnF_2/Al.

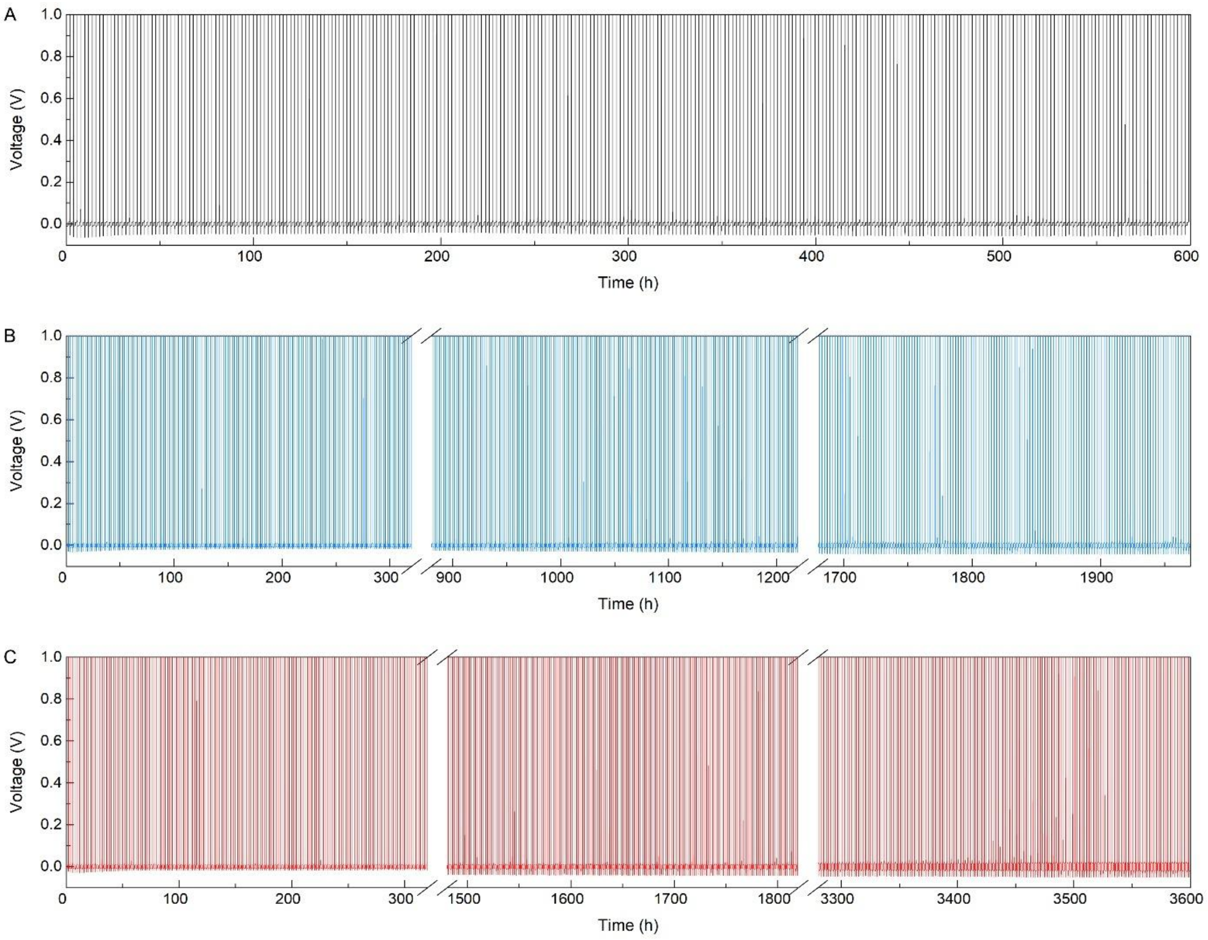

Figure S22. Voltage-time curves of different hosts tested at 1.0 mA cm^{-2} and 1.0 mAh cm^{-2}. (A) Zn/Al, (B) Sn/Al, and (C) Sn-ZnF_2/Al.

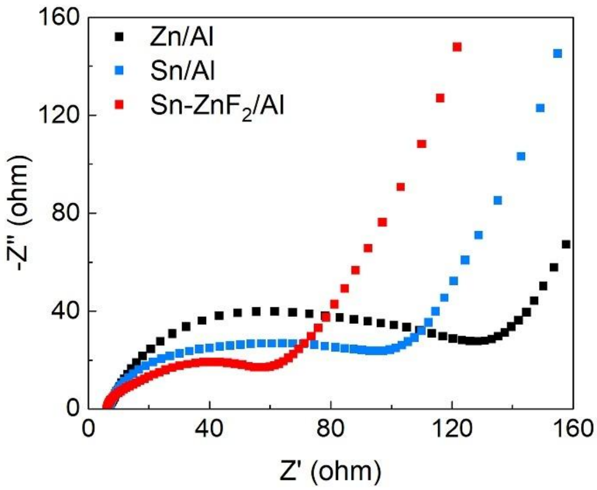

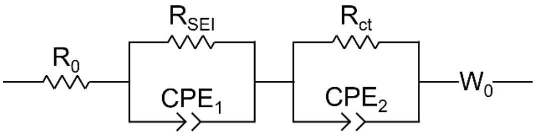

Figure S23. Nyquist plots and equivalent circuit diagram of different hosts after cycling.

R_0 represents the internal resistance. R_{SEI} represents the interfacial resistance across SEI. R_{ct} represents the charge transfer resistance. CPE_1 and CPE_2 represent the constant phase element.

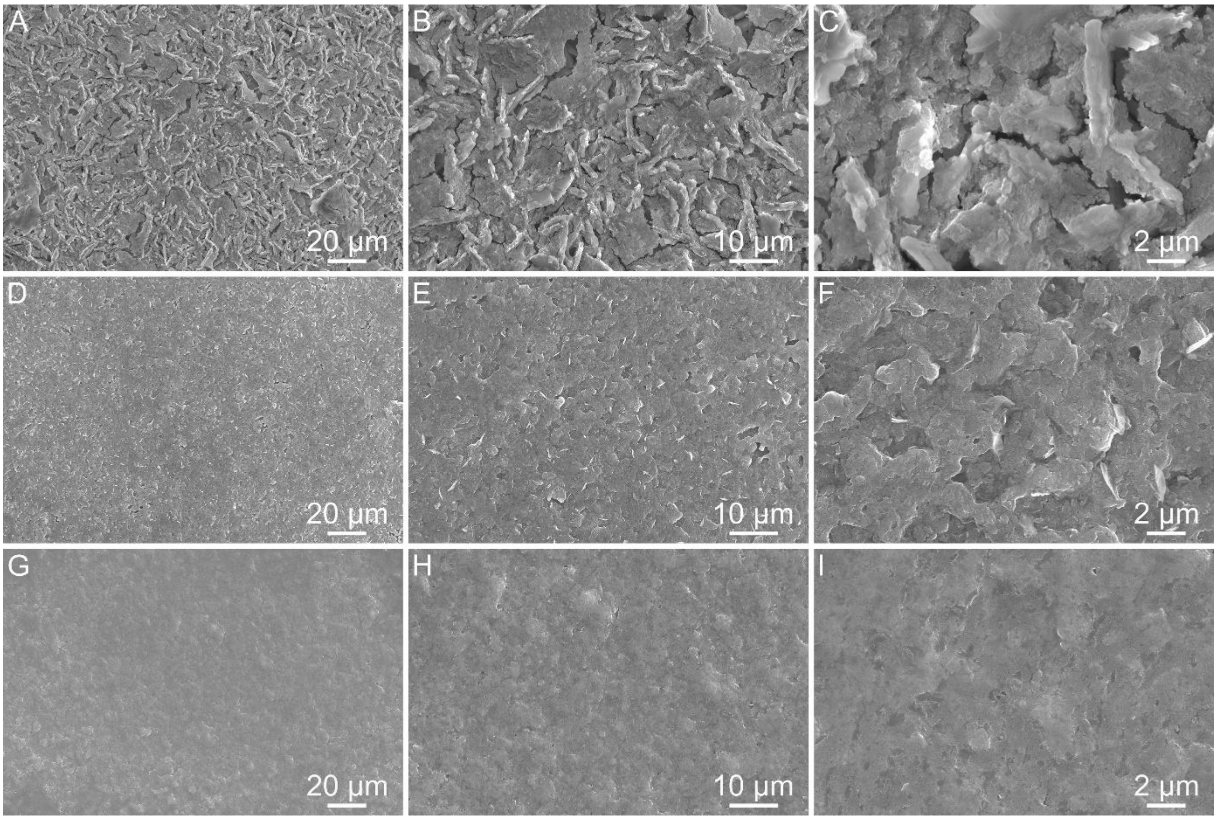

Figure S24. FESEM images of different hosts after cycling. (A-C) Zn/Al, (D-F) Sn/Al, and (G-I) Sn-ZnF_2/Al.

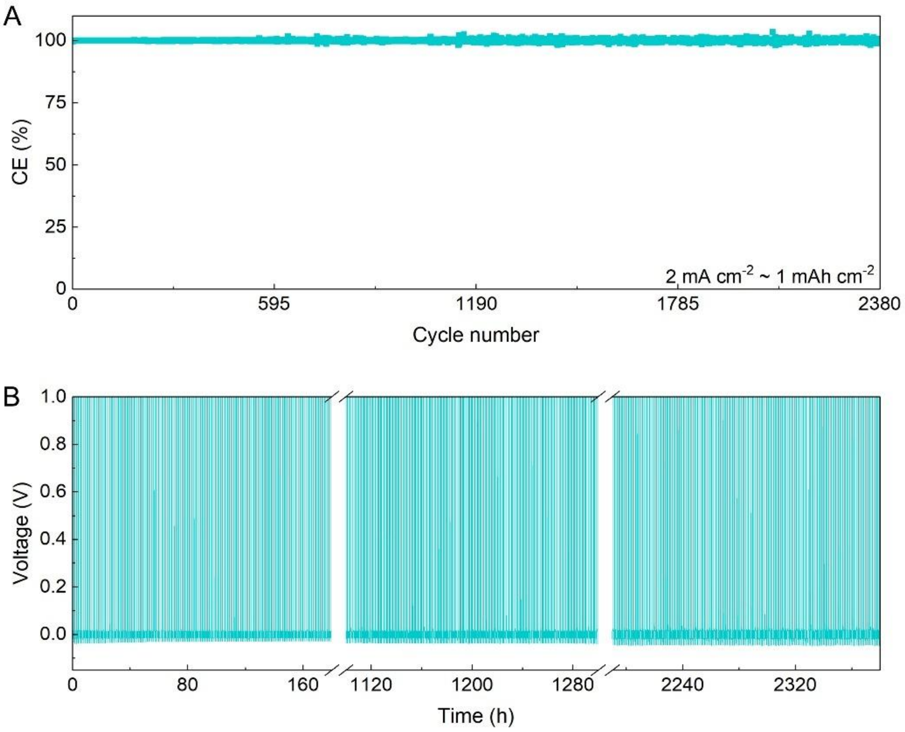

Figure S25. (A) CE plot and (B) voltage-time profile of Sn-ZnF_2/Al host tested at 2.0 mA cm^{-2} and 1.0 mAh cm^{-2}.

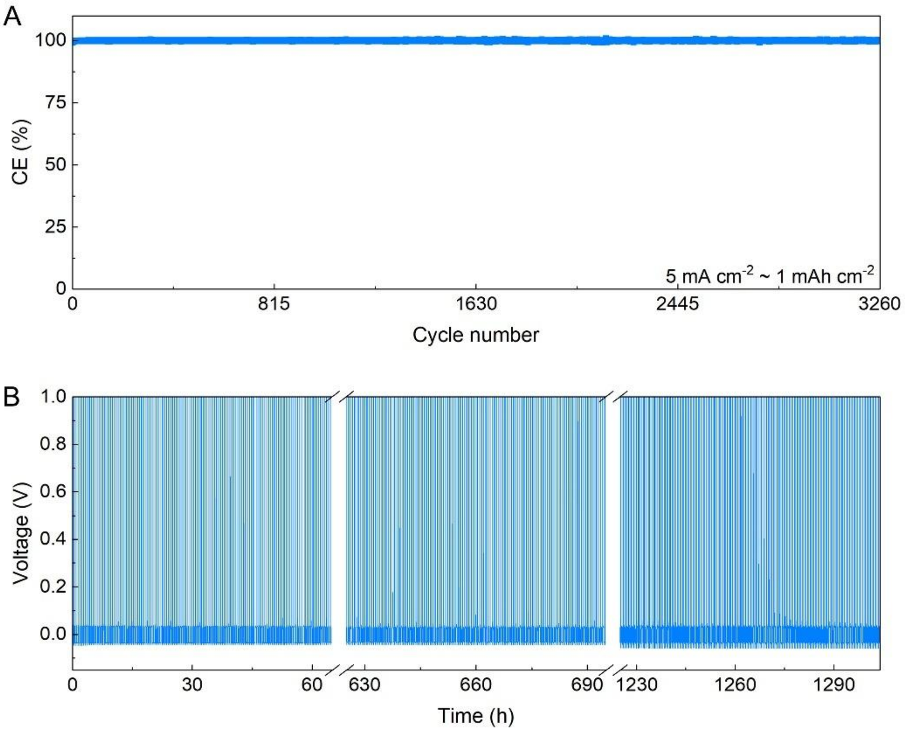

Figure S26. (A) CE plot and (B) voltage-time profile of Sn-ZnF_2/Al host tested at 5.0 mA cm^{-2} and 1.0 mAh cm^{-2}.

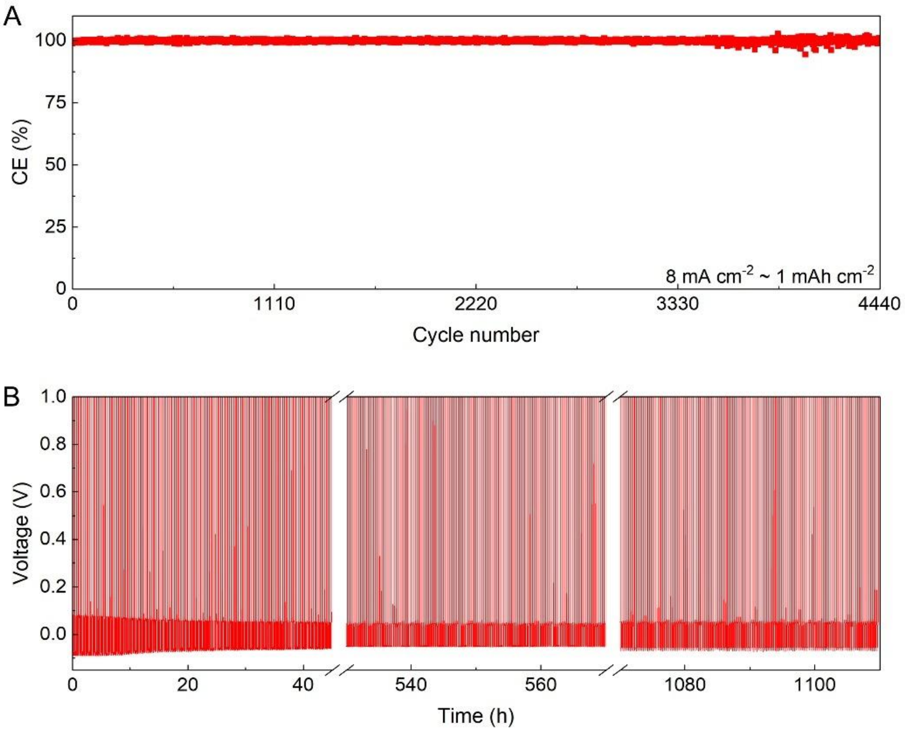

Figure S27. (A) CE plot and (B) voltage-time profile of Sn-ZnF_2/Al host tested at 8.0 mA cm^{-2} and 1.0 mAh cm^{-2}.

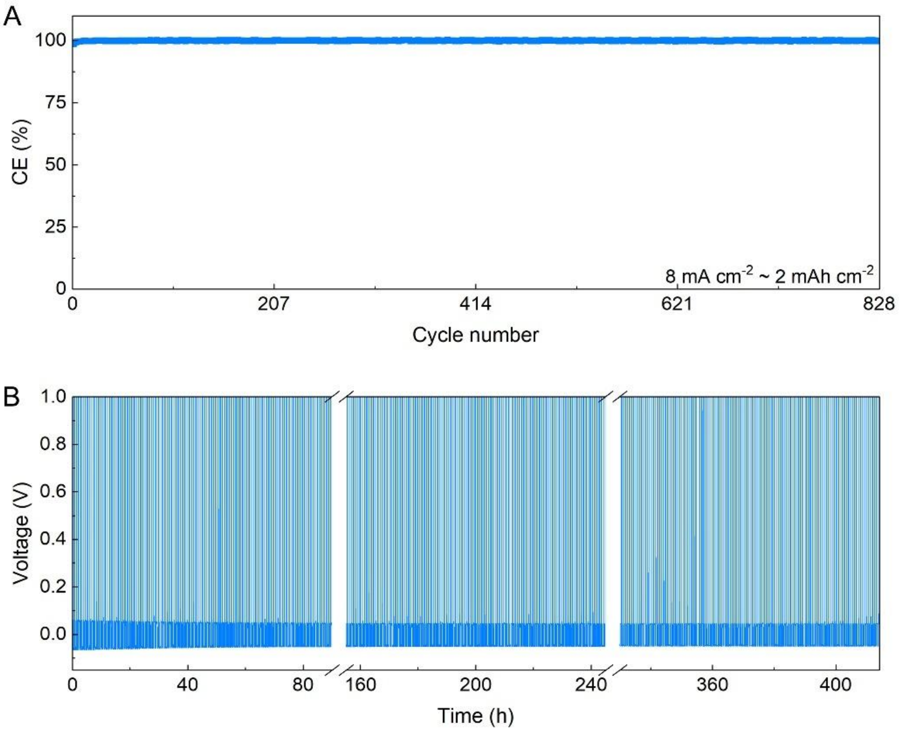

Figure S28. (A) CE plot and (B) voltage-time profile of Sn-ZnF_2/Al host tested at 8.0 mA cm^{-2} and 2.0 mAh cm^{-2}.

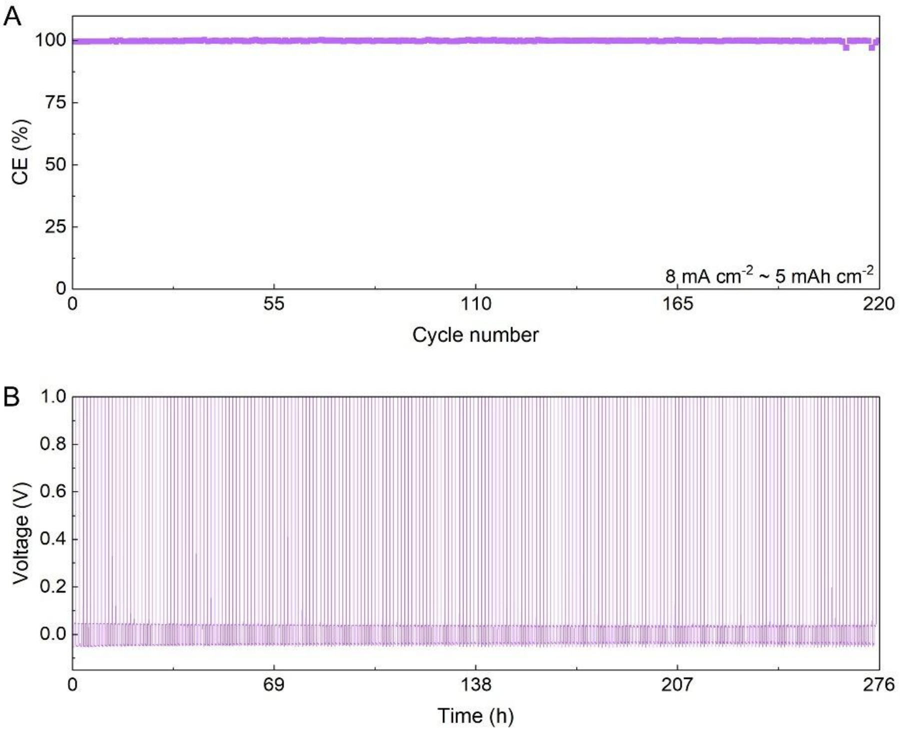

Figure S29. (A) CE plot and (B) voltage-time profile of Sn-ZnF_2/Al host tested at 8.0 mA cm^{-2} and 5.0 mAh cm^{-2}.

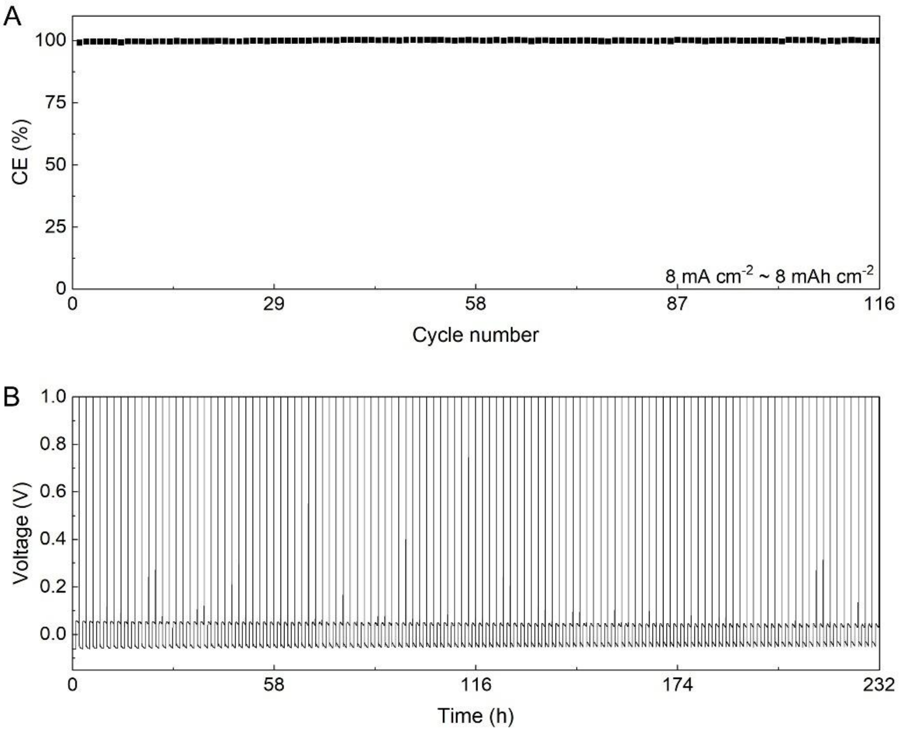

Figure S30. (A) CE plot and (B) voltage-time profile of Sn-ZnF_2/Al host tested at 8.0 mA cm^{-2} and 8.0 mAh cm^{-2}.

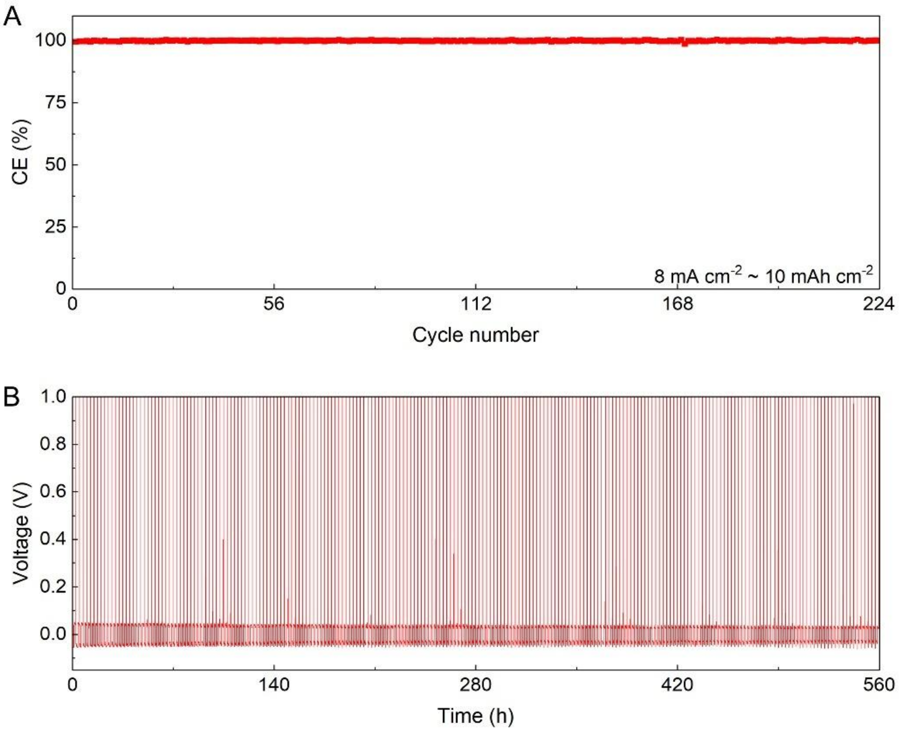

Figure S31. (A) CE plot and (B) voltage-time profile of Sn-ZnF_2/Al host tested at 8.0 mA cm^{-2} and 10.0 mAh cm^{-2}.

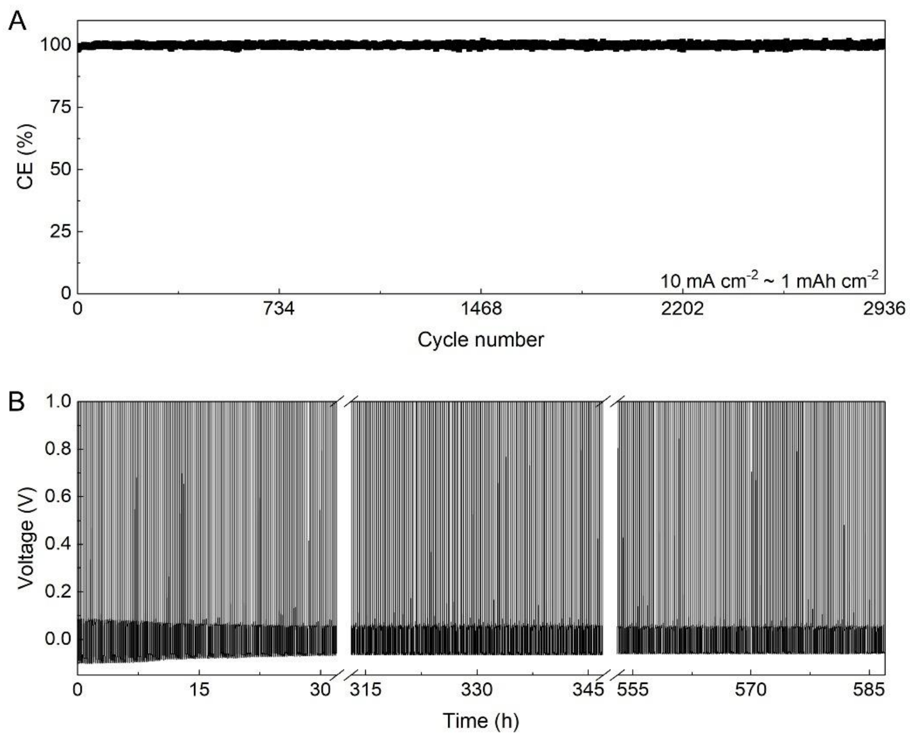

Figure S32. (A) CE plot and (B) voltage-time profile of Sn-ZnF_2/Al host tested at 10.0 mA cm^{-2} and 1.0 mAh cm^{-2}.

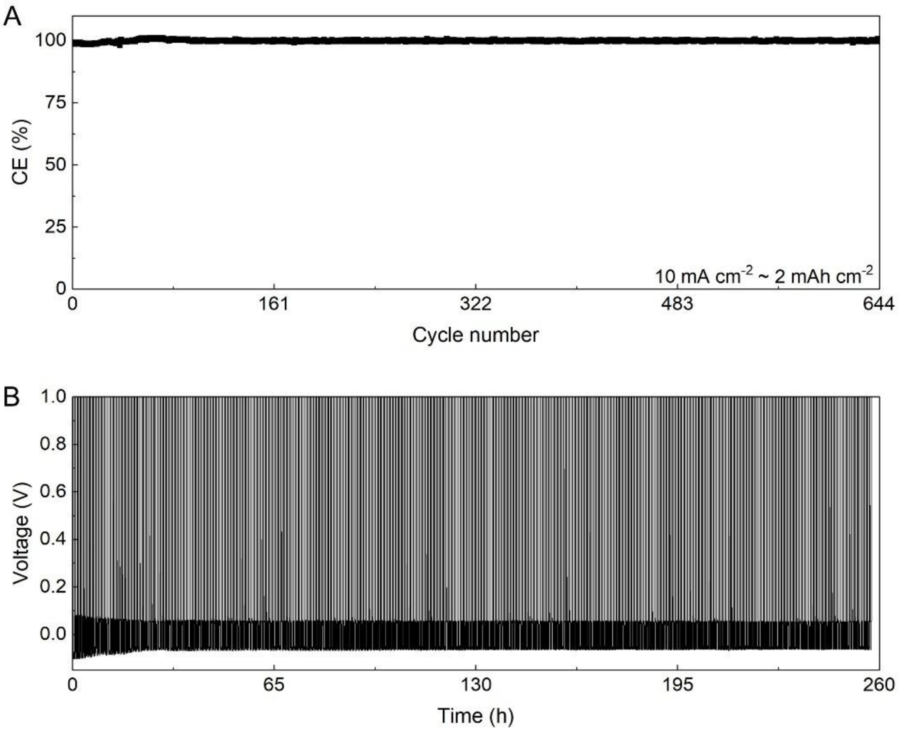

Figure S33. (A) CE plot and (B) voltage-time profile of Sn-ZnF_2/Al host tested at 10.0 mA cm^{-2} and 2.0 mAh cm^{-2}.

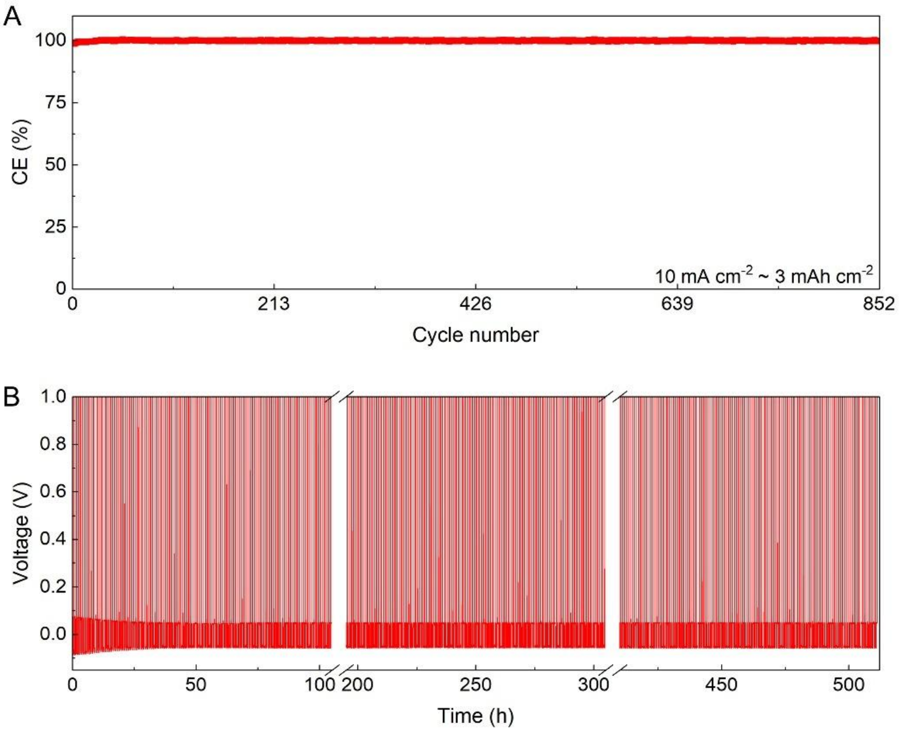

Figure S34. (A) CE plot and (B) voltage-time profile of Sn-ZnF_2/Al host tested at 10.0 mA cm^{-2} and 3.0 mAh cm^{-2}.

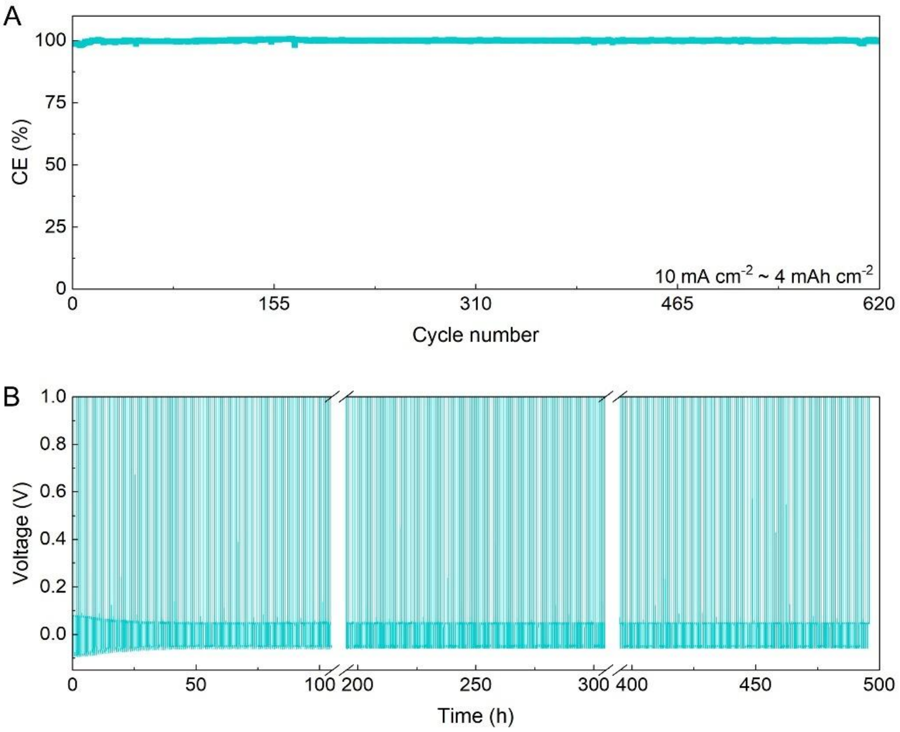

Figure S35. (A) CE plot and (B) voltage-time profile of Sn-ZnF_2/Al host tested at 10.0 mA cm^{-2} and 4.0 mAh cm^{-2}.

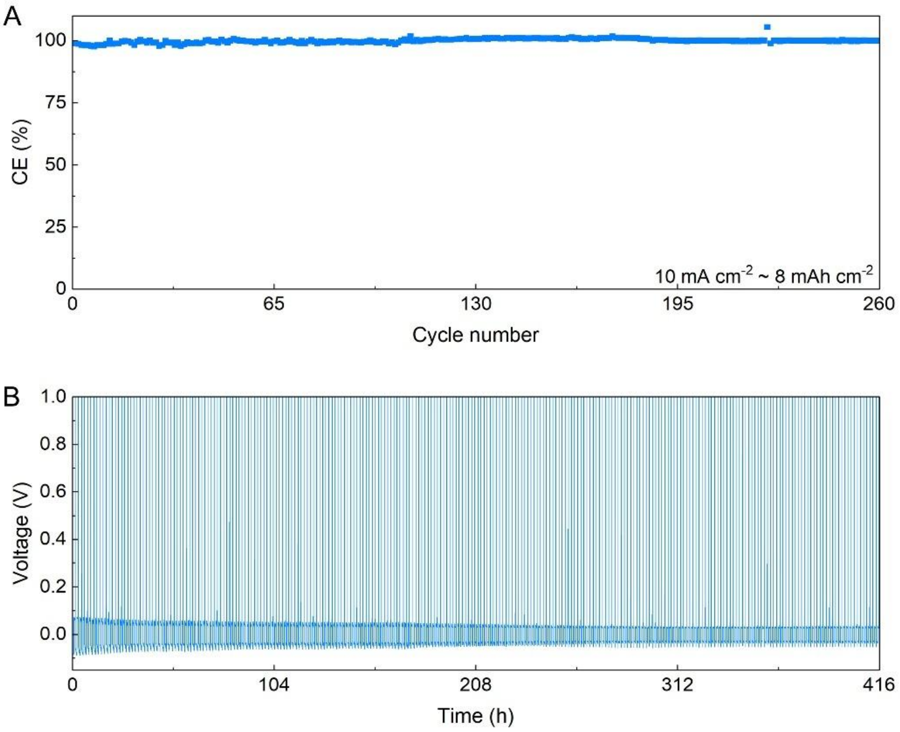

Figure S36. (A) CE plot and (B) voltage-time profile of Sn-ZnF_2/Al host tested at 10.0 mA cm^{-2} and 8.0 mAh cm^{-2}.

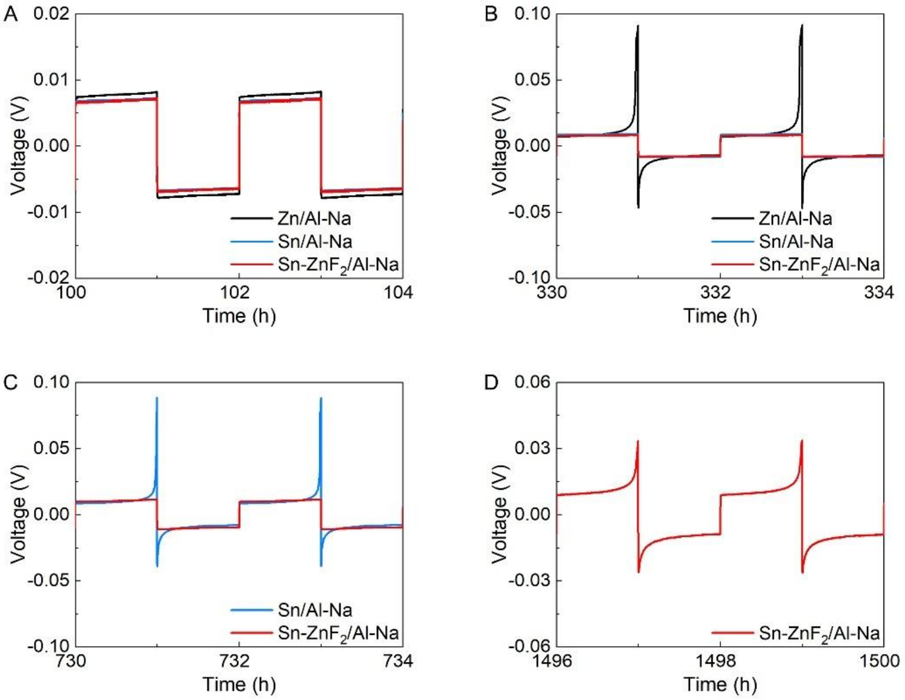

Figure S37. Voltage-time curves of different electrodes tested at 1.0 mA cm^{-2} and 1.0 mAh cm^{-2}.

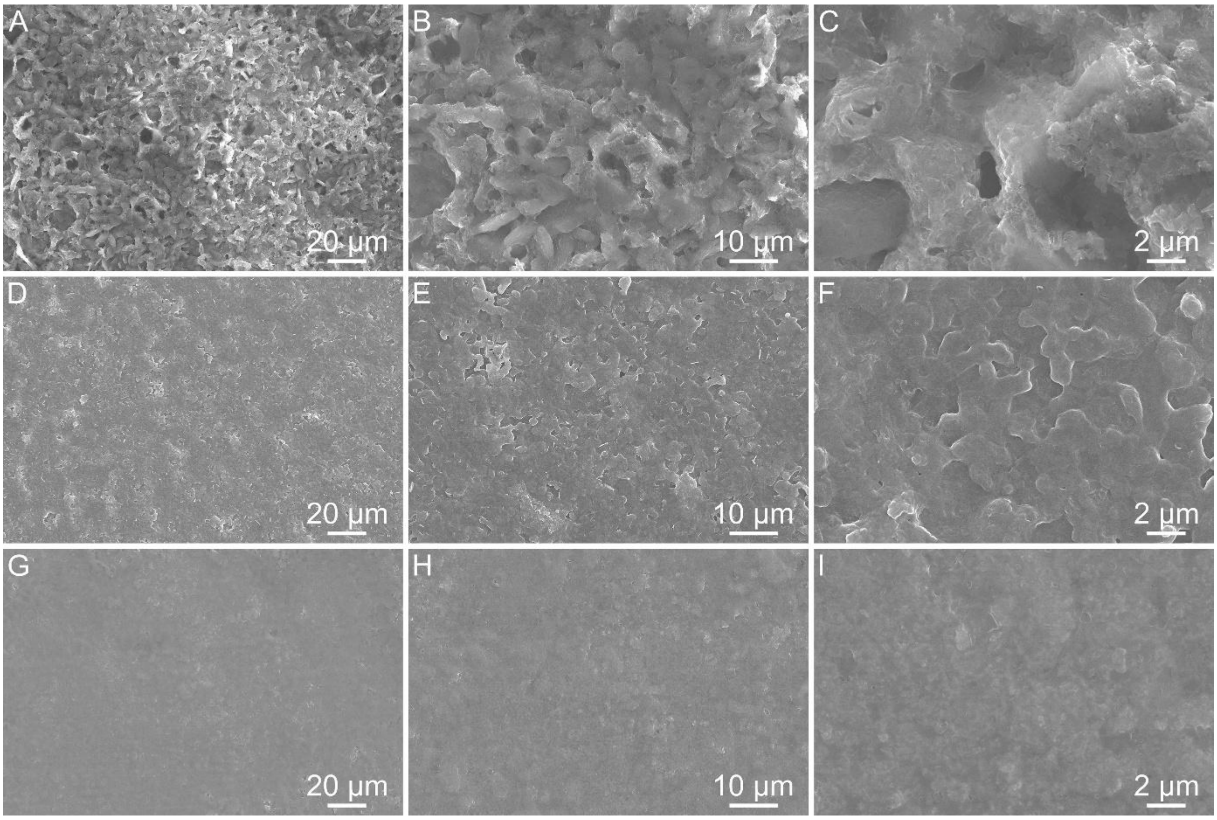

Figure S38. FESEM images of different electrodes after cycling. (A-C) Zn/Al-Na, (D-F) Sn/Al-Na, and (G-I) Sn-ZnF_2/Al-Na.

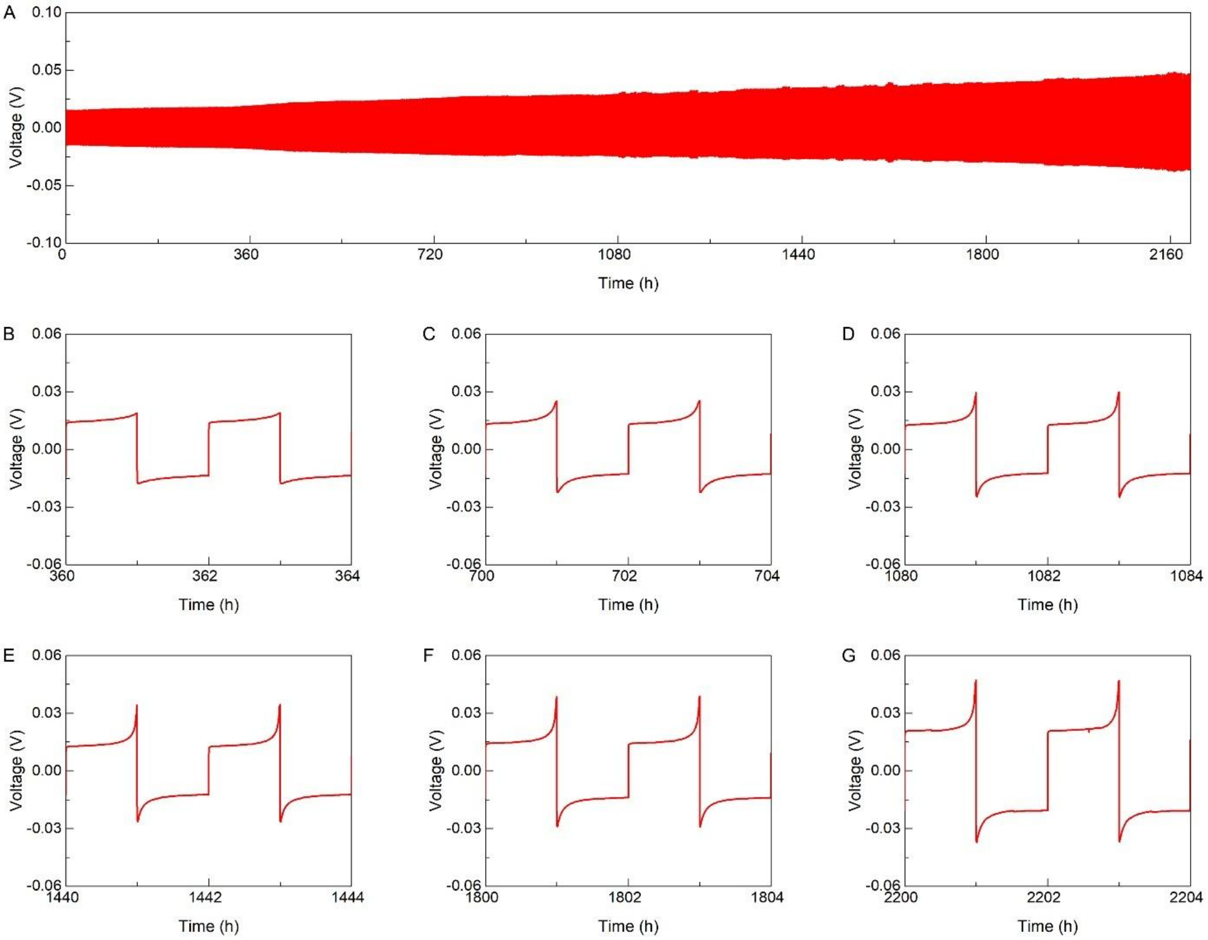

Figure S39. (A) Cycling performance and (B-G) voltage-time curves of Sn-ZnF_2/Al-Na electrode tested at 2.0 mA cm^{-2} and 2.0 mAh cm^{-2}.

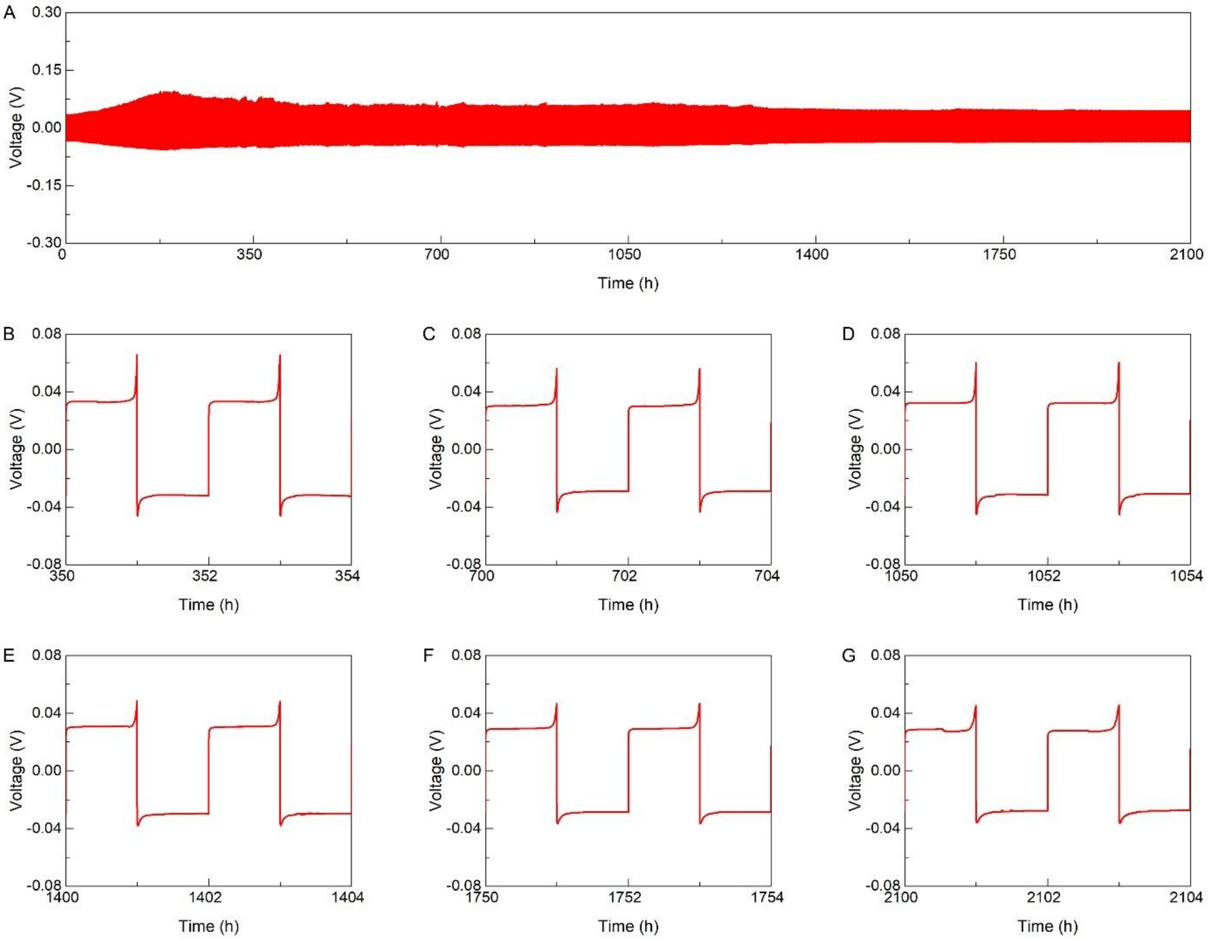

Figure S40. (A) Cycling performance and (B-G) voltage-time curves of Sn-ZnF_2/Al-Na electrode tested at 5.0 mA cm^{-2} and 5.0 mAh cm^{-2}.

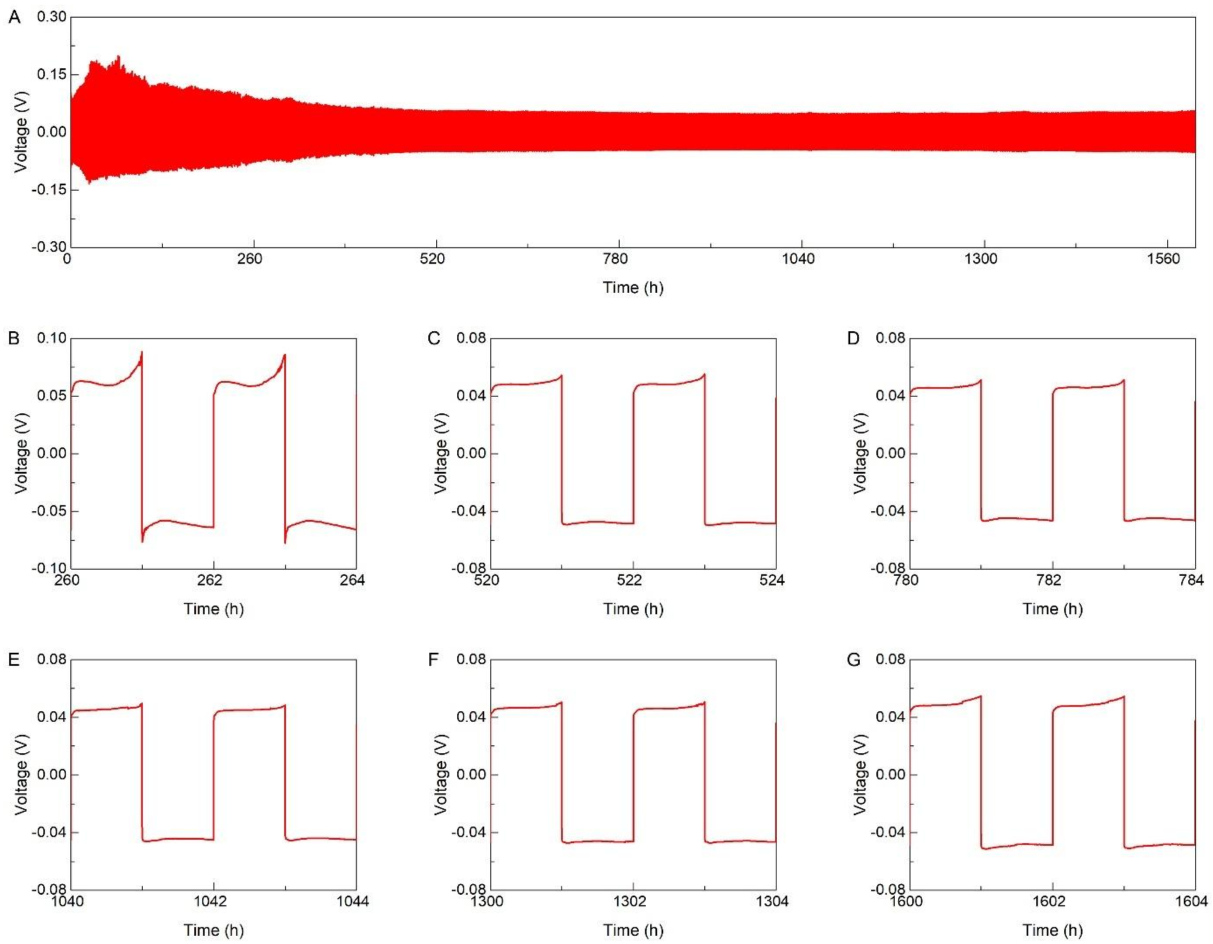

Figure S41. (A) Cycling performance and (B-G) voltage-time curves of Sn-ZnF_2/Al-Na electrode tested at 8.0 mA cm^{-2} and 8.0 mAh cm^{-2}.

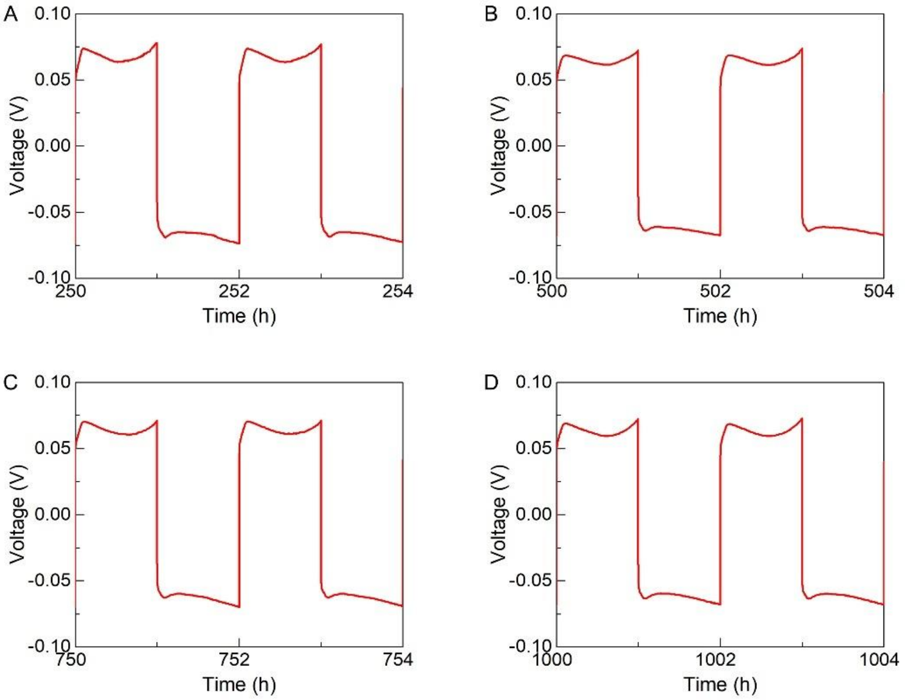

Figure S42. Voltage-time curves of Sn-ZnF_2/Al-Na electrode tested at 10.0 mA cm^{-2} and 10.0 mAh cm^{-2}.

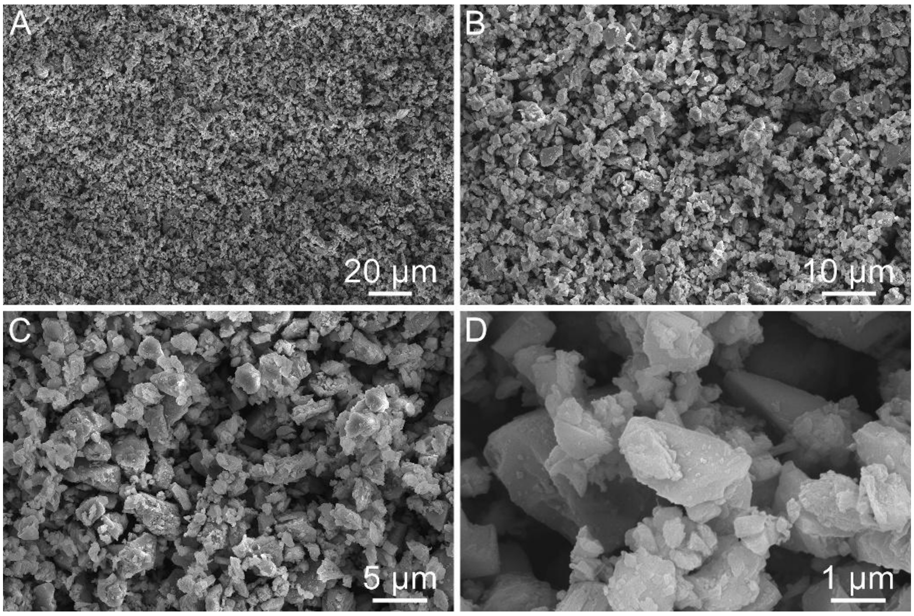

Figure S43. FESEM images of NVOPF.

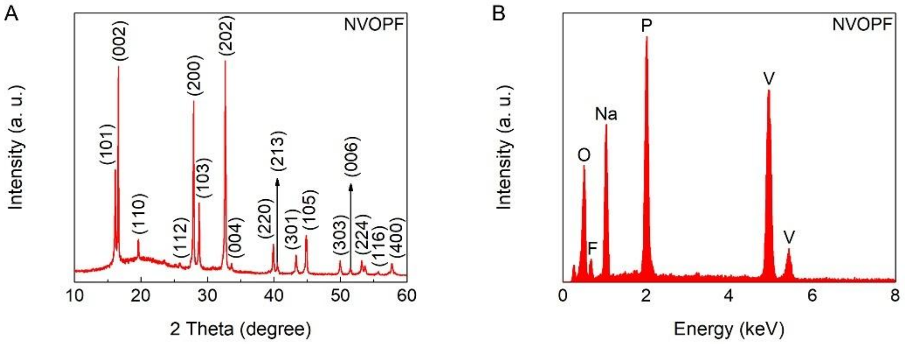

Figure S44. (A) XRD pattern and (B) EDX spectrum of NVOPF.

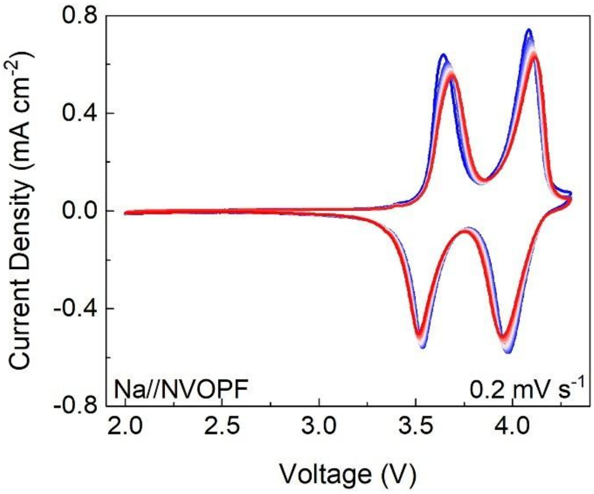

Figure S45. CV curves of Na//NVOPF.

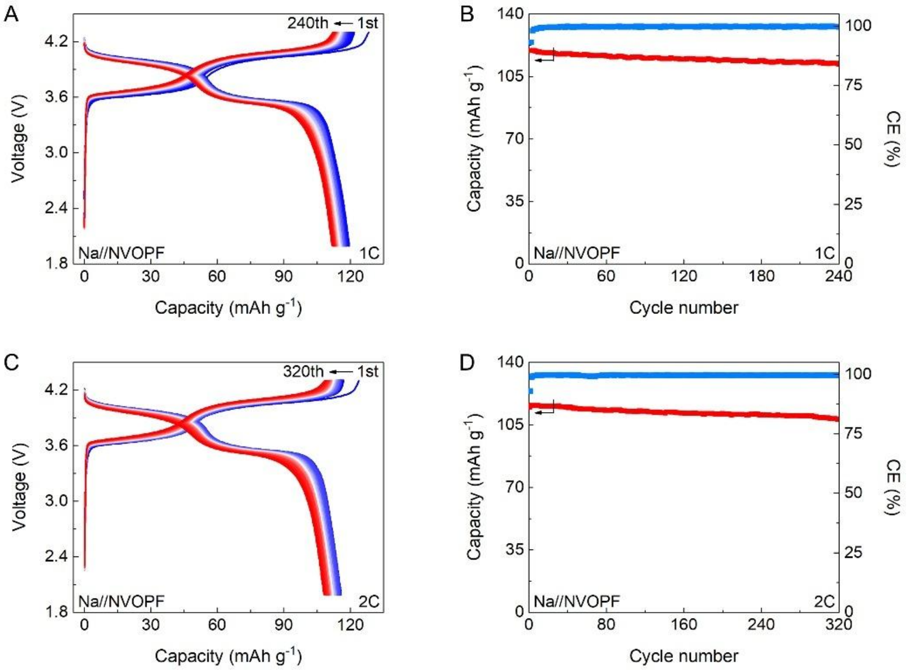

Figure S46. (A, C) Charge-discharge voltage curves and (B, D) cycling performance of Na//NVOPF cell tested at (A, B) 1C and (C, D) 2C.

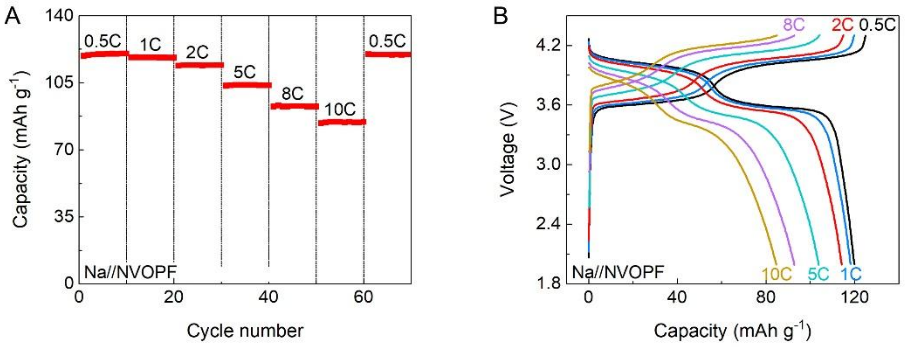

Figure S47. Rate performance of Na//NVOPF cell tested at different current densities.

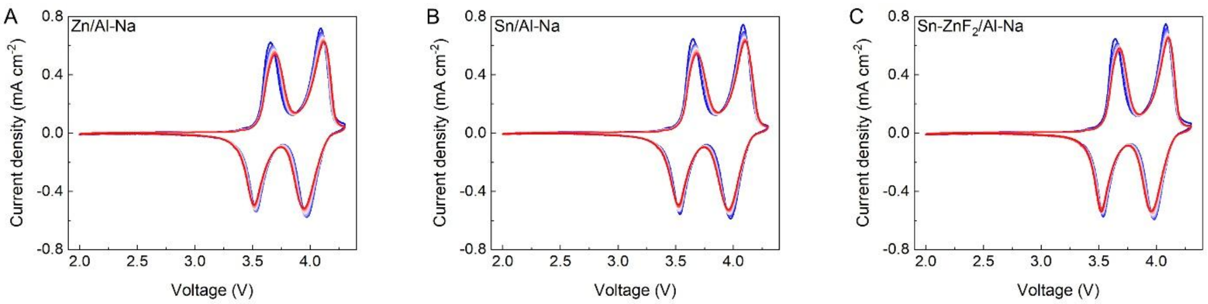

Figure S48. CV curves of different full cells tested at a scan rate of 0.2 mV s^{-1}. (A) Zn/Al-Na//NVOPF, (B) Sn/Al-Na//NVOPF, and (C) Sn-ZnF_2/Al-Na//NVOPF.

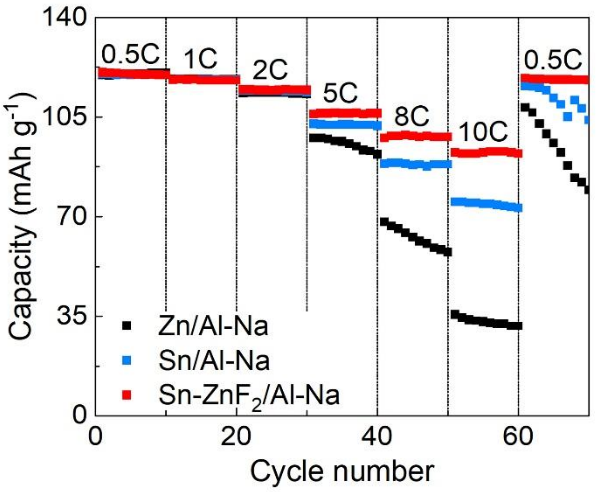

Figure S49. Rate capability of different full cells.

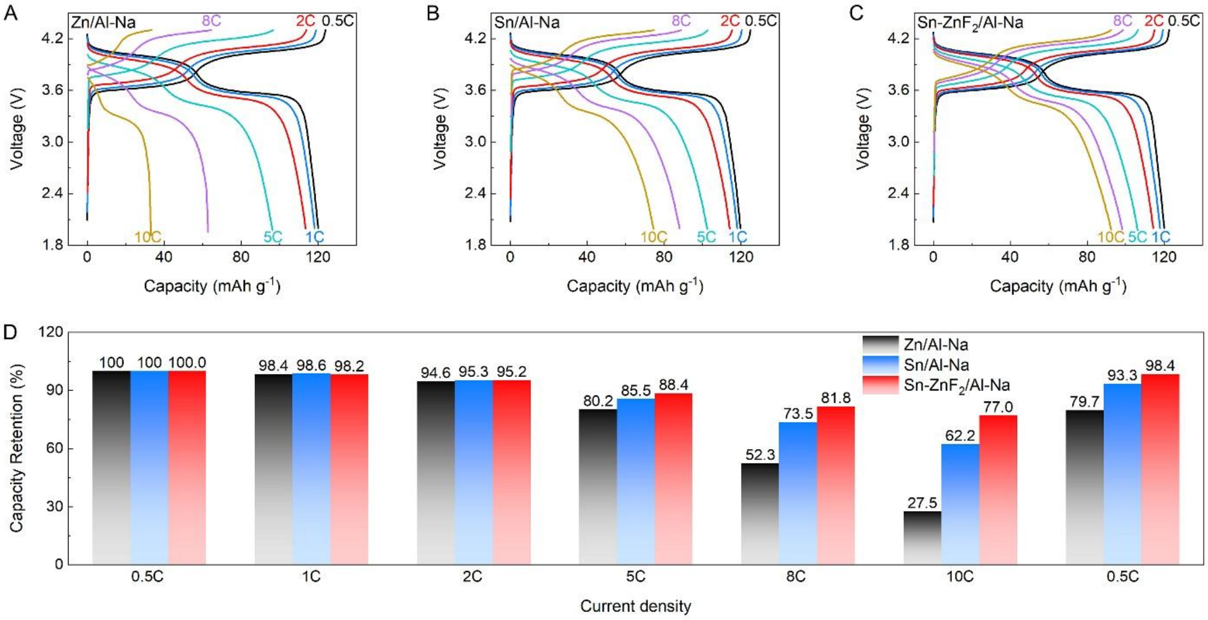

Figure S50. (A-C) Charge-discharge voltage curves and (D) capacity retentions of different full cells tested at different current densities. (A) Zn/Al-Na//NVOPF, (B) Sn/Al-Na//NVOPF, and (C) Sn-ZnF_2/Al-Na//NVOPF.

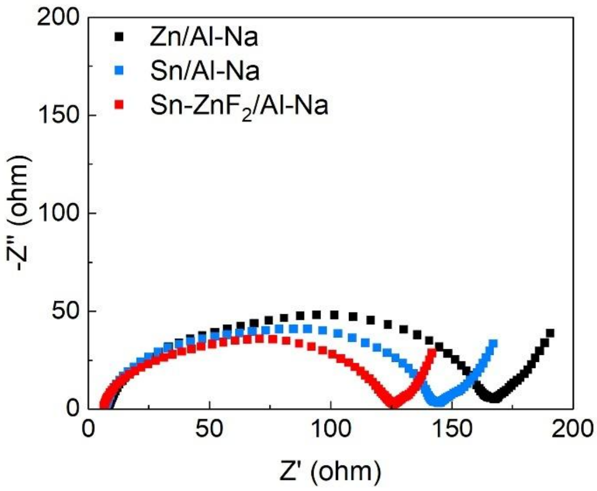

Figure S51. Nyquist plots of full cells after cycling.

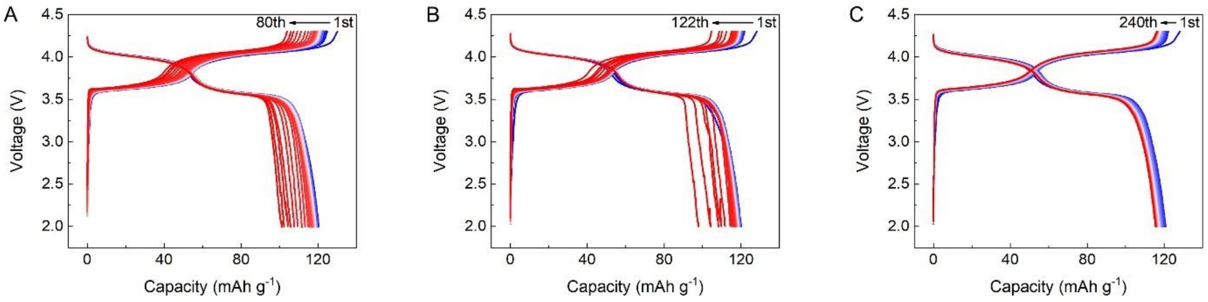

Figure S52. Charge-discharge voltage curves of different full cells tested at 1.0C. (A) Zn/Al-Na//NVOPF, (B) Sn/Al-Na//NVOPF, and (C) Sn-ZnF_2/Al-Na//NVOPF.

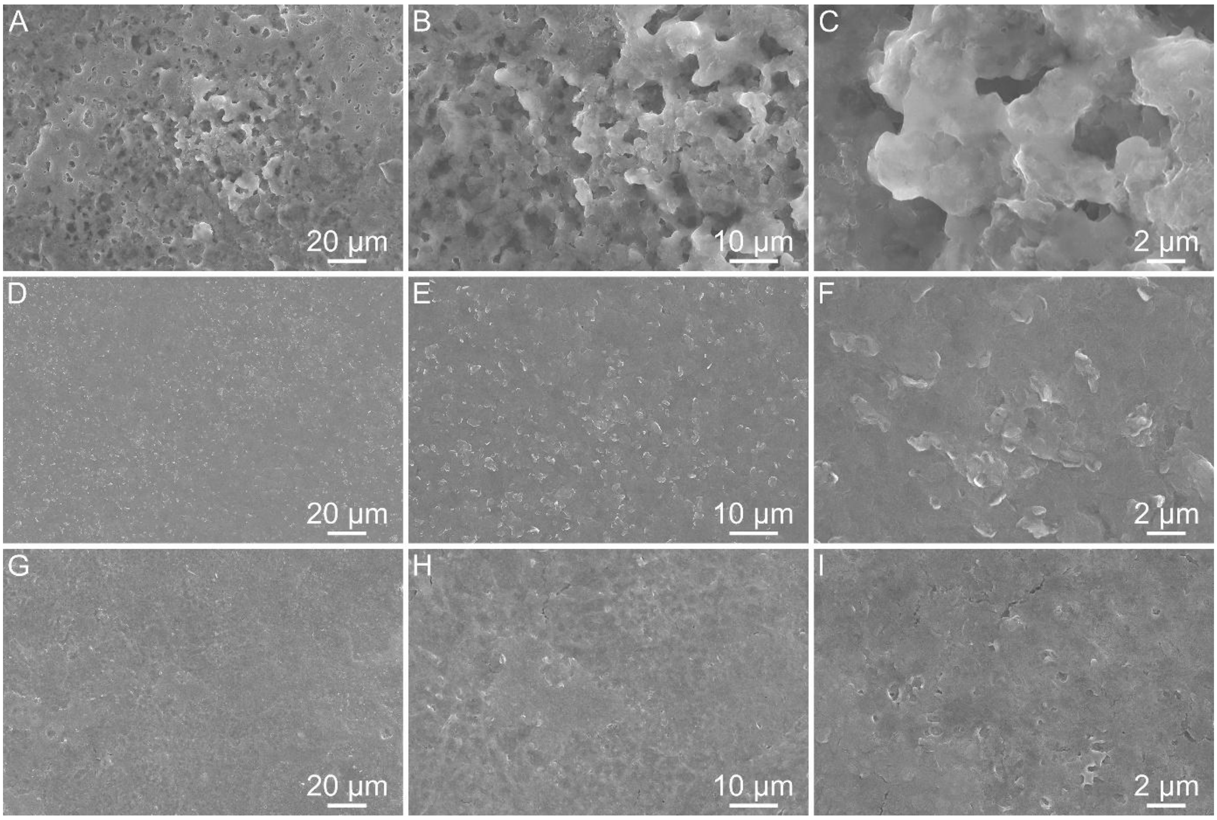

Figure S53. FESEM images of different anodes in full cells after cycling. (A-C) Zn/Al-Na, (D-F) Sn/Al-Na, and (G-I) Sn-ZnF_2/Al-Na.

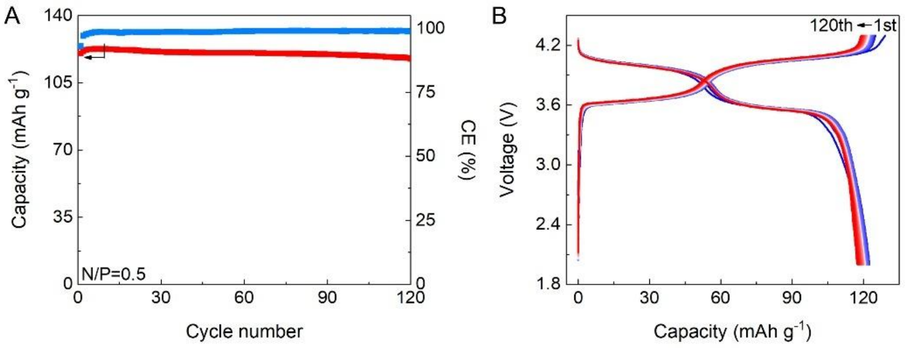

Figure S54. (A) Cycling performance and (B) charge-discharge voltage curves of Sn-ZnF_2/Al-Na//NVOPF cell (N/P = 0.5).

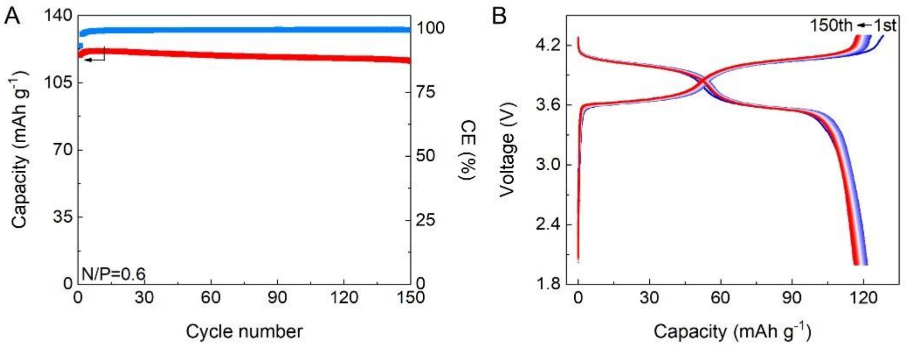

Figure S55. (A) Cycling performance and (B) charge-discharge voltage curves of Sn-ZnF_2/Al-Na//NVOPF cell (N/P = 0.6).

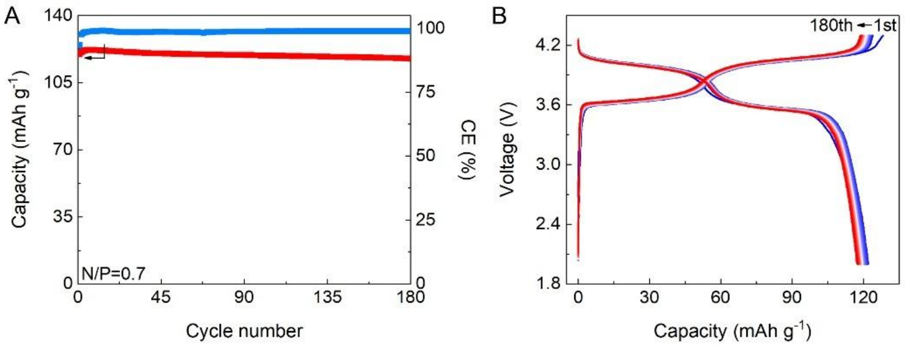

Figure S56. (A) Cycling performance and (B) charge-discharge voltage curves of Sn-ZnF_2/Al-Na//NVOPF cell (N/P = 0.7).

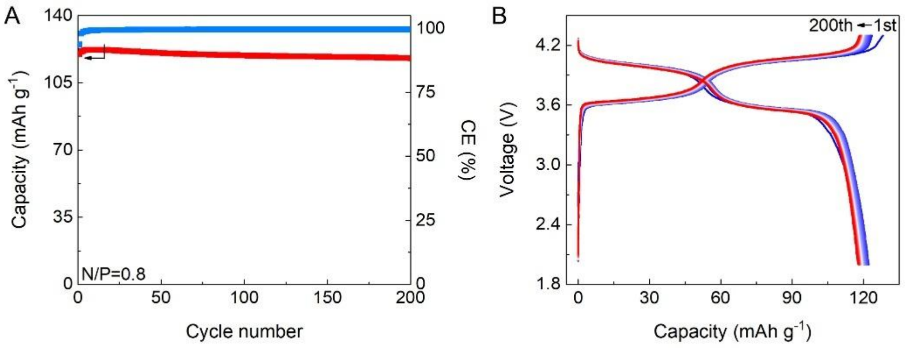

Figure S57. (A) Cycling performance and (B) charge-discharge voltage curves of Sn-ZnF_2/Al-Na//NVOPF cell (N/P = 0.8).

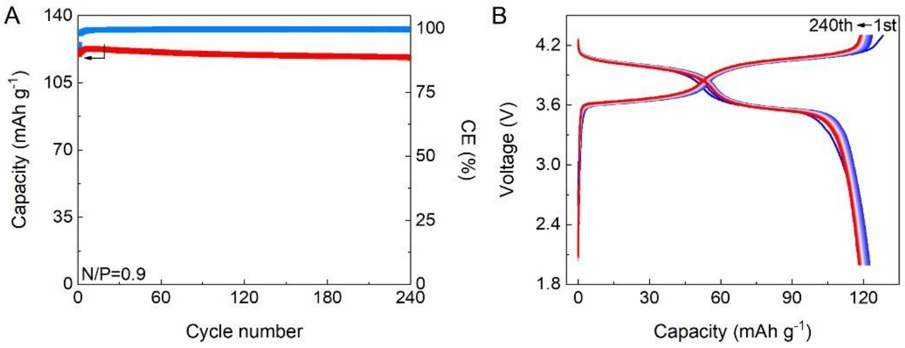

Figure S58. (A) Cycling performance and (B) charge-discharge voltage curves of Sn-ZnF_2/Al-Na//NVOPF cell (N/P = 0.9).

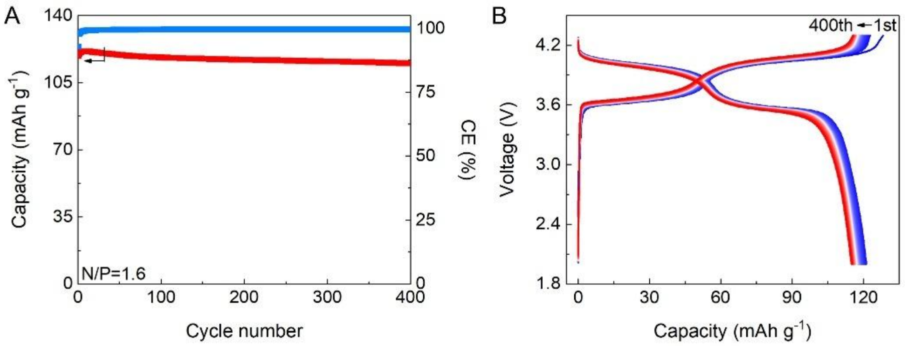

Figure S59. (A) Cycling performance and (B) charge-discharge voltage curves of Sn-ZnF_2/Al-Na//NVOPF cell (N/P = 1.6).

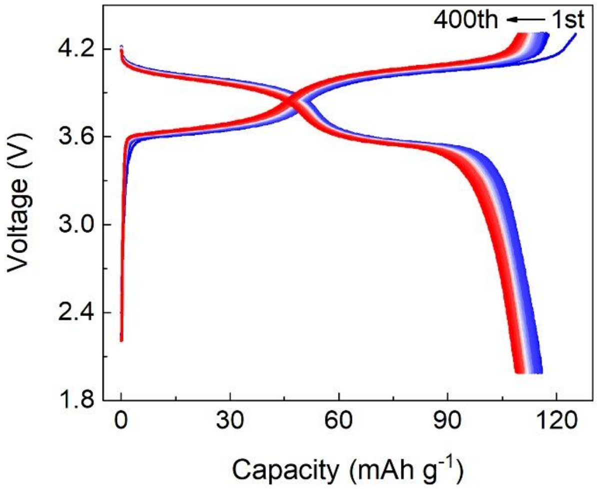

Figure S60. Charge-discharge voltage curves of Sn-ZnF_2/Al-Na//NVOPF cell tested at 2.0C.

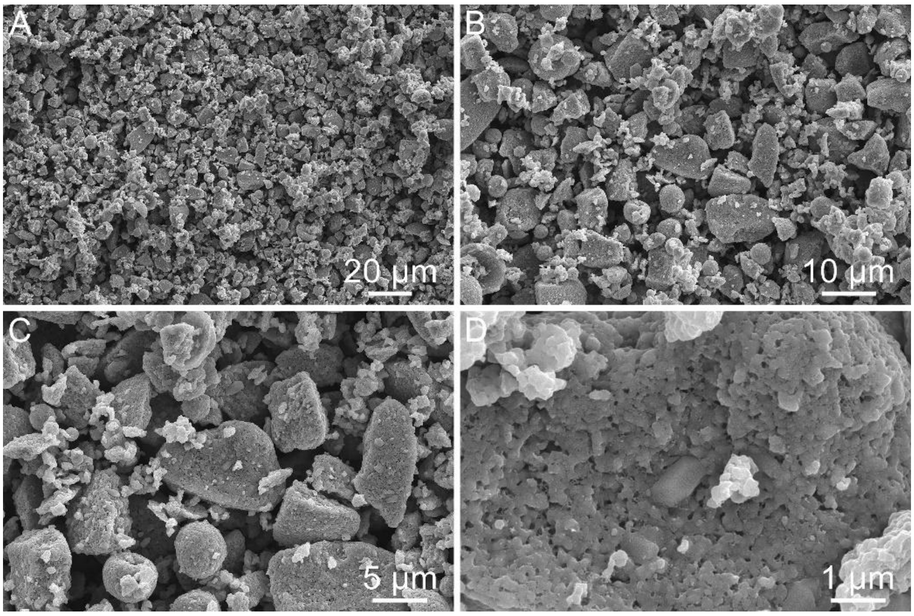

Figure S61. FESEM images of NFPP.

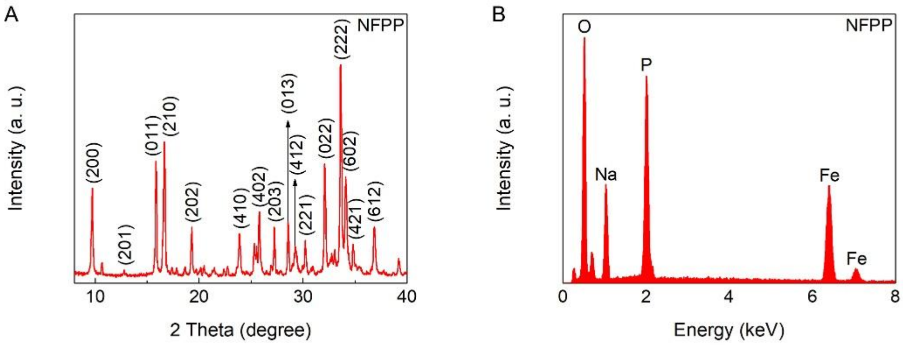

Figure S62. (A) XRD pattern and (B) EDX spectrum of NFPP.

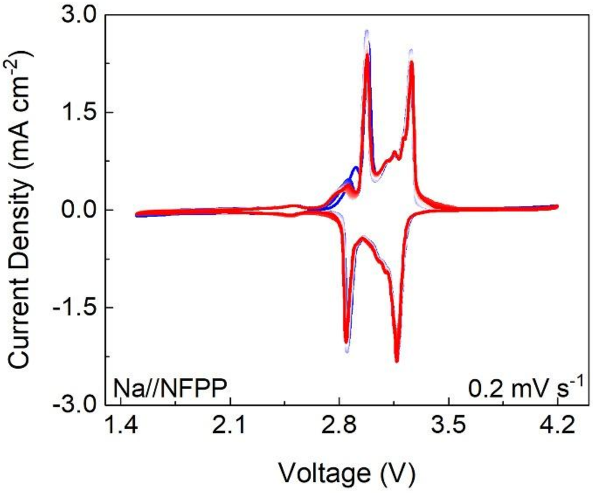

Figure S63. CV curves of Na//NFPP.

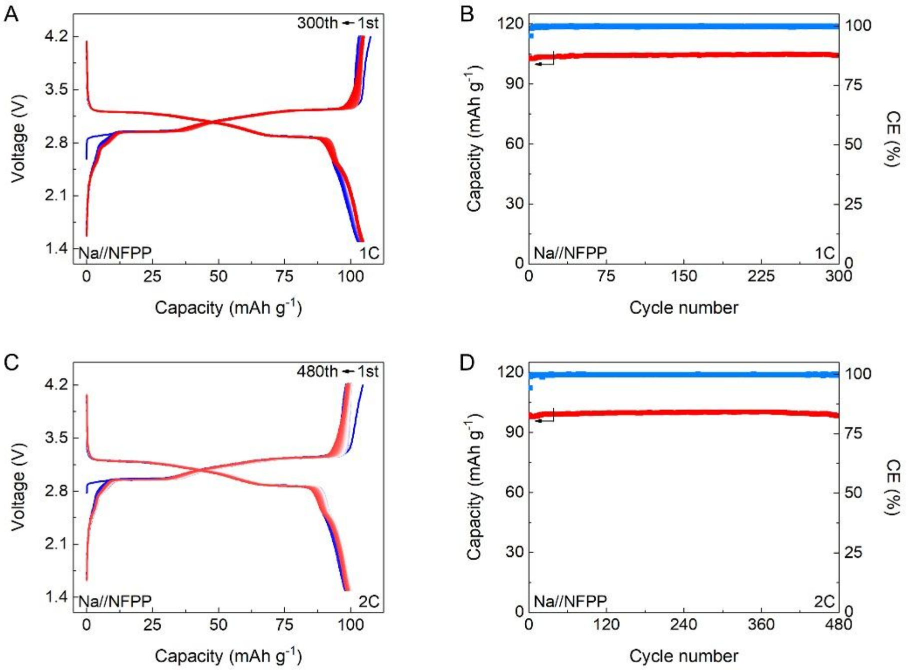

Figure S64. (A, C) Charge-discharge voltage curves and (B, D) cycling performance of Na//NFPP cell tested at (A, B) 1C and (C, D) 2C.

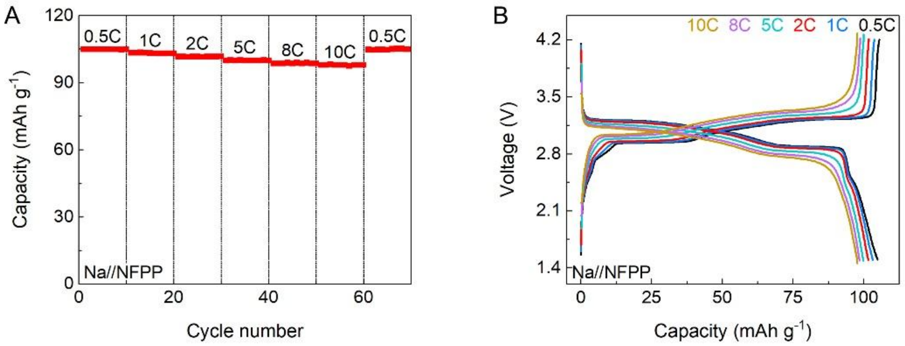

Figure S65. Rate performance of Na//NFPP cell tested at different current densities.

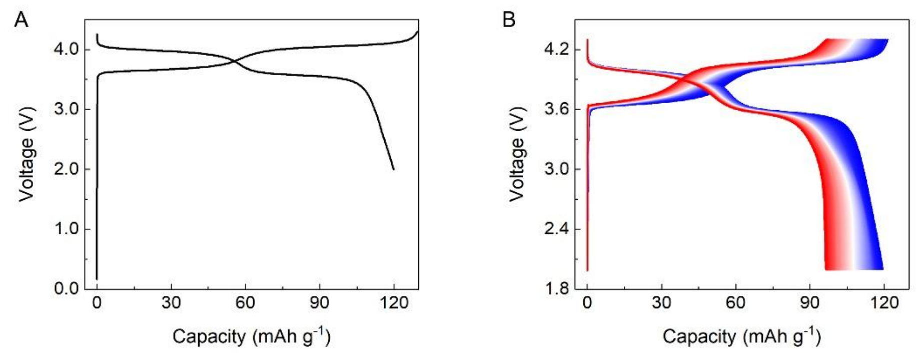

Figure S66. (A) 1st and (B) 2nd to 220th charge-discharge voltage curves of anode-free Sn-ZnF_2/Al//NVOPF pouch cell tested at 0.5C.

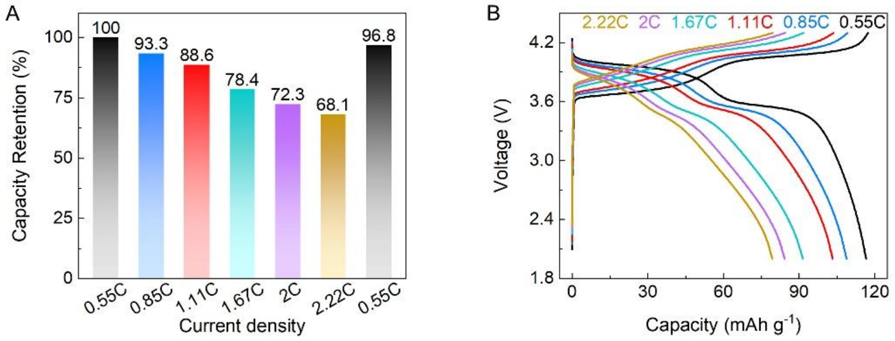

Figure S67. (A) Capacity retentions and (B) charge-discharge voltage curves of anode-free Sn-ZnF_2/Al//NVOPF pouch cell tested at different current densities.

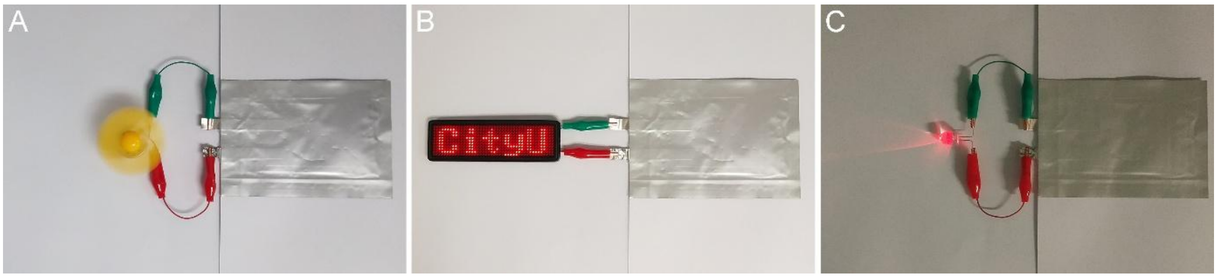

Figure S68. Optical images of fully charged anode-free Sn-ZnF_2/Al//NVOPF pouch cell to light up (A) a rotating fan, (B) an LED sign, and (C) an LED light.

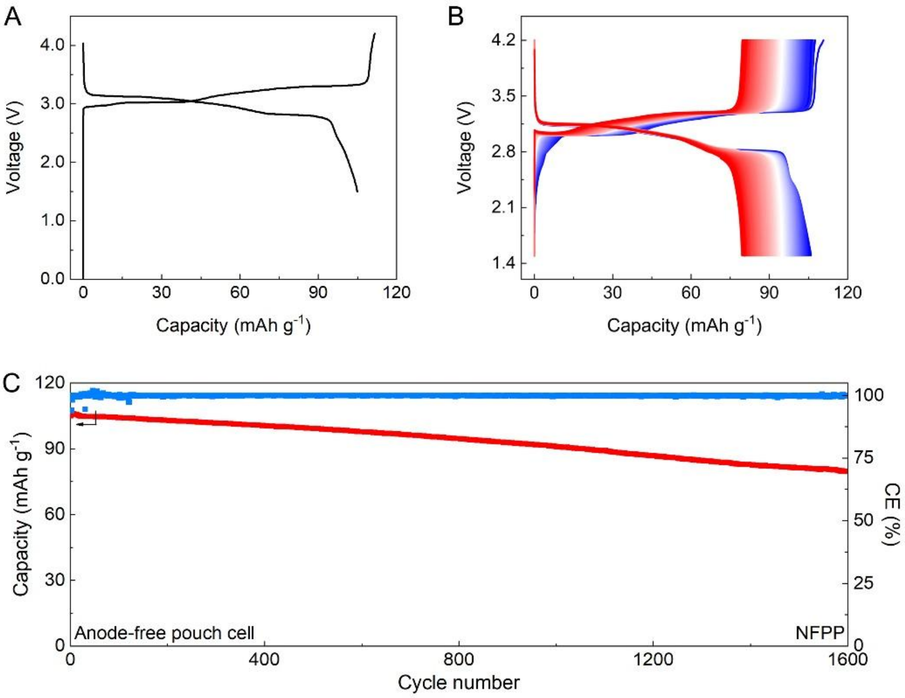

Figure S69. (A) 1st and (B) 2nd to 1600th charge-discharge voltage curves, and (C) cycling performance of anode-free Sn-ZnF_2/Al//NFPP pouch cell tested at 0.5C.

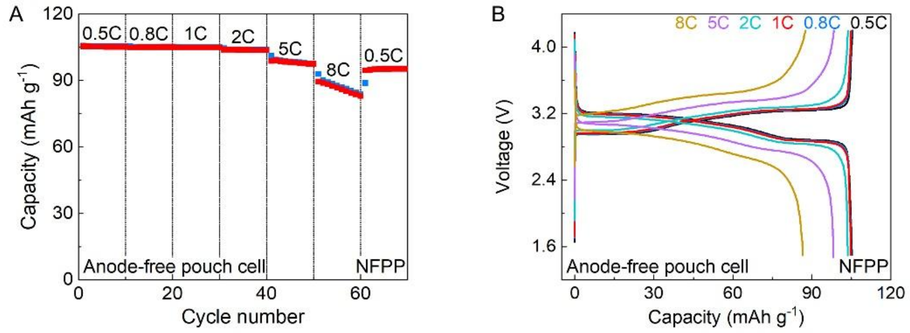

Figure S70. (A) Rate capability and (B) charge-discharge voltage curves of anode-free Sn-ZnF_2/Al//NFPP pouch cell tested at different current densities.

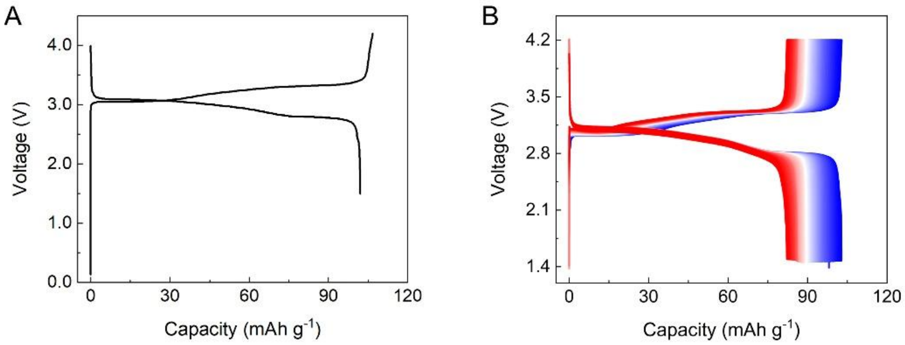

Figure S71. (A) 1st and (B) 2nd to 2600th charge-discharge voltage curves of anode-free Sn-ZnF_2/Al//NFPP pouch cell tested at 1.0C.

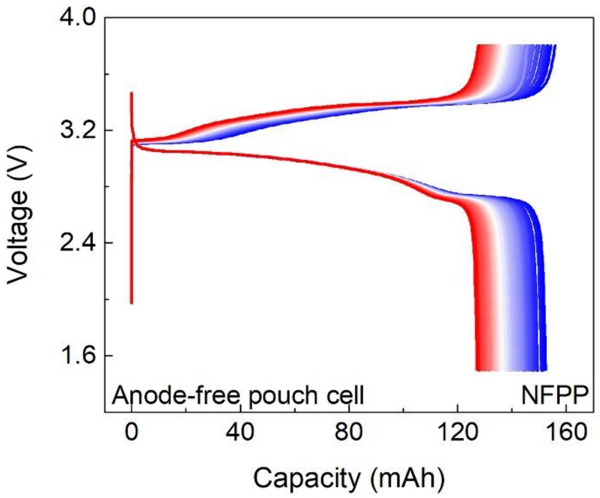

Figure S72. 10th to 60th charge-discharge voltage curves of 0.16 Ah-level anode-free Sn-ZnF_2/Al//NFPP pouch cell.

Table S1. Comparisons of Na deposition/dissolution performance of the Sn-ZnF_2/Al host tested at different current densities and areal capacities.

Current Density (mA cm^{-2})	Areal Capacity (mAh cm^{-2})	Cycle Number	Accumulated Capacity (mAh cm^{-2})	CE (%)
1.0	1.0	1800	1800	99.95
2.0	1.0	2380	2380	100.00
5.0	1.0	3260	3260	100.00
8.0	1.0	4440	4440	99.98
8.0	2.0	828	1656	99.99
8.0	5.0	220	1100	99.91
8.0	8.0	116	928	99.98
8.0	10.0	224	2240	99.96
10.0	1.0	2936	2936	99.97
10.0	2.0	644	1288	99.96
10.0	3.0	852	2556	99.99
10.0	4.0	620	2480	99.99
10.0	8.0	260	2080	99.98

Table S2. Comparisons of electrochemical performance of the Sn-ZnF_2/Al and other hosts reported previously.

Electrode	Electrolyte	Current Density (mA cm^{-2})	Areal Capacity (mAh cm^{-2})	Cycles	Accumulated Capacity (mAh cm^{-2})	CE (%)	Ref.
Cu	1M $NaPF_6$ in Diglyme	1	1	1200	1200	99.93	1
Cu	2.0 M $NaPF_6$ in DEE	1	1	150	150	99.89	2
Cu	1M $NaPF_6$ in Diglyme + SiO_2	1	2	500	1000	-	3
Cu	1.8M $NaPF_6$ in THF /2-MeTHF	1	1	400	400	99.91	4
Cu	1M $NaPF_6$ in Diglyme + MPE	5	5	1000	5000	99.95	5
Cu_3P@Cu	1M $NaPF_6$ in Diglyme	4	4	250	1000	99.12	6
N-CSs/Cu	1M $NaPF_6$ in Diglyme	2	2	900	1800	99.99	7
Bi_2Se_3-Cu	1M $NaPF_6$ in Diglyme	1	1	700	700	99.5	8
UGB-Cu	1M $NaPF_6$ in Diglyme	1	1	100	100	99.98	9
Bi-NAs@Cu	1M $NaPF_6$ in Diglyme	1	1	1000	1000	99.8	10
KJB@Cu	1M $NaPF_6$ in Diglyme	0.5	1	150	150	99.8	11
PBA@Cu	1M $NaPF_6$ in Diglyme	5	1	500	500	99.7	12
SnO_2@HMCNS @Cu	1M $NaPF_6$ in Diglyme	4	2	1800	3600	99.8	13
Ru/NC-Cu	1M $NaPF_6$ in Diglyme	1	1	1000	2000	100	14
AuPC@Cu	1M $NaPF_6$ in Diglyme	5	1	1000	1000	98	15
MRS-SbSA@Cu	1M $NaPF_6$ in DME	1	1	1800	1800	99.99	16
Cu@Au	1M $NaSO_3CF_3$ in Diglyme	2	2	300	600	99.8	17
Al	Na-SSZE	1	1	1000	1000	99.84	18
Al	1M $NaPF_6$ in DEBE	2	4	700	700	-	19
Porous Al	1M $NaPF_6$ in Diglyme	1	0.5	1000	500	99.8	20
GC@Al	1M $NaPF_6$ in Diglyme	1	1	400	400	99.8	21
Carbon/Al	1M $NaPF_6$ in Diglyme	0.5	0.25	1000	250	99.8	22
Al~HC	1M $NaPF_6$ in Diglyme	6	1	2500	2500	99.75	23

Electrode	Electrolyte	Current Density (mA cm^{-2})	Areal Capacity (mAh cm^{-2})	Cycles	Accumulated Capacity (mAh cm^{-2})	CE (%)	Ref.
C@Al	0.7M $NaPF_6$ in Diglyme + ANI	0.5	0.5	1000	500	99.98	24
p-Al@C	0.6 M NaOTF + 0.4 M $NaBF_4$ in Diglyme	0.5	0.5	100	50	99.88	25
Al/C	TGPE	0.5	0.5	400	200	99.92	26
BTO/C-Al	$Na_3Zr_2Si_2PO_{12}$	0.1	1	165	165	98.2	27
E-Zn@Al	1M $NaPF_6$ in Diglyme	3	3	1000	3000	99.95	28
SnCu@Al	1M $NaPF_6$ in Diglyme	1	1	500	500	99.95	29
D-ZnO NRAs/Al	1M $NaPF_6$ in Diglyme	10	20	100	2000	100	30
Al(100)	1M $NaPF_6$ in DME	4	4	100	400	99.9	31
3D Zn@Al	1 M $NaPF_6$ in DME	2	2	500	1000	99.5	32
NbMoTaWV@Al	1 M $NaPF_6$ in DME	2	2	1000	2000	99.5	33
F-Al	1M $NaPF_6$ in DEGDME	0.5	0.5	100	50	-	34
PC-CFe	1M $NaPF_6$ in Diglyme	10	10	500	5000	99.6	35
CeF_3@NC	1M $NaPF_6$ in Diglyme	2	2	500	1000	99.55	36
Al-Cu@C	1M $NaPF_6$ in Diglyme	0.5	1	60	60	97.5	37
O-CCF	1M $NaPF_6$ in Diglyme	5	10	1000	10000	99.6	38
FCTF	1M $NaPF_6$ in Diglyme	2	1	400	400	99.6	39
SnNCNFs	1M $NaPF_6$ in Diglyme	3	3	2000	6000	99.96	40
Zn-CNF	1M $NaPF_6$ in Diglyme	1	1	1600	1600	99.94	41
FMCNF	1M $NaPF_6$ in Diglyme	5	1	5000	5000	99.93	42
CoP@N/P-CMFs	1M $NaPF_6$ in Diglyme	10	10	330	3300	99.97	43
MgF_2@NCHNFs	1M $NaPF_6$ in Diglyme	5	1	2000	2000	99.9	44
NOCS	0.9 M $NaPF_6$ + 0.1 M $NaBF_4$ in Diglyme	2	2	1000	2000	99.97	45
N-Sb@M-Sb@CNT@C	1M $NaPF_6$ in DME	20	1	8000	8000	99.96	46
Bi-N_3S_1@CT	1M $NaPF_6$ in DME	12	6	900	5400	99.6	47
Sn-ZnF_2/Al	1M $NaPF_6$ in Diglyme	10	8	260	2080	99.98	This Work

Table S3. Comparisons of cycling performance of the Sn-ZnF_2/Al-Na electrode with different current densities and areal capacities.

Current Density (mA cm^{-2})	Areal Capacity (mAh cm^{-2})	Cycle Number	Accumulated Capacity (mAh cm^{-2})	Time (h)
1.0	1.0	750	750	1500
2.0	2.0	1100	2200	2200
5.0	5.0	1050	5250	2100
8.0	8.0	800	6400	1600
10.0	10.0	500	5000	1000

Table S4. Comparisons of electrochemical performance of the Sn-ZnF_2/Al-Na and other composite Na anodes reported previously.

Electrode	Electrolyte	Current Density (mA cm^{-2})	Areal Capacity (mAh cm^{-2})	Depth-of-Discharge (%)	Accumulated Capacity (mAh cm^{-2})	Life (h)	Ref.
Na	1M $NaPF_6$ in Diglyme	1	1	-	1500	3000	1
Na	1.8M $NaPF_6$ in THF + 2-MeTHF	2	2	-	5000	5000	4
Na	1M $NaPF_6$ in Diglyme + MPE	5	5	75	3500	1400	5
Cu_3P@Cu-Na	1M $NaPF_6$ in Diglyme	2	2	-	2000	2000	6
N-CSs-Na	1M $NaPF_6$ in Diglyme	2	2	-	1500	1500	7
Bi_2Se_3-Na	1M $NaPF_6$ in Diglyme	10	10	-	3000	600	8
UGB-Cu-Na	1M $NaPF_6$ in Diglyme	1	1	95.23	68	136	9
Bi-NAs@Cu-Na	1M $NaPF_6$ in Diglyme	1	3	75	600	1200	10
PBA@Cu-Na	1M $NaPF_6$ in Diglyme	5	1	66.67	1500	600	12
SnO_2@HMCNS @Cu-Na	1M $NaPF_6$ in Diglyme	8	4	-	2800	700	13
MRS-SbSA@Cu-Na	1M $NaPF_6$ in DME	2	2	91	2000	2000	16
Na	1M $NaPF_6$ in DEBE	3	6	-	15000	1000<br>0	19
Porous Al-Na	1M $NaPF_6$ in Diglyme	0.5	0.5	25	250	1000	20
Na	0.7M $NaPF_6$ in Diglyme + ANI	0.5	0.5	-	250	1000	24
Al/C-Na	TGPE	1	1	-	300	600	26
BTO/C-Al-Na	$Na_3Zr_2Si_2PO_{12}$	0.5	0.5	-	125	500	27
E-Zn@Al-Na	1M $NaPF_6$ in Diglyme	3	3	-	1500	1000	28
SnCu@Al-Na	1M $NaPF_6$ in Diglyme	5	5	80	3750	1500	29
D-ZnO NRAs/Al-Na	1M $NaPF_6$ in Diglyme	10	10	50	10000	2000	30
Al(100)-Na	1M $NaPF_6$ in DME	1	1	-	750	1500	31
3D Zn@Al-Na	1 M $NaPF_6$ in DME	2	1	50	1500	1500	32
NbMoTaWV@ Al	1 M $NaPF_6$ in DME	2	1	50	1000	1000	33
PC-CFe-Na	1M $NaPF_6$ in Diglyme	5	5	-	4125	1650	35

Electrode	Electrolyte	Current Density (mA cm^{-2})	Areal Capacity (mAh cm^{-2})	Depth-of-Discharge (%)	Accumulated Capacity (mAh cm^{-2})	Life (h)	Ref.
CeF_3@NC-Na	1M $NaPF_6$ in Diglyme	2	2	40	2100	2100	36
O-CCF-Na	1M $NaPF_6$ in Diglyme	50	1	16.67	10000	400	38
FCTF-Na	1M $NaPF_6$ in Diglyme	2	2	100	140	140	39
SnNCNFs-Na	1M $NaPF_6$ in Diglyme	10	10	86	3500	700	40
Zn-CNF-Na	1M $NaPF_6$ in Diglyme	1	1	-	2200	4400	41
FMCNF-Na	1M $NaPF_6$ in Diglyme	10	10	94	10000	2000	42
CoP@N/P-CMFs-Na	1M $NaPF_6$ in Diglyme	8	8	50	3200	800	43
MgF_2@NCHNFs-Na	1M $NaPF_6$ in Diglyme	5	2	16.7	9000	3600	44
NOCS-Na	0.9 M $NaPF_6$ + 0.1 M $NaBF_4$ in Diglyme	0.5	1	50	250	1000	45
N-Sb@M-Sb@CNT@C-Na	1M $NaPF_6$ in DME	4	4	-	17520	8760	46
Bi-N_3S_1@CT-Na	1M $NaPF_6$ in DME	10	10	83	5000	1000	47
NST-Na	1M $NaPF_6$ in Diglyme	2	10	60	1500	1500	48
F-A-Al-Na	Na[FSA] in [C2C1im][FSA]	0.5	0.5	25	150	600	49
HCOONa-Na	1M $NaPF_6$ in Diglyme	2	1	-	2200	2200	50
Sn-ZnF_2/Al-Na	1M $NaPF_6$ in Diglyme	10	10	50	5000	1000	This Work

Table S5. Comparisons of electrochemical performance of the anode-less Sn-ZnF_2/Al-Na//NVOPF cell with previously reported works.

Anode	Cathode	Mass Loading (mg cm^{-2})	N/P Ratio	Voltage (V)	Current Density (mA g^{-1})	Capacity (mAh g^{-1})	Cycle Number	Capacity Retention (%)	Ref.
N-CSs	NNMO	4	-	2.5-3.9	-	-	200	86.3	7
Bi_2Se_3-Na	NVP	2	-	2.5-3.7	2340	-	1500	93.7	8
Bi-NAs@Cu-Na	NTP	6	1	1.5-2.5	133	95.18	267	93.22	10
KJB@Cu-Na	NVP	-	-	2.5-3.8	60	-	150	94.16	11
PBA@Cu-Na	NVP	3	3	2.2-3.8	590	-	1500	94.3	12
Na	NMC622	22	1.7	2.0-4.3	-	- -	500	80.9	19
E-Zn@Al-Na	NVP	10	1.5	2.5-3.8	117	- -	600	99	28
SnCu@Al-Na	NVP	4.5	1	2.5-3.8	117	- -	400	93	29
D-ZnO NRAs/Al-Na	NVOPF	32.26	1.5	2.0-4.3	130	- 102.5	105	87.1	30
CeF_3@NC-Na	NVP	7.52	2	2.5-3.8	58.5	- 105.7	250	99.78	36
FCTF-Na	NVP	6.4	1.5	2.5-3.8	236	108.9	300	94.7	39
SnNCNF-Na	S@pPAN	10	1.5	0.6-2.8	750	-	80	81	40
Zn-CNF-Na	NVP	-	-	2.5-3.8	234	-	250	96.53	41
FMCNF-Na	NVP	12	2.1	2.5-3.8	234	-	600	91	42
NOCS-Na	NVP	11.4	1.5	2.0-3.8	500	100	800	96.2	45
Bi-N_3S_1@CT-Na	NVP	5.4	1.68	2.5-3.8	220	97	400	96.7	47
Zn-CNF-Na	NVP	-	-	2.5-3.8	234	-	250	96.53	41
Sn-ZnF_2/Al-Na	NVOPF	1.7	1.0	2.0-4.3	130	115.6	240	97.54	This Work

Table S6. Comparisons of electrochemical performance of the anode-free Sn-ZnF_2/Al//NVOPF and Sn-ZnF_2/Al//NFPP pouch cells with previously reported works.

Anodic Current Collector	Cathode	Mass Loading (mg cm^{-2})	Voltage (V)	Current Density (mA g^{-1})	Capacity (mAh g^{-1})	Cycle Number	Capacity Retention (%)	Ref.
Al-C	NVP	5	2.2-3.8	234	98.43	600	95.06	1
Cu	NFM	4.5	2.0-3.8	70	-	100	82.3	2
Cu	NVP	4	2.6-3.8	0.5 mA cm^{-2}	71.88	100	75	3
Al/C	NFM	14.05	2.0-3.8	24	-	150	91	4
C@Al	NVP	15	2.7-3.8	110	80	480	80	5
Cu_3P@Cu	NVP	13.6	2.5-3.5	60	76.1	75	-	6
N-CPs	NNMO	4	2.5-3.8	50	-	200	86.3	7
Bi_2Se_3-Cu	NVP	10	2.5-3.7	117	75.1	100	-	8
UGB-Cu	NVP	~10	3.0-3.6	585	73.2	800	76.41	9
PBA@Cu	NVP	3	2.2-3.8	590	-	300	75.29	12
SnO_2@HMC NS@Cu	NVP	3.5	2.8-4.0	100	-	200	79.5	13
Ru/NC-Cu	NVP	3	2.5-3.6	50	-	100	98.1	14
MRS-SbSA@Cu	NVP	7	2.0-3.6	117	-	100	83	16
Cu@Au	Na-FeS_2	-	0.8-3.0	-	140	50	-	17
C@Al	NVPOF	7.1	2.0-4.25	0.49 mA cm^{-2}	97.9	370	89.2	18
Al	NMC622	22	2.0-4.3	-	120	300	78	19
Porous Al	Na-TiS_2	-	1.3-2.6	0.1 mA cm^{-2}	~160	200	~64	20
GC@Al	NVPOF	10	2.0-4.3	-	82.44	250	-	21
Carbon/Al	$Na_{1.5}FeS_2$	5	0.8-3.0	0.125 mA cm^{-2}	335	40	-	22
Al~HC	NFPP	5	1.5-3.5	20	84.6	100	90.5	23
C@Al	NVP	10	2.7-3.8	-	-	220	70	24
Al/C	NVP	10	2.5-3.8	117	-	500	79	26
BTO/C-Al	NVP	-	2.5-3.8	0.1 mA cm^{-2}	104.1	300	95.5	27
E-Zn@Al	NVP	32	2.5-3.8	3.74 mA cm^{-2}	103	90	86	28
SnCu@Al	NVP	4.6	2.5-3.8	117	64.5	200	71	29

Anodic Current Collector	Cathode	Mass Loading (mg cm^{-2})	Voltage (V)	Current Density (mA g^{-1})	Capacity (mAh g^{-1})	Cycle Number	Capacity Retention (%)	Ref.
D-ZnO NRAs/Al	NVOPF	5.1	2.0-4.3	65	104.2	105	86	30
Al(100)	NVP	1.5	2.5-3.6	1.755 mA cm^{-2}	68	100	-	31
3D Zn@Al	NVP	1.8	2.5-3.8	59	-	100	98.8	32
NbMoTaWV @Al	NVP	1.8	2.5-3.8	-	-	300	96.5	33
F-Al	Fe-PB	15.6	2.0-3.8	-	105.1	80	76.0	34
PC-CFe	NVP	10	2.6-3.8	1 mA cm^{-2}	103	100	97	35
CeF_3@NC	NVP	9.23	2.5-3.8	23.4	-	100	83.6	36
Al-Cu@C	NVP/C	3.5-4.0	1.5-3.8	120	-	50	-	37
O-CCF	NVP	11	2.5-4.0	1 mA cm^{-2}	103.7	100	96	38
FCTF	NVP	7.4	2.5-3.8	240	-	400	56	39
SnNCNF	NVP	10	2.6-3.8	234	89.3	80	89	40
Zn-CNF	NVP	7.97	2.5-3.8	58.5	82.49	120	80.4	41
FMCNF	NVP	20	2.5-3.8	35.1	-	200	90	42
CoP@N/P-CMFs	NVP	18	2.0-3.0	117	82.7	120	87.38	43
MgF_2@NCHNFs	NVP	8	2.5-3.8	236	-	50	91.2	44
NOCS	NVP	12.3	2.0-3.8	200	81.4	350	79.0	45
N-Sb@M-Sb@CNT@C	NVP	11.13	2.3-3.6	40	91.75	500	81.92	46
Bi-N_3S_1@CT	NVP	7.9	2.5-3.8	110	70	100	91.3	47
NST	NVP	2.5	3.2-3.6	118	73.15	100	77	48
F-A-Al	NVP	11.3	2.4-3.6	35.1	-	50	46.1	49
SF-Cu	NVP	10	2.8-3.7	58.5	86.3	400	88	50
Zn-CNF	NVP	7.97	2.5-3.8	58.5	82.49	120	80.4	41
Al/C	NVP	9	2.0-3.8	117/1170	-	500	72	51
C@Al	NNCFM	8.52	2.0-4.0	30	63.9	250	-	52
GC@Al	NCNFM	15.12	2.0-3.8	63.5	-	260	84	53

Anodic Current Collector	Cathode	Mass Loading (mg cm^{-2})	Voltage (V)	Current Density (mA g^{-1})	Capacity (mAh g^{-1})	Cycle Number	Capacity Retention (%)	Ref.
Al@C	NFPP	~12.4	1.5-4.0	258	93.3	300	-	54
Sn-ZnF_2/Al	NVOPF	3.25	2.0-4.3	65	96.3	220	80.34	This Work
Sn-ZnF_2/Al	NFPP	9.17	1.5-4.2	129	82.2	2600	80.59	This Work

REFERENCES

1. Hu, Z.; Liu, L.; Wang, X.; Zheng, Q.; Lu, H.; Tang, Z.; Han, C.; Li, W. Enhancing Low-Temperature Durability and Sodium-Ion Transport of Anode-Free Sodium Metal Batteries Through Utilization of a Solvent Adsorption Separator. *Energy Environ. Sci.* **2025,** *18,* 10048-10060.
2. Zou, Y.; Zhang, B.; Luo, H.; Yu, X.; Yang, M.; Zheng, Q.; Wang, J.; Jiao, C.; Chen, Y.; Zhang, H.; Xue, J.; Kuai, X.; Liao, H. G.; Ouyang, C.; Ning, Z.; Qiao, Y.; Sun, S. G. Electrolyte Solvation Engineering Stabilizing Anode-Free Sodium Metal Battery With 4.0 V-Class Layered Oxide Cathode. *Adv. Mater.* **2024,** *36*, 2410261.
3. Panchal, R. A.; Datta, J.; Varude, V.; Bhimani, K.; Mahajani, V.; Kamble, M.; Anjan, A.; Manoj, R. M.; Zha, R. H.; Datta, D.; Koratkar, N. Nano-Silica Electrolyte Additive Enables Dendrite Suppression in an Anode-Free Sodium Metal Battery. *Nano Energy* **2024,** *129*, 110010.
4. Xing, Q.; Lee, J. M.; Yang, Z.; Van Lehn, R. C.; Liu, F. Directing Selective Solvent Presentations at Electrochemical Interfaces to Enable Initially Anode-Free Sodium Metal Batteries. *Nat. Commun.* **2025,** *16*, 8265.
5. Huang, J.; An, X.; Cheng, Z.; Li, L.; Dou, S. X.; Liu, H. K.; Wu, C. Dipole-Dipole Interaction-Induced Dense Primitive Solid-Electrolyte Interphase for High-Power Ah-Level Anode-Free Sodium Metal Batteries. *Nat. Commun.* **2025,** *16*, 8586.
6. Zhang, W.; Zheng, J.; Ren, Z.; Wang, J.; Luo, J.; Wang, Y.; Tao, X.; Liu, T. Anode-Free Sodium Metal Pouch Cell Using Cu_3P Nanowires In Situ Grown on Current Collector. *Adv. Mater.* **2024,** *36*, 2310347.
7. Huang, B.; Sun, S.; Wan, J.; Zhang, W.; Liu, S.; Zhang, J.; Yan, F.; Liu, Y.; Xu, J.; Cheng, F.; Xu, Y.; Lin, Y.; Fang, C.; Han, J.; Huang, Y. Ultrahigh Nitrogen Content Carbon Nanosheets for High Stable Sodium Metal Anodes. *Adv. Sci.* **2023,** *10*, 2206845.
8. Gao, W.; Lu, Y.; Cao, J.; Xu, S.; Lu, Y.; Liu, X.; Liu, D.; Wu, Y.; He, J. Regulating Interface Chemistry with Topological Insulator for Ultra-Stable and Dendrite-Free Sodium Metal Batteries. *Small* **2025,** *21*, e06772.
9. Chen, Y.; Li, H.; Tu, S.; Song, J.; Ye, C.; Qiao, S. Z. Grain-Optimized Copper Current Collectors for Highly Stable Anode-Free Sodium Batteries. *Adv. Mater.* **2026**, *38*, e22541.

10. Bai, Y.; Zheng, X.; Liu, H.; Huang, J.; Zhang, L.; Otitoju, T. A.; Sun, T.; Liu, H. K.; Dou, S. X.; Wu, C. Honeycomb-Like Superstructure of 3D Sodiophilic Host for Anode-Free Sodium Batteries. *Energy Storage Mater.* **2025,** *74*, 103926.
11. Kang, S.; Geng, F.; Li, Z.; Jiang, Y.; Shen, M.; Chen, Q.; Lou, X.; Hu, B. Progressive Self-Leveling Deposition Improves the Cyclability of Anode-Less Sodium Metal Batteries Revealed by In Situ EPR Imaging. *ACS Energy Lett.* **2024,** *9*, 1633-1638.
12. Hu, Z.; Liu, L.; Wang, X.; Lu, H.; Zheng, Q.; Gao, Y.; Wang, J.; Qi, Y.; Han, C.; Li, W. In Situ Integration of Rapid Ion-Diffusion Interlayers on Cu Current Collectors Toward Ultrafast Anode-Free Sodium Metal Batteries. *ACS Nano* **2025,** *19*, 23193-23208.
13. Zhu, H.; Yan, Z.; Wang, X.; Liu, W.; Guo, L.; Wei, Q.; Wang, M. S. Synergistic Effect of Mesoporous Carbon-Based Framework with Sodiophilic Nanoparticles for Stable Sodium Metal Anodes. *Adv. Funct. Mater.* **2025,** *35*, 2502032.
14. Hao, C.; Zhang, X.; He, Z.; Gao, M.; Liu, Y.; Pan, H.; Sun, W. Robust Solid Electrolyte Interphase Engineered by Catalysis Chemistry Toward Durable Anode-Free Sodium Metal Batteries. *Angew. Chem. Int. Ed.* **2025,** *64*, e202503691.
15. Kim, S.; Ryoo, G.; Lee, J. H.; Kim, J.; Park, J. H.; Cho, K. Horizontal Sodium Growth via the Sodiophilicity-Driven Structural Design of Current Collectors for Anode-Free Sodium Metal Batteries. *ACS Nano* **2025,** *19*, 25455-25465.
16. Zhao, S.; Chen, X.; Wang, Y.; Hong, Z.; Zheng, L.; Zhang, Y.; Wei, M.; Lu, J. Highly Reversible Sodium Metal Batteries Enabled by Extraordinary Alloying Reaction of Single-Atom Antimony. *Adv. Energy Mater.* **2024,** *15*, 2403432.
17. Tang, S.; Qiu, Z.; Wang, X.-Y.; Gu, Y.; Zhang, X.-G.; Wang, W.-W.; Yan, J.-W.; Zheng, M.-S.; Dong, Q.-F.; Mao, B.-W. A Room-Temperature Sodium Metal Anode Enabled by a Sodiophilic Layer. *Nano Energy* **2018,** *48*, 101-106.
18. Lu, Z.; Yang, H.; Wu, G.; Shan, P.; Lin, H.; He, P.; Zhao, J.; Yang, Y.; Zhou, H. A “Liquid-In-Solid” Electrolyte for High-Voltage Anode-Free Rechargeable Sodium Batteries. *Adv. Mater.* **2024,** *36*, 2404569.
19. Li, A.-M.; Pollard, T. P.; Wang, Z.; Zhang, N.; Omenya, F.; Tan, S.; Hu, E.; Yang, X.-Q.; Li, X.; Borodin, O.; Wang, C. Non-Fluorinated Electrolyte for High-Voltage Anode-Free Sodium Metal Battery. *Nat. Sustain.* **2026,** *9*, 306-316.

20. Liu, S.; Tang, S.; Zhang, X.; Wang, A.; Yang, Q. H.; Luo, J. Porous Al Current Collector for Dendrite-Free Na Metal Anodes. *Nano Lett.* **2017,** *17,* 5862-5868.

21. Zhang, Y.; Wu, F.; Lv, Z.; Chen, Y.; Wang, W.; Dong, M.; Su, Y.; Xie, M. Unlocking Anode-Free Sodium Metal Batteries Via Solvent Co-Insertion Mediated In Situ Sodiophilic Interface Engineering. *Energy Environ. Mater.* **2025,** *9*, e70112.

22. Cohn, A. P.; Muralidharan, N.; Carter, R.; Share, K.; Pint, C. L. Anode-Free Sodium Battery Through In Situ Plating of Sodium Metal. *Nano Lett.* **2017,** *17*, 1296-1301.

23. Ruan, J.; Hu, J.; Li, Q.; Luo, S.; Yang, J.; Liu, Y.; Song, Y.; Zheng, S.; Sun, D.; Fang, F.; Wang, F. Current Collector Interphase Design for High-Energy and Stable Anode-Less Sodium Batteries. *Nat. Sustain.* **2025,** *8*, 530-541.

24. Huang, J.; An, X.; Cheng, Z.; Jiang, J.; Bai, Y.; Liu, H. K.; Peng, J.; Wu, M.; Dou, S. X.; Sun, X.; Wu, C. Molecular Wedge Reconstructing the Solvation Structure for Low-Temperature Ah-Level Anode-Free Sodium Metal Batteries. *J. Am. Chem. Soc.* **2025,** *147*, 46594-46607.

25. Zhu, Q.; Yu, D.; Chen, J.; Cheng, L.; Tang, M.; Wang, Y.; Li, Y.; Yang, J.; Wang, H. A 110 Wh kg^{-1} Ah-Level Anode-Free Sodium Battery at -40°C. *Joule* **2024,** *8*, 482-495.

26. Xu, Z.; Lin, C.; Qiu, J.; Wang, Z. Polymer-Regulated Solvation and Interphase Engineering for Long-Life and Safe Quasi-Solid-State Anode-Free Sodium Batteries. *Adv. Mater.* **2025,** *37*, 2506037.

27. Sun, C.; Li, Y.; Sun, Z.; Yuan, X.; Jin, H.; Zhao, Y. Ferroelectric Interface for Efficient Sodium Metal Cycling in Anode-Free Solid-State Batteries. *Mater. Today* **2024,** *80*, 395-405.

28. Ge, J.; Ma, C.; Zhang, Y.; Ma, P.; Zhang, J.; Xie, Z.; Wen, L.; Tang, G.; Wang, Q.; Li, W.; Guo, X.; Guo, Y.; Zhang, E.; Zhang, Y.; Zhao, L.; Chen, W. Edge Electron Effect Induced High-Entropy SEI for Durable Anode-Free Sodium Batteries. *Adv. Mater.* **2025,** *37*, 2413253.

29. Shi, J.; Wang, D.; Liu, Q.; Yu, Z.; Huang, J. Q.; Zhang, B. Intermetallic Layers with Tuned Na Nucleation and Transport for Anode-Free Sodium Metal Batteries. *Nano Lett.* **2025,** *25*, 1800-1807.

30. An, Y.; Pei, Z.; Luan, D.; Lou, X. W. Growing Sodiophilic ZnO Nanorod Arrays on Al Substrate for High-Energy-Density Anode-Free Na Batteries. *J. Am. Chem. Soc.* **2025,** *147*, 46440-46448.

31. Tang, F.; Yang, Y.; Liu, C.; Yang, S.; Xu, S.; Yao, Y.; Yang, H.; Yang, Y.; He, S.; Pan, H.;

Rui, X.; Yu, Y. Initially Anode-Free Sodium Metal Battery Enabled by Strain-Engineered Single-Crystal Aluminum Substrate with (100)-Preferred Orientation. *Nat. Commun.* **2025,** *16*, 2280.

32. Cai, Z.; Tang, F.; Yang, Y.; Xu, S.; Xu, C.; Liu, L.; Rui, X. A Multifunctional Super-Sodiophilic Coating on Aluminum Current Collector for High-Performance Anode-Free Na-Metal Batteries. *Nano Energy* **2023,** *116*, 108814.
33. Liu, L.; Cai, Z.; Yang, S.; Yang, Y.; Yao, Y.; He, S.; Xu, S.; Wu, Z.; Pan, H.; Rui, X.; Yu, Y. Multifunctional High-Entropy Alloy Nanolayer Toward Long-Life Anode-Free Sodium Metal Battery. *Adv. Mater.* **2025,** *37*, 2413331.
34. Zhang, G.; Li, S.; Huang, Q.; Ma, C.; Su, J.; Fu, C.; Huo, H.; Yin, G.; Ma, Y. Tailoring Hard-Base Sites on the Al Current Collector Enables Anode-Free Sodium-Metal Batteries. *ACS Energy Lett.* **2025,** *10*, 4410-4417.
35. Lee, K.; Lee, Y. J.; Lee, M. J.; Han, J.; Lim, J.; Ryu, K.; Yoon, H.; Kim, B. H.; Kim, B. J.; Lee, S. W. A 3D Hierarchical Host with Enhanced Sodiophilicity Enabling Anode-Free Sodium-Metal Batteries. *Adv. Mater.* **2022,** *34*, 2109767.
36. Xu, P.; Huang, F.; Liu, Z.; Guo, S.; Liang, S.; Fang, G. In Situ Construction of NaF-rich Solid Electrolyte Interphase with Metallic Ce Sites for Stable Anode-Free Sodium Metal Batteries. *Angew. Chem. Int. Ed.* **2025,** *64*, e202515566.
37. Li, H.; Zhang, H.; Wu, F.; Zarrabeitia, M.; Geiger, D.; Kaiser, U.; Varzi, A.; Passerini, S. Sodiophilic Current Collectors Based on MOF-Derived Nanocomposites for Anode-Less Na-Metal Batteries. *Adv. Energy Mater.* **2022,** *12*, 2202293.
38. Li, T.; Sun, J.; Gao, S.; Xiao, B.; Cheng, J.; Zhou, Y.; Sun, X.; Jiang, F.; Yan, Z.; Xiong, S. Superior Sodium Metal Anodes Enabled by Sodiophilic Carbonized Coconut Framework with 3D Tubular Structure. *Adv. Energy Mater.* **2020,** *11*, 2003699.
39. Zhuang, R.; Zhang, X.; Qu, C.; Xu, X.; Yang, J.; Ye, Q.; Liu, Z.; Kaskel, S.; Xu, F.; Wang, H. Fluorinated Porous Frameworks Enable Robust Anode-Less Sodium Metal Batteries. *Sci. Adv.* **2023,** *9*, eadh8060.
40. Li, S.; Zhu, H.; Liu, Y.; Wu, Q.; Cheng, S.; Xie, J. Space-Confined Guest Synthesis to Fabricate Sn-Monodispersed N-Doped Mesoporous Host Toward Anode-Free Na Batteries. *Adv. Mater.* **2023,** *35*, 2301967.

41. Xu, P.; Liu, Y.; Qin, M.; Huang, F.; Liang, S.; Fang, G. Electronic Structure Regulation Inducing Robust Solid Electrolyte Interphase for Stable Anode-Free Sodium Metal Batteries. *Adv. Powder Mater.* **2025,** *4*, 100303.

42. Zhu, H.; Peng, L.; Wu, J.; Li, S.; Wu, Q.; Cheng, S.; Xie, J.; Lu, J. Fluorine-Doped Micropore-Covered Mesoporous Carbon Nanofibers for Long-Lasting Anode-Free Sodium Metal Batteries. *Nat. Commun.* **2025,** *16*, 5494.

43. An, Y.; Pei, Z.; Luan, D.; Lou, X. W. Foldable Anode-Free Sodium Batteries Enabled by N,P-Codoped Carbon Macroporous Fibers Incorporated with CoP Nanoparticles. *Sci. Adv.* **2025,** *11*, eadv2007.

44. Guo, W.; Liu, X.; Mu, Y.; Yue, G.; Liu, J.; Zhu, K.; Cui, Z.; Wang, N.; Chen, Z.; Zhao, Y. Outside-In Directional Sodium Deposition Through Self-Supporting Gradient Fluorinated Magnesium Alloy Framework Toward High-Rate Anode-Free Na Batteries. *Energy Storage Mater.* **2024,** *73*, 103840.

45. Zhang, R.; Zhu, X.; Xie, T.; Jiang, C.; Ma, J.; Xie, C.; Ji, H.; Wang, J.; Li, H.; Wang, H. N,O Co-Doped Carbon Spheres Enable Stable Anode-Less Sodium Metal Batteries. *Small Methods* **2025,** *9*, 2401884.

46. Wang, Z.; Tian, R.; Jiang, H.; Chen, G.; Shen, Z.; Zhao, C. Z.; Du, F.; Zhang, Q. A *k* Descriptor to Design of Current Collectors for Anode-Free Sodium Batteries. *Adv. Mater.* **2025,** *37*, 2504760.

47. Zhou, S.; Sun, Z.; Zhang, J.; Yang, H.; Sun, Z.; Zhang, Q.; Wen, S.; Chen, H.; Yin, Q.; Feng, S.; Han, J.; Zeng, L.; Chen, W.; Li, J.; Zhang, L.; Peng, D. L.; Zhang, Q. Single-Atom Engineering for Synergistic Nucleation and Interfacial Regulation Enabling Durable Anode-Free Sodium Metal Batteries. *Adv. Mater.* **2026**, *38,* e13154.

48. Wang, Y.; Dong, H.; Katyal, N.; Hao, H.; Liu, P.; Celio, H.; Henkelman, G.; Watt, J.; Mitlin, D. A Sodium-Antimony-Telluride Intermetallic Allows Sodium-Metal Cycling at 100% Depth of Discharge and as an Anode-Free Metal Battery. *Adv. Mater.* **2022,** *34*, 2106005.

49. Wu, S.; Hwang, J.; Matsumoto, K.; Hagiwara, R. The Rational Design of Low-Barrier Fluorinated Aluminum Substrates for Anode-Free Sodium Metal Battery. *Adv. Energy Mater.* **2023,** *13*, 2302468.

50. Wang, C.; Zheng, Y.; Chen, Z. N.; Zhang, R.; He, W.; Li, K.; Yan, S.; Cui, J.; Fang, X.; Yan,

J.; Xu, G.; Peng, D.; Ren, B.; Zheng, N. Robust Anode-Free Sodium Metal Batteries Enabled by Artificial Sodium Formate Interface. *Adv. Energy Mater.* **2023,** *13*, 2204125.

51. Yuan, H.; Yu, D.; Ding, P.; Liu, H.; Wen, K.; Ren, X.; Li, Y.; Liang, Y.; Guo, C.; Zhang, J.; Ren, Y.; Zhao, C. Z.; Li, L.; Yang, Y.; Zhang, Q.; Nan, C. W. Regulating Sodium Deposition Kinetics to Decouple the Electrochemo-Mechanical Effects in Anode-Free Sodium Batteries. *Adv. Energy Mater.* **2025,** *15*, 2501103.

52. Lu, Z.; Yang, H.; Yang, Q. H.; He, P.; Zhou, H. Building a Beyond Concentrated Electrolyte for High-Voltage Anode-Free Rechargeable Sodium Batteries. *Angew. Chem. Int. Ed.* **2022,** *61*, e202200410.

53. Li, Y.; Zhou, Q.; Weng, S.; Ding, F.; Qi, X.; Lu, J.; Li, Y.; Zhang, X.; Rong, X.; Lu, Y.; Wang, X.; Xiao, R.; Li, H.; Huang, X.; Chen, L.; Hu, Y.-S. Interfacial Engineering to Achieve an Energy Density of over 200 Wh kg^{-1} in Sodium Batteries. *Nat. Energy* **2022,** *7*, 511-519.

54. Xie, C.; Liu, S.; Wang, J.; Meng, X.; Yu, S.; Zhang, J.; Peng, H.; Sun, D.; Tang, Y.; Wang, H. Sodium-Compensating Electrolyte Additives Stabilize Interfaces for Highly Reversible Anode-Free Sodium Batteries. *Energy Environ. Sci.* **2026,** *19*, 200-210.
